# Current advances in metal nanoparticle-based stimuli-responsive nanotherapies

**DOI:** 10.3389/fchem.2026.1886627

**Published:** 2026-09-11

**Authors:** Yao Wang, Qianpeng Li, Meina Yu, Yao Gao, Kirsten L. Van Landuyt, Wenyue Sun, Huanhuan Jiang

**Affiliations:** 1 Department of Oral Implantology, Wuxi Stomatology Hospital, Wuxi, China; 2 Department of Oral Health Sciences, KU Leuven, BIOMAT & UZ, Leuven, Belgium; 3 Department of Imaging and Pathology, Faculty of Medicine, OMFS IMPATH Research Group, University Hospitals, Leuven, Belgium

**Keywords:** functionalization, metal-based nanomaterials, nanotherapies, photosensitizers, reactive oxygen species

## Abstract

Stimuli-responsive nanotherapy (SRN) has emerged as a promising therapeutic strategy that employs nanosensitizers activated by external physical or chemical stimuli to generate reactive species for disease treatment. Among various nanosensitizers, metal nanoparticles (MNPs) have attracted considerable attention owing to their unique physicochemical properties, including tunable optical responses, catalytic activity, magnetic behavior, and high surface reactivity. These characteristics enable the construction of versatile nanoplatforms for multiple dynamic therapeutic modalities. In this review, we summarize the fundamental physicochemical properties of representative metal nanoparticles and their relevance to stimulus-responsive nanotherapies. We systematically review major therapeutic modalities, including photodynamic therapy (PDT), photothermal therapy (PTT), sonodynamic therapy (SDT), piezocatalytic therapy (PCT), magnetic hyperthermia therapy (MHT), chemodynamic therapy (CDT), and electrodynamic therapy (EDT), with emphasis on their underlying mechanisms, material design, and recent biomedical applications. We further discuss the advantages and limitations of these therapeutic strategies and highlight recent advances in multifunctional nanoplatforms that integrate multiple therapeutic mechanisms to achieve synergistic effects. The biomedical applications of these metal nanoparticle-based therapies in tumor treatment, infection control, neurological disorders, bone tissue regeneration, and cardiovascular diseases are highlighted. Finally, current challenges and future perspectives are addressed, with particular emphasis on biosafety, therapeutic efficiency, stimulus control, multimodal integration, and clinical translation. This review provides an integrated overview of metal nanoparticle-based stimuli-responsive nanotherapies and highlights opportunities for the rational design of multifunctional therapeutic platforms.

## 1 Introduction

**FIGURE 1:**
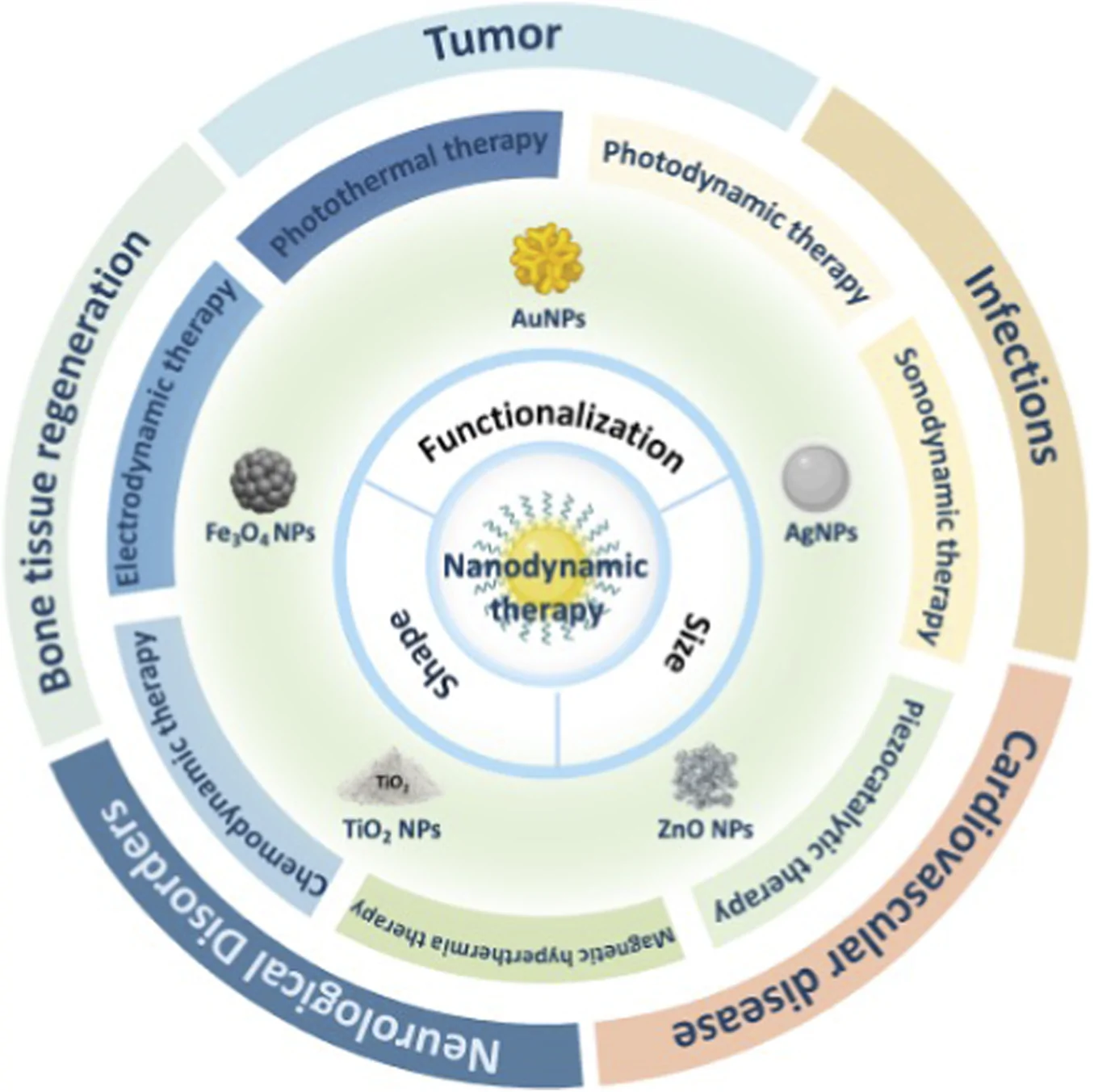
An overview of the construction of metal-based SRNs and their medical applications.

The last quarter century has witnessed substantial impacts from global health issues. The prevalence of diseases such as infections, inflammation, tumors, and other disorders indicates an urgent need for the development of effective therapeutic strategies. Current treatment methods often face limitations, particularly due to inefficient drug delivery and insufficient therapeutic specificity, underscoring the need for more effective and precisely controlled treatments. Recently, stimuli-responsive nanotherapy (SRN) has emerged as a promising therapeutic strategy for various diseases (Figure 1). These therapies utilize specific external or endogenous stimuli to activate therapeutic agents and induce localized therapeutic effects, thereby providing opportunities to improve therapeutic specificity and treatment efficacy while minimizing damage to healthy tissues. One representative example is photodynamic therapy (PDT), which utilizes light to activate photosensitizers (PSs), subsequently generating reactive oxygen species (ROS) that induce the elimination of aberrant cells. The development of PDT has demonstrated the potential of stimulus-responsive therapeutic strategies and has further stimulated the exploration of other stimulus-responsive modalities using diverse physical and chemical stimuli.

However, conventional PDT faces significant limitations, primarily due to the low tissue penetration depth of light. Most clinically approved PSs are activated by visible and ultraviolet (UV) light, which restricts their effects to superficial sites. Hence, other stimulus-responsive therapeutic modalities have been explored, including sonodynamic therapy (SDT), piezocatalytic therapy (PCT), chemodynamic therapy (CDT) and electrodynamic therapy (EDT) (Deng et al., 2023; Gu et al., 2019). These approaches primarily exert their therapeutic effects through the stimulus-induced generation or regulation of ROS or other reactive species and are therefore collectively classified as nanodynamic therapies (NDTs). In addition to these ROS-generating approaches, stimulus-responsive nanotherapies also encompass other therapeutic mechanisms, such as photothermal therapy (PTT) and magnetic hyperthermia therapy (MHT), which utilize photothermal and magnetothermal conversion, respectively, to induce therapeutic effects (Wang W. et al., 2022). The specificity of these external and endogenous stimuli has propelled swift progress in nanomedicine, offering new avenues for treatment.

Furthermore, most traditional PSs face challenges such as undesirable hydrophobicity, high oxygen dependency, low targeting efficiency, and limited bioavailability. These limitations have motivated the development of versatile metal nanoparticle-based platforms, whose unique physicochemical properties enable them to overcome some of these challenges and facilitate the development of diverse stimulus-responsive therapeutic modalities.

Metal nanoparticles (MNPs), including pure metals, metal alloys, and metal oxides, have attracted great attention due to their unique physicochemical properties (Azharuddin et al., 2019). Representative examples include gold nanoparticles (AuNPs), silver nanoparticles (AgNPs), platinum nanoparticles (PtNPs), titanium dioxide (TiO_2_), and iron oxide (Fe_3_O_4_) nanoparticles (IONPs). These metal nanoparticles possess several distinctive advantages that make them attractive for stimulus-responsive nanotherapies (Colombeau et al., 2016; Kwiatkowski et al., 2018; Qi et al., 2019): (1) Plasmonic metals (e.g., Au, Ag) exhibit strong localized surface plasmon resonance (LSPR), enabling highly efficient photon-to-thermal energy conversion; (2) Semiconducting metal oxides (e.g., TiO_2_, ZnO) possess unique bandgap structures that facilitate electron-hole pair separation under specific physical triggers; (3) Transition metal-based nanomaterials (e.g., Fe_3_O_4_) can serve as robust nanozymes to catalyze endogenous H_2_O_2_ into highly toxic hydroxyl radicals (·OH) via Fenton-like reactions, completely bypassing the oxygen-dependency issue; (4) Their nanoscale dimensions can facilitate cellular uptake and, depending on size and surface properties, transport across selected biological barriers; (5) surface functionalization can improve aqueous dispersibility and biological interactions; and (6) conjugation with targeting ligands can enable receptor-mediated targeting (Chen T. et al., 2020; Dirersa et al., 2023; Liang et al., 2023; Meng et al., 2023; Yu et al., 2023; Zhu et al., 2023).

The unique physicochemical properties of MNPs make them versatile platforms for the development of stimulus-responsive nanotherapies. Their high surface-area-to-volume ratios, tunable optical and electronic properties, magnetic responsiveness, and high surface reactivity enable them to interact with diverse external and endogenous stimuli. For example, plasmonic nanoparticles can exhibit LSPR, in which incident light induces coherent oscillations of conduction electrons and generates enhanced local electromagnetic fields (Moskovits, 2008). Such properties make MNPs valuable not only as therapeutic agents or sensitizers, but also as platforms for bio-sensing and bioimaging (Huang et al., 2008; Ou et al., 2021; Xia, 2016). Several MNPs, such as AuNPs and AgNPs, have demonstrated efficacy as sensitizers in different stimulus-responsive therapeutic modalities (Dhanalekshmi et al.,2019; Meng et al., 2022; Sun et al., 2017; Park et al., 2020). However, despite their considerable therapeutic potential, the biological safety of MNPs remains an important concern. Their uptake by the mononuclear phagocyte system, particularly in organs such as the liver and spleen, may result in prolonged retention and potential long-term toxicity. Importantly, the biocompatibility and toxicity of MNPs are strongly influenced by factors such as nanoparticle composition, particle size, and surface modification (Asharani et al., 2011; Bai et al., 2020; Pang et al., 2020; Pinzaru et al., 2018). Therefore, considerable research efforts have focused on optimizing the physicochemical and biological properties of MNPs to improve their therapeutic performance and facilitate their potential clinical translation.

In this review, we discuss the fundamental properties of MNPs and their applications in stimulus-responsive nanotherapies. We systematically summarize the mechanisms and recent advances of major therapeutic modalities, including photothermal therapy (PTT), photodynamic therapy (PDT), sonodynamic therapy (SDT), piezocatalytic therapy (PCT), magnetic hyperthermia therapy (MHT), chemodynamic therapy (CDT), and electrodynamic therapy (EDT), with particular emphasis on the roles of MNPs in stimulus activation and therapeutic regulation. In addition, the biomedical applications of MNP-based stimulus-responsive nanotherapies in tumors, infections, neurological disorders, bone tissue regeneration, and cardiovascular diseases are discussed. Finally, we highlight the current challenges and critical issues associated with MNP-based stimulus-responsive nanotherapies, including biosafety, therapeutic efficiency, stimulus control, and clinical translation, and provide perspectives on the development of multifunctional nanotherapeutic platforms for future biomedical applications.

## 2 Fundamental properties

### 2.1 Size

Nanoparticles within the 1–100 nm size demonstrate size-dependent physicochemical and biological behaviors distinct from their bulk counterparts, which are highly relevant for biomedical applications (Bayda et al., 2019). At the cellular level, transport pathways are intrinsically size-selective: small molecules (<1 nm) cross membranes via channels or transporters, whereas macromolecules (>10 nm) rely on receptor-mediated endocytosis (Xiang et al., 2019). This size-selective biological transport provides a fundamental basis for understanding nanoparticle–cell interactions.

Although reducing nanoparticle size generally improves vascular extravasation and tissue penetration (Hoshyar et al., 2016; Lin et al., 2019; Popović et al., 2010), cellular internalization does not increase monotonically as particle size decreases. For example, Jiang et al. showed that nanoparticles with diameters of approximately 40–50 nm achieved maximal cellular uptake (Jiang et al., 2008), which was attributed to an optimal balance between membrane deformation and endocytic wrapping efficiency (Zhang et al., 2015). A similar nonmonotonic size dependence has also been observed for AgNPs (Chen et al., 2015), indicating that this phenomenon is not restricted to a single nanomaterial system. Collectively, these observations suggest that nanoparticles within an intermediate size regime can retain sufficient transport across biological barriers while still supporting efficient cellular entry (Foroozandeh and Aziz, 2018). Further miniaturization therefore does not necessarily translate into superior therapeutic performance. Although ultrasmall particles may penetrate more deeply into tumors, they are also more rapidly eliminated, resulting in diminished intratumoral retention (Tang et al., 2014). Taken together, these findings indicate that the biological performance of a given nanoparticle population is governed by a trade-off among tissue penetration, cellular internalization, and retention, thereby giving rise to an optimal intermediate size window.

Beyond local transport and cellular uptake, nanoparticle size also critically shapes systemic pharmacokinetics and organ-level biodistribution. Within the size range of ∼10–150 nm, smaller nanoparticles generally display prolonged blood circulation half-lives (Hoshyar et al., 2016; Perrault et al., 2009). Indeed, blood circulation half-life has been reported to scale inversely with particle volume, suggesting that smaller AuNRs can more effectively evade mononuclear phagocyte system (MPS) sequestration and thereby maintain longer systemic exposure (Tong et al., 2016). However, this trend does not extend indefinitely. Ultrasmall nanoparticles (<10 nm), including nanoclusters, are prone to rapid renal clearance (Choi et al., 2011). At the other extreme, larger nanoparticles (>150 nm) are more readily recognized by the immune system, promoting MPS-mediated removal and preferential accumulation in the liver and spleen (Hoshyar et al., 2016).

The size dependence of biodistribution is further illustrated by comparative *in vivo* studies. For instance, Hoshyar et al. compared the variations in biodistribution for 24 and 37 nm diameter shell cross-linked nanoparticles (Hoshyar et al., 2016). The smaller particles showed lower liver accumulation at 10 min post-injection and reduced kidney uptake at 4 h, whereas splenic uptake exhibited no clear correlation with size. Accordingly, particle size should be regarded as a primary design variable in nanomedicine rather than a simple geometric descriptor. Representative examples of size-dependent transport, biodistribution, and cellular uptake are summarized in Table 1. Importantly, no universal optimum exists. The preferred size window must be defined in a context-dependent manner by jointly considering material composition, surface chemistry, target tissue, and therapeutic objective.

**TABLE 1:** Size-dependent biological transport, biodistribution, and cellular uptake behaviors of metal-based nanoparticles.

Size (nm)	Biological behavior	Mechanism	Representative nanosystem	Ref.
<10	Rapid renal clearance	Nanoparticles with hydrodynamic diameters below the glomerular filtration threshold can pass through the renal filtration barrier and be efficiently eliminated via urine	AuNPs, ultrasmall inorganic NPs	Choi et al. (2007), Choi et al. (2011)
∼10–20	Efficient vascular extravasation and deep tissue penetration	Small particle size facilitates transvascular transport and reduces steric hindrance across endothelial barriers	AuNPs	Blanco et al. (2015), Lin et al. (2019), Longmire et al. (2011); Popović et al. (2010)
40–70	Maximum cellular internalization	Optimal membrane wrapping energy and receptor clustering enhance receptor-mediated endocytosis efficiency	AuNPs	Jiang et al. (2008), Zhang et al. (2015)
120–150	Prolonged blood circulation	Reduced renal filtration and moderated recognition by the mononuclear phagocyte system (MPS) prolong systemic circulation	AuNPs, AgNPs, SiO_2_ NPsetc.	Alexis et al. (2008), Hoshyar et al. (2016), Li et al. (2019), Perrault et al. (2009)
>150	Preferential accumulation in liver and spleen	Enhanced opsonization and recognition by macrophages in the reticuloendothelial system (RES) lead to hepatic and splenic sequestration	AgNPs, polymeric NPs	Chen et al. (2015), Wilhelm et al. (2016)

### 2.2 Shape

**FIGURE 2:**
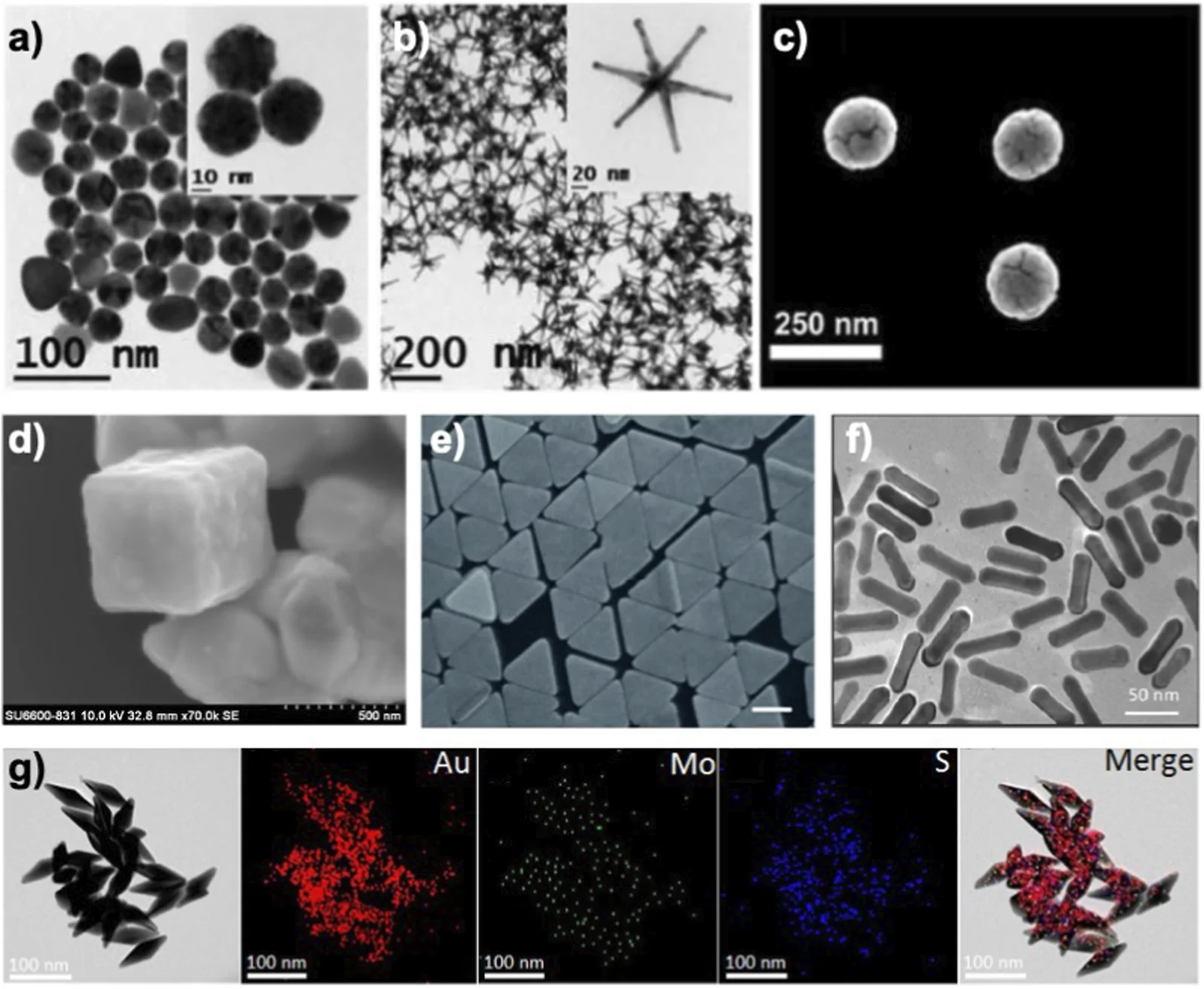
Schematic illustrations of representative nanoparticles with diverse morphologies. **(a)** Spherical-shaped and **(b)** star-shaped TEM images of Au nanoparticles. Reproduced with permission (Atta et al., 2019). Copyright 2019, Royal Society of Chemistry. **(c)** SEM image of Au nanoshells with defective shell morphology. Reproduced with permission (Brinson et al., 2008). Copyright 2008, American Chemical Society. **(d)** SEM images of Au nanocages synthesized using a microwave-assisted approach. Reproduced with permission (Raveendran et al., 2017). Copyright 2017, Springer Nature. **(e)** Au nanotriangles obtained by a seeded growth method. Reproduced with permission (Huang et al., 2014). Copyright 2014, Royal Society of Chemistry. **(f)** Au nanorods prepared by seeded growth method. Reproduced with permission (Huang et al., 2019). Copyright 2019, Wiley. **(g)** EDX elemental mapping of Au nanobipyramids. Reproduced with permission (Maji et al., 2018). Copyright 2018, American Chemical Society.

Various synthetic strategies enable precise control over nanoparticle morphology, including spherical, star-shaped (Atta et al., 2019), nanoshell (Brinson et al., 2008), nanocage (Raveendran et al., 2017), triangular (Huang et al., 2014), rod-like (Huang et al., 2019), and bipyramid structures (Maji et al., 2018). Nanoparticle shape critically governs optical responses, cellular interactions, and intracellular trafficking, thereby influencing therapeutic performance. Figure 2 presents schematic illustrations of representative nanoparticles with diverse morphologies, together with the corresponding references. Tong et al. revealed that tumor accumulation of gold nanorods is more strongly associated with aspect ratio than with particle volume, highlighting the superior performance of elongated nanorod architectures in achieving enhanced intratumoral sequestration (Tong et al., 2016).

Anisotropic nanostructures such as gold nanorods and nanostars exhibit shape-dependent LSPR, enabling tunable near-infrared absorption that is advantageous for photothermal therapy (PTT) and photoacoustic imaging (Zhou R. et al., 2022). In particular, nanostars generate highly localized electromagnetic “hot spots” at their sharp tips, enhancing photothermal conversion efficiency and surface-enhanced Raman scattering (Ma et al., 2020). However, the influence of nanoparticle shape on other therapeutic modalities (e.g., sonodynamic or catalytic therapies) remains insufficiently explored and warrants further systematic investigation.

Beyond optical effects, nanoparticle morphology strongly affects biological interactions. Irregular and anisotropic nanoparticles can exhibit enhanced tissue penetration compared with spherical counterparts (Golbek et al., 2022). However, these shape-dependent effects are closely coupled to particle size, aspect ratio, and surface properties. Particle size influences diffusion, cellular uptake, and tissue transport, whereas the aspect ratio of anisotropic nanoparticles affects their membrane contact, cellular internalization, and biodistribution. Surface charge and surface functionalization further modulate protein adsorption and nanoparticle–cell interactions, thereby altering the biological effects associated with a given morphology. Moreover, sharp-edged nanostructures can facilitate endosomal membrane disruption and cytoplasmic localization, potentially leading to prolonged intracellular retention and reduced exocytosis (Salatin et al., 2015). Nevertheless, these advantages may be accompanied by elevated cytotoxicity; for example, rod-shaped nanoparticles have been reported to induce higher cytotoxic responses than spherical or sheet-like structures under specific experimental conditions (Cheng Y. et al., 2023). Collectively, these findings underscore that therapeutic performance is determined not by morphology alone but by the interplay among shape, size, aspect ratio, and surface properties, highlighting the importance of multidimensional nanoparticle design in balancing therapeutic efficacy and biosafety.

### 2.3 Functionalization

Precise surface functionalization is essential for metal nanoparticles to achieve biological compatibility, colloidal stability, and target specificity in biomedical environments. Small-sized metal NPs such as Au, Ag, Fe, Ti, and Cu demonstrate enhanced catalytic performance owing to their high specific surface area and abundant unsaturated bonds (Song X. R. et al., 2016; Wawrzyńczak et al., 2024). However, their high surface energy makes them prone to aggregation during both preparation and application. Such aggregation significantly reduces the available surface area and the number of active sites, thereby diminishing their catalytic efficiency (Liu et al., 2017).

Beyond colloidal instability, another major challenge in biomedical applications is the formation of a protein corona. In physiological environments, proteins and other biomolecules rapidly adsorb onto nanoparticle surfaces, forming a dynamic protein layer that alters the surface charge distribution and masks functional groups. Depending on their binding affinity and exchange dynamics, adsorbed biomolecules can form relatively persistent “hard” and more dynamically exchanging “soft” corona layers. Importantly, corona formation can redefine the biological identity of functionalized nanoparticles, such that the surface recognized by cells and tissues *in vivo* may differ substantially from the originally engineered surface (Chen D. et al., 2020; Hoang et al., 2022). The composition and dynamics of the protein corona depend on nanoparticle size, shape, surface chemistry, charge, and the surrounding biological environment, and may evolve during circulation and tissue exposure (Bashiri et al., 2023; Hoang et al., 2022). Therefore, not only the amount but also the composition of adsorbed proteins is important for determining nanoparticle behavior. Corona components may shield or alter the accessibility of targeting ligands and influence receptor recognition, cellular uptake, biodistribution, immune recognition, and clearance. Consequently, the biological performance of a functionalized nanoparticle cannot always be predicted solely from its synthetic surface properties (Bashiri et al., 2023; Chen D. et al., 2020; Hoang et al., 2022). To address colloidal and biological-interface challenges, a variety of surface functionalization strategies have been developed, including peptide conjugation, polymer coatings, small-molecule ligands, DNA or glycan functionalization, and dendronization. These modifications enhance nanoparticle stability, targeting capability, and functional versatility, ultimately improving their therapeutic performance (Hoang et al., 2022). However, their biological effects ultimately depend on how the engineered surface interacts with biomolecules after administration, emphasizing the importance of evaluating surface functionalization under physiologically relevant conditions.

Among these strategies, polymer and dendrimer coatings are extensively investigated because their hydrophilicity and steric hindrance can reduce nanoparticle aggregation while improving solubility and dispersibility (Huang et al., 2015). A variety of polymers have been employed for nanoparticle modification, including polyetherimide, polyethylene glycol (PEG), polydopamine, copolymers, polyacrylic acid, polyvinyl alcohol, and chitosan (Bhuckory et al., 2023; Chen Y. et al., 2022; Najim Obaid et al., 2024). PEG is one of the most widely used surface modifiers. PEGylation provides a steric shielding effect that can reduce nonspecific protein adsorption, prolong blood circulation time, and decrease immune clearance of nanoparticles *in vivo* (Alalaiwe et al., 2017; Groysbeck et al., 2023). Nevertheless, PEGylation should not be regarded as completely “non-fouling.” Protein adsorption can still occur on PEGylated nanoparticles, and the resulting corona composition may modify ligand accessibility, cellular interactions, biodistribution, and clearance (Bashiri et al., 2023; Suk et al., 2016). Thus, the biological performance of PEGylated nanoparticles depends not only on reduced total protein adsorption but also on the identity and organization of the proteins that remain associated with the nanoparticle surface. In addition, pre-existing or treatment-induced anti-PEG antibodies have been associated with accelerated blood clearance (ABC) and complement-mediated immune responses, potentially reducing the circulation time and therapeutic efficacy of PEGylated nanomedicines, particularly after repeated administration (Chen B. M. et al., 2021; Ishida et al., 2003). Typically, one end of the PEG chain is chemically anchored to the nanoparticle surface, while the other terminus can be further functionalized with targeting ligands, fluorescent probes, or stimuli-responsive groups, enabling versatile functional customization.

For example, Basti et al. developed multidentate catechol and PEGylated polymers-coated Fe_3_O_4_ nanoparticles to improve the magnetic resonance imaging (MRI) performance (Basti et al., 2010). In addition to improving colloidal stability, polymer coatings can also serve as platforms for conjugating targeting ligands, thereby enhancing the specificity of nanoparticle-based drug delivery. Beyond passive stabilization, polymer coatings can also enable stimuli-responsive therapeutic systems. For instance, Yavuz et al. developed a pH-sensitive polymer-based drug-loaded gold nanocages (Wang et al., 2024). Upon light activation, the temperature of the nanoparticles rises, causing the polymer to melt and expose the pores of the metal cages. This enables the controlled release of the encapsulated drug. By altering the coating agents, similar strategies can be used to create intelligent NPs that respond to various stimuli, including pH, acoustics, and electric fields.

In addition to steric stabilization, electrostatic interactions also play a crucial role in nanoparticle–cell interactions. Surface charge can significantly influence the binding affinity between nanoparticles and biological membranes. Most metal nanoparticles possess a negative surface charge in aqueous environments, while eukaryotic cell membranes are also negatively charged due to the presence of glycoproteins and phospholipids. This electrostatic repulsion can hinder effective nanoparticle–cell interactions. To overcome this limitation, Wiwatchaitawee et al. encapsulated nanoparticles with positively charged polymers, such as poly (amidoamine) and poly (ethylenimine), thereby increasing nanoparticle interactions with glioblastoma multiforme cells and improving therapeutic efficacy (Wiwatchaitawee et al., 2022).

Furthermore, biomolecule-derived coatings have also emerged as promising surface engineering strategies. Biological components such as cell membranes, nucleic acids, proteins, cytokines, and peptides have been used to construct biomimetic nanoplatforms (Li X. et al., 2024; Liang and Chen, 2020; Song X. R. et al., 2016). For example, Li et al. functionalized silica nanoparticles with activated platelet membranes and conjugated them with tumor necrosis factor-related apoptosis-inducing ligand (Li et al., 2016). This approach enabled nanoparticles to selectively target and kill tumor cells in the bloodstream, offering a promising strategy for targeted cancer therapy. Nevertheless, the biological behavior of functionalized nanoparticles cannot be predicted solely from their engineered surface properties. Following exposure to biological fluids, biomolecular adsorption and protein-corona formation can dynamically redefine nanoparticle biological identity, potentially altering ligand accessibility, targeting efficiency, biodistribution, and clearance. Moreover, despite favorable *in vitro* performance, the long-term stability, degradation, metabolism, and immunological consequences of many surface modifiers remain insufficiently understood. Surface coatings may also increase the hydrodynamic diameter of nanoparticles, potentially impairing transport across physiological barriers. Future surface-engineering strategies should therefore consider both the intended synthetic properties and the biological identity acquired under physiological conditions, with comprehensive *in vivo* evaluation required to support clinical translation.

## 3 Representative metallic nanoparticles

### 3.1 Gold nanoparticles

AuNPs have been extensively explored in biomedical applications because of their favorable physicochemical properties and the possibility of achieving acceptable biocompatibility under appropriately controlled conditions (Liu et al., 2013). For example, a zebrafish study showed that 15–35 nm AuNPs accumulated in embryonic cells without detectable cytotoxic effects during embryonic development (Asharani et al., 2011). AuNPs have therefore also been investigated as surface coatings for more reactive nanoparticles to improve their biocompatibility. Liu et al. prepared Au/Fe nanoparticles with a gold-to-iron atomic ratio of 1:10, which significantly reduced cytotoxicity while preserving their magnetic properties (Liu et al., 2013). Similarly, Au/Ag nanoparticles demonstrated a lower toxicity profile than monometallic AgNPs (Singh et al., 2022). However, the biological response to AuNPs is highly dependent on their physicochemical characteristics and exposure conditions, including particle size, morphology, surface chemistry, aggregation state, dose, and exposure duration. In particular, particle size can substantially influence cellular uptake, biodistribution, barrier penetration, and biological activity (Sardar et al., 2009). Therefore, the biocompatibility of AuNPs should be evaluated in a formulation- and exposure-specific manner rather than regarded as an intrinsic property of gold nanomaterials.

AuNPs possess unique, morphology-dependent optical properties that are particularly advantageous for tumor therapy. Conventional spherical AuNPs typically exhibit an LSPR absorption band around 520 nm, whereas anisotropic nanostructures such as gold nanorods, nanoshells, nanocages, and nanostars can be engineered to shift their plasmonic absorption toward the red and near-infrared (NIR) regions, thereby improving their suitability for light-triggered therapeutic applications (Yang Y. et al., 2015). The major therapeutic mechanisms associated with these optical properties include the following:

1. Photothermal effect.

When the excitation wavelength overlaps with the LSPR band of AuNPs, the conduction electrons at the nanoparticle surface undergo collective oscillation, resulting in enhanced interaction with the incident light (Peng et al., 2023). The position and intensity of the LSPR band are influenced by nanoparticle size, morphology, composition, and the surrounding dielectric environment. As a result, AuNPs exhibit enhanced light absorption and scattering around their LSPR band. The relative contributions of absorption and scattering depend strongly on nanoparticle size and morphology; smaller AuNPs generally exhibit absorption-dominated optical responses, whereas larger particles tend to show an increased contribution from scattering (Ahmad et al., 2020; Huang et al., 2007). For photothermal applications, light absorption is particularly important because the absorbed optical energy is converted into heat through non-radiative relaxation processes, whereas light scattering primarily redirects incident photons and contributes more substantially to optical imaging (Huang and El-Sayed, 2010). Consequently, AuNPs with strong absorption in the desired spectral region can efficiently convert light energy into localized heat, resulting in thermal damage to tumor tissue (Chen et al., 2016; Jabeen et al., 2014; Yang H. et al., 2015).

2. Reactive oxygen species generation.

Excited AuNPs can also interact with surrounding molecules and promote the generation of ROS, which may contribute to tumor tissue damage. Although AuNPs can also serve as photodynamic agents, their application in photodynamic therapy is less common than their well-established use in photothermal therapy (Hwang et al., 2014).

3. Enhancement of photodynamic therapy.

Upon light excitation, plasmonic coupling between AuNPs and nearby photosensitizers can enhance local electromagnetic fields and potentially improve photosensitizer excitation. Consequently, AuNPs are often used in combination with photosensitizers in PDT to boost its effectiveness. For example, Au nanorods have been shown to significantly enhance the photodynamic therapy of methylene blue (Chen et al., 2016).

Notably, both PDT and PTT typically rely on near-infrared light, which enables deeper tissue penetration (Song J. et al., 2016). Conventional spherical AuNPs exhibit LSPR near 520 nm, whereas anisotropic Au nanostructures can be engineered for absorption within the NIR windows (Lorenzana-Vázquez et al., 2023). Alternatively, upconversion nanoparticles have been widely integrated with AuNPs to convert NIR light into visible light, thereby significantly improving PDT efficiency (Chen et al., 2016). In addition, AuNPs can function as sensitizers under various external stimuli. For instance, ultrasound (US) can activate AuNPs for targeting melanoma cells (Zahraie et al., 2022) and peri-implant infections (Sun et al., 2022). Hydrogen peroxide (H_2_O_2_) can also generate ROS through catalysis by AuNPs (Ouyang et al., 2022), while low-dose X-rays can induce ROS production in gold nanoclusters via an oxygen-independent Type I pathway (Zhu et al., 2023). However, the mechanisms behind these emerging nanodynamic therapies are not fully understood and warrant further investigation.

Despite their considerable biomedical potential, the clinical application of AuNPs as sensitizers still faces several challenges, including relatively limited ROS generation efficiency and insufficient selectivity toward pathological tissues. Therefore, further structural engineering and surface modification of AuNPs are required to improve their therapeutic efficacy and clinical translation potential.

### 3.2 Silver nanoparticles

AgNPs possess similar optical properties to those of AuNPs, allowing them to absorb NIR light and produce ROS and/or heat (Park et al., 2020). However, unlike AuNPs, AgNPs are less commonly used alone as photosensitizers in PTT due to their higher bioreactivity.

The stability of AgNPs is highly dependent on coating agents noncovalently attached to their surface. These coatings can be displaced or weakened by competing molecules within biological tissues, causing AgNPs to lose their electrostatic or steric barriers, leading to NPs’ aggregation (McShan et al., 2014). Consequently, their photothermal conversion efficiency declines. Furthermore, uncoated AgNPs are prone to oxidation by oxygen molecules, forming silver oxide and releasing cytotoxic Ag^+^ ions (McShan et al., 2014). Therefore, improving stability and reducing cytotoxicity are key modification strategies, such as employing more stable metal coatings or durable coating agents (Ma et al., 2024; Meng et al., 2020).

Notably, AgNPs are widely used as antibacterial agents in SRN due to their inherent biotoxicity. Beyond producing ROS, AgNPs exert antibacterial effects through multiple mechanisms:

1. Membrane interactions: AgNPs or silver ions can adsorb onto bacterial cell membranes, interacting with extracellular proteins, altering membrane potential (Quinteros et al., 2018), promoting bacterial cell death (Schaumann et al., 2015) and disrupting the membrane structure (El Badawy et al., 2011). Bacterial cell surfaces carry a more negative charge compared to mammalian cells, primarily due to the presence of ionized phosphate and carboxyl groups unique to bacteria. This further favors the binding of AgNPs and Ag^+^ ions (Wilhelm et al., 2021; Zhang et al., 2019).
2. Intracellular effects: AgNPs and released Ag^+^ can enter or interact with bacterial cells following membrane disruption, leading to oxidative stress, enzyme inhibition, and disruption of intracellular metabolic processes (McShan et al., 2014).
3. DNA interactions: AgNPs can bind to sulfur and phosphorus groups in DNA, altering its conformation and causing DNA damage (Ali et al., 2023; Lim et al., 2012). The strong affinity between AgNPs and DNA has also led to their extensive application in DNA biosensing (Fang et al., 2018; Wu et al., 2020).
4. Protein interactions: AgNPs can form covalent bonds with sulfhydryl groups and interact with acidic amino acid residues or phosphorylation sites via electrostatic interactions (Liao et al., 2019). These interactions can alter protein structure and function, disrupting crucial signaling pathways in microorganisms (McShan et al., 2014).

Similar to AuNPs, AgNPs can also be activated by various stimuli, including light, radiation, and chemical substances. Firstly, compared to AuNPs, AgNPs exhibit a broader surface plasmon resonance range (300–1,200 nm), spanning from ultraviolet to NIR regions (Kah et al., 2023). However, their absorption peak occurs at a shorter wavelength of 420–440 nm (Srividhyaa et al., 2023), compared to the approximately 520 nm peak of AuNPs (Lorenzana-Vázquez et al., 2023). Because the intrinsic plasmonic absorption of conventional AgNPs is predominantly located in the visible region, direct optical activation generally provides more limited tissue penetration than excitation within the NIR biological windows. Under NIR light irradiation, AgNPs can generate localized hyperthermia and ROS, enabling PTT and/or PDT against tumors and infections and thereby extending their therapeutic potential to lesions located beneath superficial tissues (Li Y. et al., 2022; Zhang et al., 2020). Besides, AgNPs can enhance the activation of chemical photosensitizers, such as toluidine blue and meso-tetrakis (N-methyl-4-pyridinio)porphyrin under NIR light, making them effective carriers for photosensitizers and improving PDT efficiency (Aydın et al., 2020; Malá et al., 2021). Secondly, AgNPs have shown potential as radiosensitizers in radiotherapy for various cancers, including glioma (Wu et al., 2016), nasopharyngeal carcinoma (Zhao et al., 2012) and renal cell carcinoma (Morais et al., 2023). Thirdly, AgNPs are pH-responsive, as they release Ag^+^ ions in the presence of protons (H^+^), which can react with H_2_O_2_ to generate ROS or bind to negatively charged molecules in bacteria (He et al., 2023). Given the features of H_2_O_2_ enrichment and low pH in tumor microenvironment (TME) (Fu et al., 2021) and infected tissue (Zhou K. et al., 2022), chemodynamic therapy based on AgNPs emerges as a promising treatment strategy, especially when combined with PTT (Zhou K. et al., 2022). Finally, recent studies suggested the potential of AgNPs as sonosensitizers in SDT. For instance, Myronov et al. demonstrated that low-frequency US enhanced the effectiveness of silver nanoparticles in a purulent wound model (Myronov et al., 2020). Similarly, Meng et al. found that AgNPs could accelerate the ultrasound response of TiO_2_ nanoparticles (Zhou K. et al., 2022).

In summary, compared to AuNPs, AgNPs exhibit relatively strong surface plasmon resonance effects, shorter absorption peak wavelengths, and superior antibacterial properties. Therefore, AgNPs -based SRNs are particularly effective for fighting against bacterial infections in superficial tissues. However, the stability and biosafety of AgNPs remain critical challenges. Future research should prioritize improving material safety and reducing toxicity through modifications or integration with biocompatible components.

### 3.3 Iron oxide nanoparticles

IONPs have been widely explored in multifunctional medical applications including antibacterial, anticancer, and cell-labeling activities, owing to their favorable biocompatibility, biodegradability, catalytic activity, and unique magnetic properties. Representative IONPs mainly include Fe_3_O_4_, α-Fe_2_O_3_, and γ-Fe_2_O_3_ nanoparticles (Noqta et al., 2019). At the nanoscale, iron oxides exhibit physicochemical properties that differ substantially from those of their bulk counterparts. For example, Fe_3_O_4_ nanoparticles below approximately 20 nm can exhibit superparamagnetic behavior above their blocking temperature, owing to rapid thermally induced fluctuations of their magnetic moment (Li et al., 2017). This property enables remote manipulation by external magnetic fields and has supported their development for magnetic targeting, molecular imaging, and image-guided therapy.

The magnetic responsiveness of IONPs can facilitate their accumulation at target sites before activation of a therapeutic modality. Shen et al. demonstrated that Fe_3_O_4_@carboxymethyl chitosan NPs could be magnetically directed towards tumors, thereby improving the efficiency of photothermal ablation (Shen et al., 2013). IONPs can also function as MRI contrast agents, particularly by producing signal attenuation in T2-and T2*-weighted imaging, allowing nanoparticle distribution to be monitored before or during treatment. Caro et al., for example, developed Fe_3_O_4_@Au nanoparticles for imaging-guided photothermal therapy in rodent models (Caro et al., 2021). Similarly, Zhang et al. integrated a porphyrin-based MOF photosensitizer with an Fe_3_O_4_@C core, in which the porphyrinic component mediated PDT, whereas the Fe_3_O_4_ core provided magnetic responsiveness and MRI capability, enabling multimodal imaging-guided therapy (Zhang et al., 2017). These findings highlight the role of IONPs as magnetically responsive and imaging-enabled components that can facilitate the spatial control and monitoring of stimuli-responsive nanotherapies.

More directly relevant to stimuli-responsive nanotherapies is the intrinsic catalytic activity of IONPs. Through Fe^2+^/Fe^3+^ redox cycling, iron-based nanomaterials can catalyze Fenton or Fenton-like reactions that convert H_2_O_2_ into highly reactive hydroxyl radicals (•OH), thereby inducing oxidative damage in diseased tissues (Guo et al., 2022; Zhang et al., 2025). Because elevated H_2_O_2_ levels are frequently associated with tumor and inflammatory or infected microenvironments, this catalytic activity provides a mechanistic basis for using IONPs in ROS-mediated therapeutic strategies. In this context, IONPs can function as catalytic components of stimuli-responsive therapeutic platforms, enabling microenvironment-responsive ROS generation rather than serving merely as passive magnetic carriers. Beyond these ROS-mediated effects, IONPs have also been reported to regulate osteogenesis-related signaling pathways, including Wnt/β-catenin (Xia et al., 2019), PI3K/Akt (Song et al., 2017), and ERK-MAPK (Yu et al., 2020), while suppressing osteoclast activity and bone loss (Liu L. et al., 2020). Although these regenerative effects are mechanistically distinct from ROS-mediated therapeutic mechanisms, they may provide additional value when IONP-based therapeutic platforms are developed for bone-related diseases or tissue engineering.

Another important property of IONPs is their ability to generate heat under an alternating magnetic field, forming the basis of MHT (Saeed et al., 2018). Magnetic heating provides an additional mechanism for exploiting the magnetic responsiveness of IONPs in stimuli-responsive nanotherapies. Dan et al. reported that exposure to an alternating magnetic field enhanced the transport of IONPs across the blood–brain barrier by increasing paracellular permeability, with effects extending beyond bulk temperature changes (Dan et al., 2015). However, intracellular internalization of IONPs can reduce their magnetic properties and heat-generation efficiency (Kolosnjaj-Tabi et al., 2014). To address intracellular limitations, Creixell et al. developed a novel nano-ionic delivery method that targets the epidermal growth factor receptor, directing IONPs to lysosomes and improving their heat generation capacity for tumor destruction (Creixell et al., 2011). These findings demonstrate that magnetic stimulation can be exploited not only for therapeutic heating but also for regulating nanoparticle transport, localization, and biological responses.

IONPs can additionally be integrated with photoactive components to combine magnetic targeting with light-triggered ROS production. Because bare IONPs generally exhibit relatively limited intrinsic photochemical ROS generation, they are more commonly employed as catalytic or magnetic components of hybrid nanoplatforms than as primary photosensitizers. Conjugation with organic photosensitizers or other photoactive nanomaterials can therefore introduce complementary PDT or photothermal functions while retaining magnetic guidance and imaging capabilities. For example, Penon et al. showed that porphyrin-functionalized IONPs enhanced singlet oxygen (^1^O_2_) generation (Penon et al., 2016), whereas Unterweger et al. developed hypericin-loaded IONPs that combined magnetic drug targeting with light-triggered photodynamic activity (Unterweger et al., 2015). Such hybrid systems illustrate how magnetic enrichment, catalytic radical generation, and photoinduced ROS production can be integrated within a single nanoscale platform. Overall, the major value of IONPs in stimuli-responsive nanotherapies lies not simply in their capacity to generate heat, but in their ability to integrate Fe-mediated catalytic ROS generation with magnetic targeting, imaging, and externally controlled therapeutic functions. Their capacity to combine Fenton/Fenton-like chemistry with magnetic and photodynamic modalities makes IONPs particularly attractive as multifunctional components for spatially controlled and multimodal stimuli-responsive nanotherapies.

### 3.4 Titanium dioxide nanoparticles

Titanium dioxide (TiO_2_) is an inorganic compound characterized by high chemical stability. Upon UV light irradiation in aqueous environments, TiO_2_ can rapidly generate a variety of ROS, including H_2_O_2_, •OH, ^1^O_2_, and superoxide radicals, through photocatalytic reactions (Wang et al., 2014). This strong photocatalytic oxidation capability has enabled the widespread application of TiO_2_ nanoparticles in biomedical fields such as drug delivery systems, dentistry, and surgical bioengineering.

Despite these advantages, the clinical application of TiO_2_ NPs faces several challenges. TiO_2_ nanoparticles tend to aggregate when dispersed in aqueous solutions. Such aggregation reduces their effective surface area and consequently decreases photocatalytic activity. Another important limitation is the shallow penetration of UV light in biological tissues because of strong optical attenuation, together with its potential phototoxicity. Therefore, improving the responsiveness of TiO_2_ NPs to visible or near-infrared (NIR) light has become an important research focus (Younis et al., 2023). For example, Yang et al. developed a system in which upconversion nanoparticles were used as a substrate and coated with TiO_2_. These upconversion nanoparticles convert NIR light into shorter-wavelength UV light, thereby enhancing the efficacy of photodynamic therapy (PDT) under NIR irradiation.

TiO_2_ nanoparticles are also widely recognized as effective sonosensitizers in sonodynamic therapy (SDT). Ultrasound stimulation can activate TiO_2_ NPs to generate ROS through cavitation and sonoluminescence effects, as well as through the formation of electron–hole pairs. Ultrasound-activated TiO_2_ NPs have been shown to induce mechanical and thermal damage to bacterial cells (Cheng S. et al., 2023; Sun et al., 2022). Furthermore, due to the superior tissue penetration of ultrasound, SDT is considered more suitable than PDT for the treatment of deep-seated lesions.

Even in the absence of external physical activation, TiO_2_ nanoparticles exhibit certain antibacterial properties. TiO_2_ NPs have been shown to inhibit a wide range of microorganisms, including *Escherichia coli*, *Staphylococcus aureus*, *Bacillus cereus*, *Enterococcus faecalis*, *Lactobacillus species*, and *Porphyromonas gingivalis*. Younis et al. summarized the potential antibacterial mechanisms of TiO_2_ NPs, which include ROS generation, ion release, direct contact with bacterial membranes, membrane permeabilization, and interactions with DNA and proteins (Younis et al., 2023).

TiO_2_ nanoparticles have long been regarded as relatively biocompatible and have therefore been widely explored in biomedical applications. However, recent studies have reported potential toxic effects associated with TiO_2_ exposure. TiO_2_ NPs have been shown to induce fatty degeneration in hippocampal regions of the brain, as well as vasodilatation, central vein congestion, and degeneration around the central hepatic vein (Alarifi et al., 2013; Wang et al., 2007; Ze et al., 2014). Moreover, numerous *in vitro* and *in vivo* studies have reported genotoxic effects of TiO_2_ nanoparticles under specific experimental conditions (Frenzilli et al., 2014; Kim et al., 2014; Pawar and Kaul, 2014; Sekar et al., 2014; Srividhyaa et al., 2023; Wang et al., 2015). Consequently, reducing the adverse biological effects of TiO_2_ NPs has become an important research priority. Dorier et al. and Bessa et al. further reported that serum protein-corona formation on TiO_2_ nanoparticles can reduce DNA damage without significantly affecting ROS generation (Bessa et al., 2017; Dorier et al., 2015), illustrating how biological interfaces can further modify nanoparticle toxicity. In summary, TiO_2_ nanoparticles show considerable potential in biomedical applications due to their strong photocatalytic oxidation capacity and broad-spectrum antibacterial properties. However, concerns regarding genotoxicity, biosafety, and their reliance on UV light for optimal activation highlight the need for further investigation. Future studies should focus on improving the safety profile of TiO_2_ NPs and developing strategies that enable efficient activation under safer and more penetrative light sources, such as visible or near-infrared light.

### 3.5 Zinc oxide nanoparticles

Zinc oxide (ZnO) NPs have gained increasing attention as semiconducting materials with excellent photocatalytic, sonocatalytic and piezoelectric properties (Zhang X. et al., 2024). Meanwhile, ZnO has an established safety record for certain regulated applications, including its use as an active ingredient in sunscreen products (Hu et al., 2013). However, the biocompatibility of ZnO NPs is highly dependent on their physicochemical properties, dose, and exposure conditions, and their nanoscale formulation should not be assumed to share the same safety profile as bulk ZnO.

One distinguishing feature of ZnO NPs, compared to other metallic nanoparticles, is their high biodegradability, particularly in low-pH environments. While ZnO NPs generally enter the blood circulation in particle form, they primarily localize in organs as dissolved zinc ions (Choi and Choy, 2014). This natural degradation prevents the accumulation of ZnO NPs in organs like the liver and kidneys, reducing the risk of long-term toxicity. Additionally, zinc ions are essential trace elements for the human body, with a high tolerable upper intake level (40 mg per day), making the degradation of ZnO NPs relatively safe (Seok et al., 2013). However, concerns remain about the toxicity of zinc ions, especially at high concentrations. Seok et al. reported that ZnO NPs with size of 40 nm caused pancreatitis in rats when administered orally, likely due to their dissolution in gastric fluid (Seok et al., 2013). In addition, this degradation may reduce the efficiency of dynamic therapies, particularly in tumor tissues with lower pH levels. These challenges can often be addressed by using capping agents, antidotes, or coatings. For example, chelation with chelators such as ethylene diamine tetra acetic acid disodium salt and N,N,N′,N′-tetrakis-(2-pyridylmethyl) ethylenediamine has been shown to detoxify ZnO NPs (Seok et al., 2013), while Mageswari et al. found that sodium hexametaphosphate (SHMP) capping improved the photocatalytic efficiency of ZnO NPs (Mageswari et al., 2023).

Interestingly, the degradable nature of ZnO NPs also imparts pH-responsive behavior for targeted therapies. Cai et al. developed ZnO@doxorubicin NPs that are taken up by cancer cells. Once inside the acidic environment of endosomes or lysosomes, the ZnO NPs dissolve to release Zn^2+^ ions, which trigger the dissociation of the metal-drug complex and promote controlled release of anticancer drug doxorubicin (Cai et al., 2016). Additionally, the released Zn^2+^ ions can induce pro-death autophagy in cancer cells, further enhancing therapeutic efficacy (Cai et al., 2024). Therefore, the unique biodegradability and pH-responsive behavior of ZnO nanoparticles make them promising candidates for stimuli-responsive nanotherapies.

In summary, different metal nanoparticles exhibit distinct physicochemical properties, which determine their suitability for specific nanotherapeutic modalities. For instance, noble metal nanoparticles such as AuNPs and AgNPs are mainly utilized for photothermal and photodynamic therapies due to their plasmonic optical properties, whereas metal oxides such as Fe_3_O_4_, TiO_2_, and ZnO are more frequently employed in catalytic or magnetically activated therapies. To provide a clearer comparison of these materials, the representative metal nanoparticles and their major applications in different SRNs are summarized in Table 2.

**TABLE 2:** Representative metal nanoparticles and their corresponding applications in different stimuli-responsive nanotherapies.

Material	Size	Shape	Functionalization	Therapy	Stimulation	Disease	Ref.
Au nanoshells (Si@Au)	∼120 nm	Core–shell	PEG	PTT	NIR (820 nm,4 W/cm^2^)	Tumor (mouse)	Hirsch et al. (2003)
Au nanorods	Aspect ratio of 3.9	Rod	Anti-EGFR antibody	PTT	NIR (800 nm,10–20 W/cm^2^)	Nonmalignant epithelial cell line (HaCat); Malignant oral epithelial cell lines (HOC 313 clone 8 and HSC 3)	Huang et al. (2006)
Au nanocages	∼40 nm	Hollow cube	PEG,secondary antibody (anti-mouse immunoglobulin G or IgG)	PTT	NIR (800 nm)	breast cancer cell line (SK-BR-3)	Chen et al. (2005)
Au nanorods	8–90 nm	Rod	Hexadecyltrimethylammonium bromide (CTAB), bovine serum albumin (BSA)	PTT	NIR (1,064 nm,0.35 W/cm^2^)	Breast tumor (mouse)	Zhao et al. (2021)
Au nanostars	70–100 nm	Star	\	PTT	NIR (1,064 nm,0.4 W/cm^2^)	Intracranial tumors (mouse)	Srinivasan et al. (2023)
Au nanoclusters	∼2.7 nm	Cluster	Immune checkpoint inhibitor 1-cyclohexyl-2-(5H-imidazo [5,1-*a*]isoindol-5-yl)ethanol (NLG919)	PTT, PDT	NIR (808 nm,1.5 W/cm^2^)	Tumor (mouse)	Yang et al. (2023)
Au nanorods	∼95 nm	Rod	Ruthenium photosensitizer; Human serum albumin (HSA)	PTT, PDT	NIR (808 nm,0.5–2 W/cm^2^)	Human hepatocellular carcinoma cell line (HepG2)	Shi et al. (2022)
AgNPs	∼40 nm	Sphere	Nano curcumin (nCur); Colistin (CL)	PDT	LED light (450 nm, 0.5 W/cm^2^)	*P. aeruginosa* (ATCC 27853)	Azimzadeh et al. (2024)
Ag plate NPs	∼100 nm	Plate	Ir, IrO2	PTT, PDT	NIR(808 nm, 480 J)	Human cancer cells (MDA-MB-231,HepG2,A375P and HeLa)	Yim et al. (2019)
AgNPs	∼52 nm	Sphere	Photosensitizer	PDT	LED light (620–640 nm, 0.5 W/cm^2^)	Biofilm (*Enterococcus faecalis*)	Aydın et al. (2020)
Ag@Ag_2_O Core-Shell NPs	∼3.8 nm	Core–shell	Black phosphorus vesicle; PEG; Polyacrylic acid (PAA)	PTT	NIR(1,064 nm)	Cancer	Li et al. (2022b)
Au-Ag NPs	∼20 nm	Sphere	PEG; Folic acid (FA); Graphene oxide (GO)	PDT, PTT	NIR(808 nm, 3 W/cm^2^)	Cancer cell lines (A549, AGS, HeLa and HT-29)	Lee et al. (2023)
Fe_3_O_4_ NPs	8,15,20 nm	Sphere	\	MHT	Alternating magnetic field (10 kOe, f = 80, 120 and 180 kHz)	Human cancer cell line (HCT-116)	Attar et al. (2016)
Fe_3_O_4_ clusters	13–17 nm	Multicore	Citric acid	MHT	Magnetic alternating current hyperthermia (f = 950 kHz and H = 10.5 kA/m)	\	Blanco-Andujar et al. (2015)
Fe_3_O_4_@C	7–9 nm	Core–shell DSPE-PEG (2000)	Carbon shell	RDT, CDT	Radio-frequency generator (350 Hz, 1.2 kW); pH 5.5	Pancreatic cancer cell line (Panc-1)	Xu et al. (2012)
Fe–N codoped carbon NPs	∼80 nm	Polyhedral shape and hierarchical porous structure	Sphere	CDT, PTT	H_2_O_2_ solution (0.1 mM)	Tumor cell line (4T1)	Sui et al. (2021)
Fe_3_O_4_@Pt	∼200 nm	Core–shell	Pt shell	CDT, EDT	H_2_O_2_ solution (10–100 mM); Square wave AC electric field (10 MHz, 10 mA)	Tumor (mouse)	Chen et al. (2021c)
Sulfur–Fe–heme NPs	∼63 nm	Sphere	\	CDT	low pH, H_2_O_2_	Esophageal squamous cell carcinoma cell lines (EC109, EC9706, and TE1)	Zhang et al., (2024d)
TiO_2_ NPs	∼120 nm	Sphere	Protein	SDT	Ultrasound (0–0.7 W/cm^2^)	Tumor (mouse)	Ninomiya et al. (2012)
TiO_2_ NPs	∼215 nm	Sphere	Hemoglobin	SDT	Ultrasound (1 MHz, 1 W/cm^2^)	Periodontitis (mouse)	Liang et al. (2014)
Graphene oxide (GR) nanosheets@TiO2	∼230 nm	Sheet	\	SDT, PTT	Ultrasound (0.5–1.5 W/cm^2^); NIR(808 nm, 2 W/cm^2^)	Tumor (mouse)	Dai et al. (2017)
TiO_2_@Au nanoplate	∼80 nm	Plate	PEG	SDT, PTT	Ultrasound (0.5 W/cm^2^, 3 MHz); NIR(1,064 nm, 1 W/cm^2^)	Tumor (mouse)	Gao et al. (2019)
Black TiO_2_	∼50 nm	Defect-rich	nanocomposite hydrogel	SDT, PDT	Ultrasound (1 W/cm^2^, 1 MHz); NIR(808 nm, 1 W/cm^2^)	Bacteria (*S. aureus* and *E. coli*)	Wang et al. (2025b)
ZnO@Ag Nanorods	∼240 nm	Rod	\	SDT	Ultrasound (1.5 W/cm^2^, 1 MHz)	Mouse colorectal carcinoma cell line (CT-26) *F. nucleatum*	Tao et al. (2025)
Fe@Mn@ZnO NP	∼70 nm	Sphere	mPEG-NH_2_	CDT, SDT	0.1 mM H_2_O_2_; Ultrasound (1.5 W/cm^2^, 1 MHz)	Tumor (mouse)	Hu et al. (2022)
ZnO@reduced graphene oxide (rGO) nanosheets@Au	∼200 nm	Hollow Sphere	\	PCT, SDT	Ultrasound (1.5 W/cm^2^, 1 MHz)	Tumor (mouse)	Cai et al. (2024)
Fe_3_O_4_@ZnO	7–10 nm	Sphere	Brusatol	PDT	Ultraviolet A (365 nm, 100 kJ/m^2^)	Tumor (mouse)	Ren et al. (2022)
ZnO_2_	∼150 nm	Sphere	Lip-ICG	PTT	NIR (808 nm,0.5–2 W/cm^2^)	Tumor (mouse)	Wu et al. (2022c)
Pd–Pt nanosheets	190 nm	Sphere	Extracellular vesicles (EVs)	EDT, PTT	NIR (980 nm,0.5–2 W/cm^2^); Square-wave AC field (10 m HZ)	*S. aureus*	Qiao et al. (2022)
Pt	15 nm	Sphere	-	EDT	Square-wave AC (5 mA)	Tumor (mouse)	Gu et al. (2019)
BaTiO3	110 nm	Cube	Injectable thermogel matrix	SDT, PCT	Ultrasound (1 W/cm^2^)	Tumor (mouse)	Zhu et al. (2020)
Cu@BTO	90–150 nm	Rounded cube	Guanidine (Gua) groups	SDT, PCT	Ultrasound (1 MHz, 1 W/cm^2^)	*S. aureus*	Wu et al. (2025)

## 4 Stimulus-responsive nanotherapies

Metal nanoparticle-based stimuli-responsive nanotherapies encompass a diverse range of therapies that exploit distinct external or endogenous stimuli to achieve spatially and temporally controlled therapeutic effects. In this section, we focus on seven representative modalities, including PDT, PTT, SDT, PCT, MHT, CDT, and EDT, with emphasis on their underlying mechanisms, material design, and recent biomedical applications. Despite their shared reliance on stimulus-responsive activation, these modalities differ substantially in their activation requirements, therapeutic mechanisms, and translational constraints. Their principal activation stimuli, mechanisms, advantages, and limitations are comparatively summarized in Table 3.

**TABLE 3:** Comparative characteristics of major stimuli-responsive nanotherapies.

Therapy	Stimulus	Principal mechanism	Advantage	Limitation
PDT	Light	Photoexcitation → Type I/II ROS generation	High spatiotemporal controllability; clinically established	Limited optical penetration; Type II PDT is O_2_-dependent
PTT*	Light, mainly NIR	Photothermal conversion → heat; NDT-relevant when heating promotes radical/ROS generation	Rapid and externally controllable; relatively low O_2_ dependence	Limited optical penetration; overheating may damage surrounding tissues
SDT	Ultrasound	Ultrasound-induced sensitizer activation/cavitation → ROS generation	Greater tissue penetration than optical activation	Mechanisms and efficacy are strongly dependent on ultrasound parameters and tissue properties
PCT	Mechanical stress/ultrasound	Piezoelectric polarization → charge separation → redox reactions/ROS	Can exploit mechanical stimulation; potential for regenerative applications	Requires piezoelectric materials and sufficient mechanical activation
MHT*	Alternating magnetic field	Magnetic relaxation → heat; NDT-relevant when heating promotes radical/ROS generation	Remote activation with relatively deep field penetration	Heating strongly depends on nanoparticle and magnetic-field parameters
CDT	Endogenous chemical microenvironment (e.g., H_2_O_2_, acidic conditions)	Fenton/Fenton-like reactions → •OH	No external energy input; not constrained by external energy penetration	Limited by endogenous H_2_O_2_, pH, and catalytic kinetics
EDT	Electric field/current	Electrically induced redox processes → ROS	Externally controllable; potentially less dependent on endogenous H_2_O_2_	Electrode delivery, electrochemical by-products, and limited clinical evidence

* PTT and MHT are primarily thermal modalities and do not constitute nanodynamic therapy when heat generation alone mediates the therapeutic effect. In the context of NDT, they are considered relevant only when thermal energy triggers or enhances ROS/reactive-radical generation.

### 4.1 Photothermal therapy

The photothermal effect has been widely applied in clinical medicine for disease treatment. Photothermal nanomaterials can convert light energy into heat, thereby locally increasing tissue temperature and eliminating temperature-sensitive cancer cells (Lv et al., 2021) or microorganisms (Xiao et al., 2023). PTT has shown particular potential for superficial tumors, such as skin cancer (Wang R. et al., 2023) and oral cancer (Wang Y. et al., 2024). Additionally, photothermal agents can target highly vascularized tumor tissues, demonstrating efficacy against solid tumors like breast, lung, liver, and kidney cancers (Li H. et al., 2023; Xiong et al., 2023; Yang et al., 2021; Yilmazer et al., 2023). Beyond cancer, PTT has shown promise in eradicating antibiotic-resistant bacteria (Yan et al., 2023). It is also being investigated for chronic inflammation-related diseases, such as arthritis, atherosclerosis, and Alzheimer’s disease, due to the regulatory effects of mild heat on immunity and inflammation (Liu et al., 2021; Yoo et al., 2021; Zhang M. et al., 2022). As a light-triggered therapeutic modality, PTT represents an important component of stimuli-responsive nanotherapies. Although photothermal heating alone primarily exerts its effects through thermal mechanisms, its integration with ROS- or radical-generating processes can further expand its therapeutic functions and enable synergistic treatment.

Therapeutic heating can be broadly classified into two categories according to the treatment temperature: hyperthermia and thermal ablation. Hyperthermia involves mild and transient elevation of tissue temperature, typically below 45 °C. Clinically, regional hyperthermia can enhance the efficacy of conventional therapies by improving tumor perfusion and oxygenation, impairing DNA repair, and modulating antitumor immune responses. In contrast, thermal ablation generally employs temperatures above 50 °C to directly damage tumor cells through processes such as protein denaturation and coagulation. Beyond direct thermal damage, moderate or localized heating can also alter the tumor microenvironment and facilitate other therapeutic processes, providing opportunities for the integration of PTT with complementary treatment modalities.

Metal-based nanomaterials are particularly attractive for stimuli-responsive photothermal therapy and photothermal-assisted combination therapy because their optical properties can be coupled with catalytic or radical-generating processes (Lv et al., 2021). When the excitation wavelength overlaps with the LSPR band of the nanoparticles, the incident light induces collective oscillation of conduction electrons, resulting in enhanced light–matter interactions. The LSPR response can be tuned by nanoparticle size, shape, composition, and the surrounding dielectric environment (Lee and Link, 2021). This process leads to efficient light absorption and rapid heat generation. The resulting optical extinction consists of both light absorption and scattering, whose relative contributions depend on nanoparticle size and morphology. For photothermal therapy, light absorption is particularly important because the absorbed optical energy is dissipated through non-radiative relaxation processes and ultimately converted into heat, whereas scattering contributes more substantially to optical imaging (Jain et al., 2006). Initially, the absorbed light energy excites conduction electrons, creating a non-equilibrium state. This energy is then redistributed through electron-electron collisions and transferred to the metal lattice via electron-phonon coupling, raising the temperature of the nanoparticle. The heat is subsequently dissipated into the surrounding medium, increasing the local temperature. AuNPs are among the most extensively investigated photothermal agents because of their tunable plasmonic absorption and efficient photothermal conversion. Additionally, the fluorescence properties of Au NPs enable real-time imaging, providing timely treatment feedback (Wang R. et al., 2022). Other noble metals, such as Ag (Zhao et al., 2023), platinum (Pt) (He et al., 2024) and palladium (Pd) (Zhang et al., 2023) also exhibit LSPR effects in the NIR region. However, their photothermal conversion efficiency and biocompatibility are generally inferior to those of Au, requiring further modifications and optimization. Non-noble metals, including Cu (Tai et al., 2018), nickel (Ni) (Song et al., 2023), Fe (Jiang et al., 2023), Ti (Wang Z. et al., 2024) have also been explored as photothermal agents in PTT. While their photothermal conversion efficiency is lower than that of noble metals, their lower cost and unique physicochemical properties expand the potential applications of PTT. For instance, Cu-based nanoparticles (∼50 ± 8 nm) have been shown to biodegrade and be excreted through the kidneys, reducing *in vivo* toxicity (Tai et al., 2018). Similarly, Fe_3_O_4_ has been incorporated into photosensitizers to enable multimodal imaging and therapy for subcutaneous tumors (Wang et al., 2021).

**FIGURE 3:**
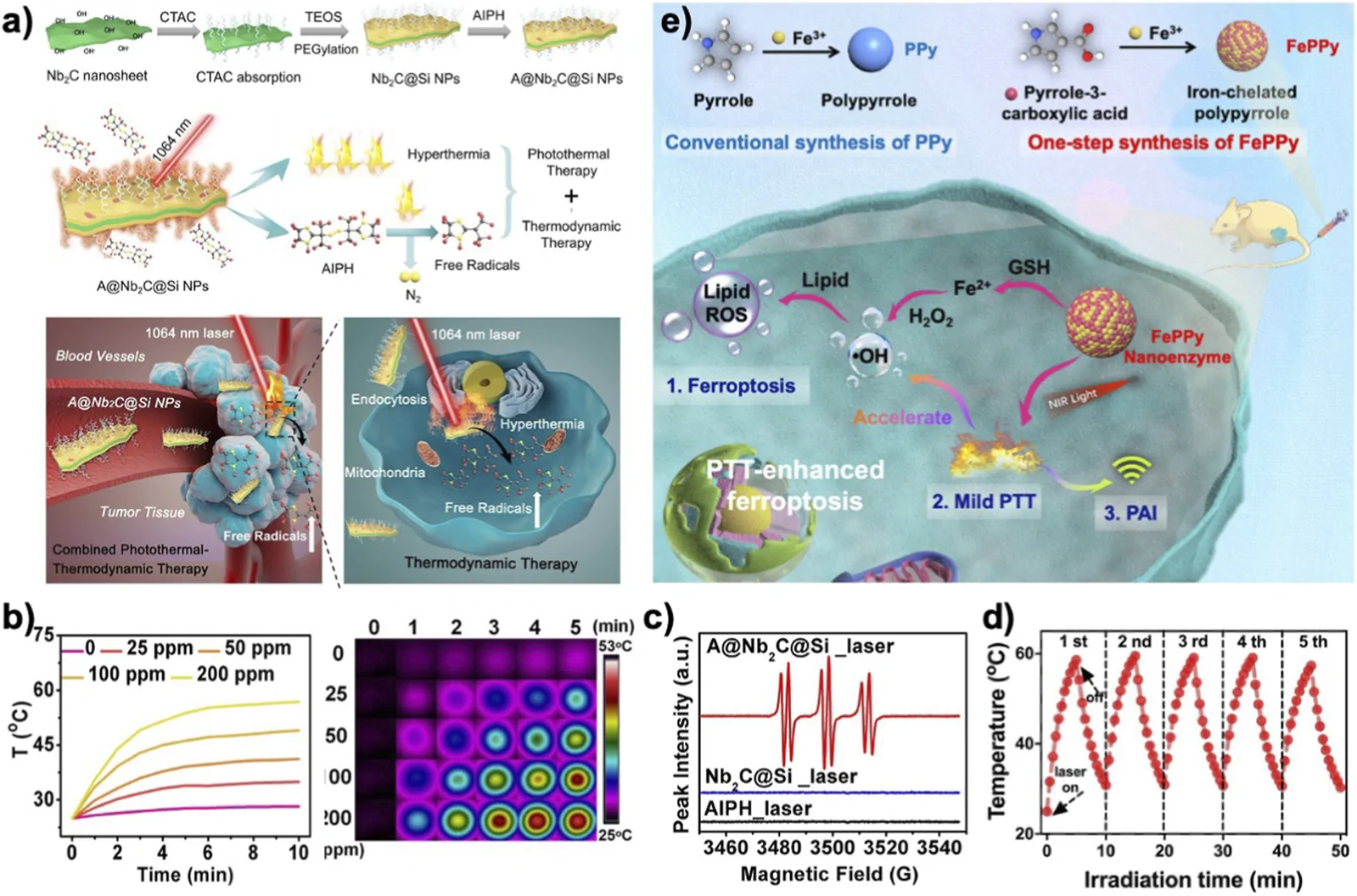
**(a)** Schematic illustration of the synthesis routes of silica-coated Nb_2_C MXene, and photothermal-induced AIPH decomposition for free radical production in antitumor treatment. **(b)** Photothermal curves of AIPH@Nb_2_C@mSiO_2_ NPs at different concentrations under 1,064 nm laser irradiation (1.0 W cm^–2^) and the corresponding infrared thermal images. **(c)** ESR spectra of different NPs solution under 1,064 nm laser irradiation for 3 min. Reproduced with permission (Xiang et al., 2019). Copyright 2019, American Chemical Society. **(d)** Temperature change profile of FePPy NPs under laser “on–off” cycles (1.0 W/cm^2^). **(e)** Schematic illustration of photoinduced therapies using FePPy nanoenzymes. Reproduced with permission (Cui et al., 2021). Copyright 2021, American Chemical Society.

A representative example of heat-triggered radical generation was reported by Xiang et al., who incorporated 2,2′-azobis [2-(2-imidazolin-2-yl)propane] dihydrochloride (AIPH) into silica-coated two-dimensional niobium carbide (Nb_2_C) MXene to construct AIPH@Nb_2_C@mSiO_2_ nanoparticles (Xiang et al., 2019). Upon 1,064 nm near-infrared irradiation, the Nb_2_C component efficiently converted light into heat, increasing the temperature of the nanosystem from 24.9 °C to 56.8 °C within 10 min (Figures 3a,b). Importantly, the therapeutic activity of this platform was not restricted to photothermal ablation. The localized temperature increase accelerated both the release and thermal decomposition of encapsulated AIPH, thereby generating cytotoxic free radicals (Figure 3C). Thus, photothermal conversion serves as an external trigger for radical generation, enabling effective tumor eradication *in vitro* and *in vivo*.

Photothermal heating can also enhance metal-catalyzed ROS generation by accelerating redox reactions. Lee et al., for example, developed iron-chelated polypyrrole (FePPy) nanoparticles composed of pyrrole-3-carboxylic acid and Fe^3+^ ions (Cui et al., 2021). The polypyrrole component acted as a photothermal converter, whereas the iron centers provided redox activity capable of promoting Fenton-mediated radical generation. Under 808 nm laser irradiation, the temperature of FePPy nanoparticles increased substantially (Figure 3d), thereby accelerating temperature-dependent Fenton chemistry. In parallel, Fe^3+^ interacted with intracellular glutathione (GSH), promoting its oxidation to glutathione disulfide and generating Fe^2+^ species that further supported Fenton reactions. The combined effects of GSH depletion, enhanced iron redox cycling, and photothermally accelerated radical generation increased oxidative stress and promoted ferroptotic cell death (Figure 3e; Cui et al., 2021). This system illustrates another mechanism of photothermal-assisted ROS-mediated therapy, in which heating does not directly generate radicals but instead accelerates catalytic reactions responsible for ROS production.

The dependence on external light nevertheless remains an important limitation of PTT-based nanotherapy platforms. NIR-I irradiation (approximately 700–900 nm) generally provides greater tissue penetration than visible light because of reduced absorption and scattering by major tissue components (Chu et al., 2022). However, its effective penetration remains highly dependent on wavelength, tissue type and composition, and irradiation conditions, which can restrict the treatment of deep-seated lesions. Moreover, some metal NPs such as silver NPs have relatively weak absorption in this region, limiting their photothermal conversion efficiency. The NIR-II window (approximately 1,000–1700 nm) generally enables deeper optical penetration than NIR-I because of reduced photon scattering and tissue autofluorescence (Chu et al., 2022; Li H. et al., 2023). However, the effective penetration of both NIR-I and NIR-II light is generally limited to the millimeter-to-centimeter scale depending on tissue type, wavelength, and irradiation conditions. Future strategies should focus not only on improving photothermal conversion efficiency but also on developing highly responsive systems and integrating photothermal stimulation with complementary deep-tissue activation modalities.

### 4.2 Photodynamic therapy

PDT is a clinically established light-activated therapeutic approach for cancer treatment based on photosensitizers and light. Similar to PTT, PDT employs specific light for activation and produces ROS to induce treatment. Many nanomaterials employed in PTT also serve as photosensitizers in PDT, making the two therapies often discussed together. Nanoparticles such as Au, Ag, Pd, CuS, and TiO_2_ can function as dual-modality PDT/PTT photosensitizers (An et al., 2023; Celikbas et al., 2023; Overchuk et al., 2023; Rodríguez-Barajas et al., 2022; Sheng et al., 2023).

However, unlike PTT, which relies on heat generation, PDT functions by producing ROS via two distinct pathways: Type I and Type II (Baptista et al., 2017). The Type I pathway involves direct electron or proton transfer between the photosensitizer and surrounding biomolecules, resulting in reactive intermediates such as superoxide anions, •OH, and H_2_O_2_. In contrast, the Type II pathway entails energy transfer from the excited triplet state of the photosensitizer to ground-state molecular oxygen (^3^O_2_), generating ^1^O_2_. ^1^O_2_ is a highly reactive species capable of oxidizing biomolecules and inducing cell or microbial damage (Baptista et al., 2017; Prejanò et al., 2023; Yu et al., 2022). These ROS, particularly singlet oxygen, are effective in destroying lesion tissues or microorganisms (Dobson et al., 2018). Similar to PTT, PDT has demonstrated efficacy in treating carcinomas (cutaneous tumors, basal cell carcinoma, equine sarcoids, prostate cancer, urinary bladder cancer, brain tumors, etc.), bacterial infections (Badran et al., 2023), and non-neoplastic conditions such as arthritis, skin diseases, and atherosclerosis (Gallardo-Villagrán et al., 2019; Kim et al., 2015; Lin et al., 2024).

PDT, like PTT, is most effective for lesions located closer to the surface. This limitation arises not only from the limited penetration depth of excitation light but also from the oxygen-dependent nature of ROS generation, particularly through the Type II pathway. Deep-seated lesions with poor blood supply are often hypoxic, significantly reducing PDT efficacy. To overcome this challenge, numerous studies explored the use of nanocarriers or nanoenzymes to enhance oxygen availability. For instance, Chen et al. developed porphyrin-lipoprotein nanoparticles capable of generating oxygen for PDT, effectively targeting deep glioblastomas (Chen et al., 2024). Another common strategy involves using nanoenzymes like manganese dioxide (MnO_2_) to catalyze the decomposition of H_2_O_2_ into oxygen, which can then be utilized by the photosensitizer (Chen Y. et al., 2021).

### 4.3 Sonodynamic therapy

Low-intensity US, as an exogenous physical trigger, possesses noninvasiveness and high tissue-penetrating capability, making it widely utilized in clinical diagnostic imaging and therapies (Yumita et al., 1989). Unlike PDT, in which photoexcitation directly activates photosensitizers to initiate ROS generation, SDT uses ultrasound as the primary external stimulus to activate sonosensitizers through several interconnected mechanisms. A major proposed pathway involves acoustic cavitation, in which ultrasound induces the oscillation and collapse of microbubbles, generating transient high-temperature and high-pressure conditions. The collapse of cavitation bubbles can produce sonoluminescence, resulting in the emission of short-lived photons. These photons can subsequently induce photoexcitation of sonosensitizers, generating excited states that undergo electron or energy transfer with surrounding molecules and oxygen, thereby producing ROS through mechanisms analogous to Type I and Type II PDT. Another proposed pathway involves cavitation-induced pyrolysis, in which the localized high temperatures generated during inertial cavitation promote the pyrolysis of water and other molecules, producing highly reactive species such as •OH and •H. These primary reactive species can subsequently react with oxygen and endogenous substrates, further contributing to ROS generation (Son et al., 2020). Thus, although both PDT and SDT ultimately rely on ROS-mediated cytotoxicity, PDT is directly initiated by light-driven photoexcitation, whereas SDT involves ultrasound-induced cavitation, with sonoluminescence-mediated photoexcitation and pyrolysis representing two distinct routes to reactive species generation.

On the other hand, SDT offers a distinct advantage over PDT due to the superior tissue-penetrating ability of ultrasound, making it particularly effective for targeting deep-seated lesions (Xu et al., 2023). Various metal-based nanomaterials have been investigated for SDT, including TiO_2_ (Li Y. et al., 2024), ZnO (Cai et al., 2024; Wang F. et al., 2022), Fe_3_O_4_ (Shen et al.,2015), noble metal (e.g., Au, Ag, and Pt) (Meng et al., 2022; Zhang et al., 2021), MnO_2_ (Xu et al., 2024), MoS_2_ (Chen H. et al., 2022) and CeO_2_ (Zheng et al., 2024). Notably, SDT is currently being investigated for the treatment of a range of medical conditions, including cancer, infections, inflammation, and neurodegenerative diseases (Li Y. et al., 2023; Wang M. et al., 2023; Zhu et al., 2024). It is also being explored for diagnostic imaging.

**FIGURE 4:**
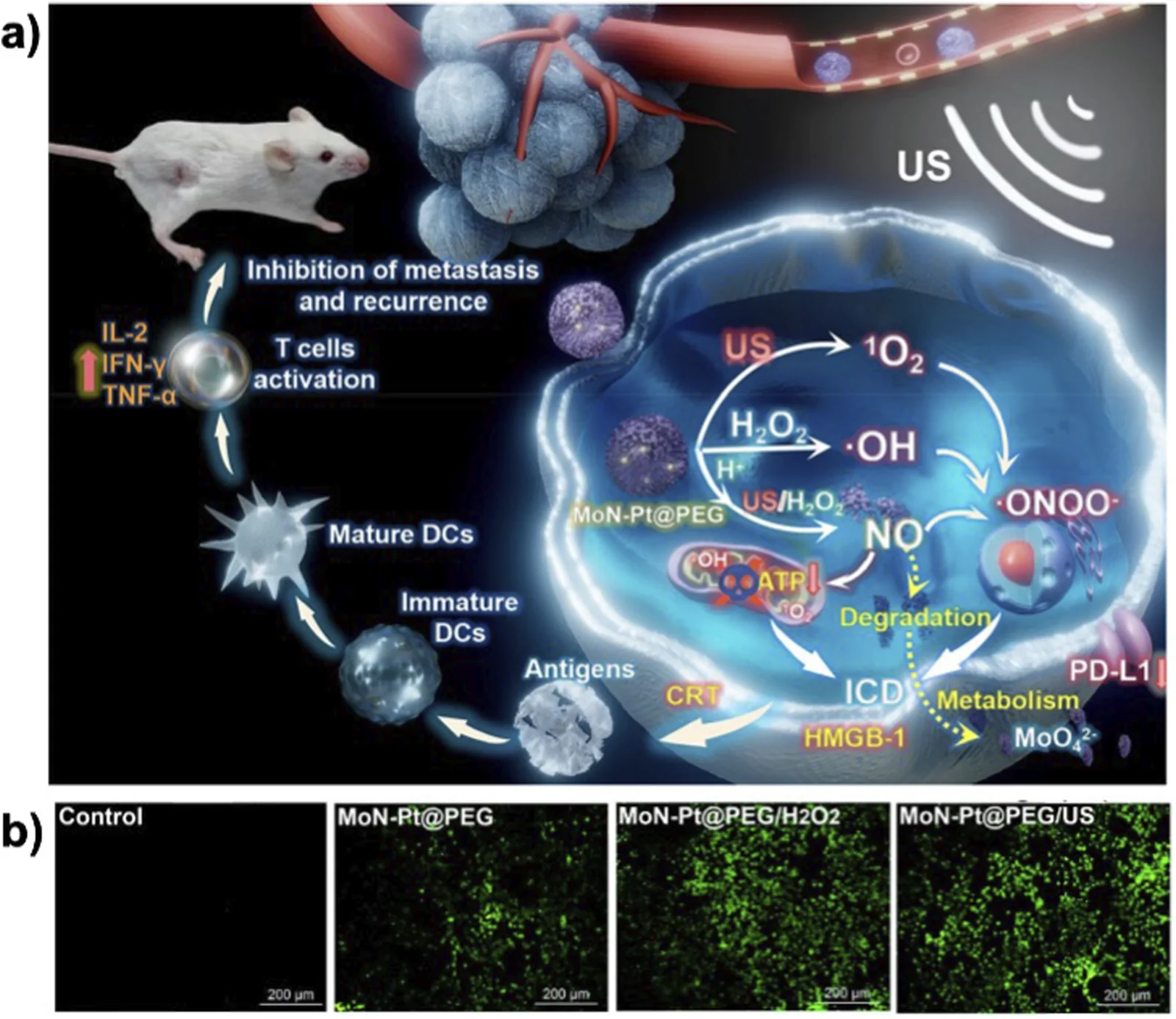
**(a)** Schematic diagram of synergetic SDT and NO gas therapy utilizing MoN-Pt@PEG for anticancer treatment and immune activation. **(b)** Fluorescence images of DCFH-DA stained 4T1 cells incubated with MoN-Pt@PEG under various conditions. Reproduced with permission (Bai et al., 2023). Copyright 2023, American Chemical Society.

The nanosonosensitizer-enabled SDT can effectively combine with other therapeutic approaches to achieve synergistic effects. This can be accomplished by carefully designing and engineering multifunctional nanosonosensitizers. For example, Bai et al. developed MoN-Pt@PEG NPs by encapsulating porous molybdenum nitride (MoN) nanospheres in PEG, as shown in Figure 4a (Bai et al., 2023). Upon ultrasound activation, the nanocomposites selectively released nitric oxide (NO) within the tumor microenvironment, promoting the formation of cytotoxic peroxynitrite (•ONOO^−^). Additionally, MoN-Pt@PEG NPs acted as sonosensitizers in SDT, triggering ROS production that damaged tumor cells (Bai et al., 2023). Under US irradiation, dissolved O_2_ can be transformed into ^1^O_2_, which possesses a higher oxidization ability (Figure 4b). This process facilitates the oxidation of MoN and the release of NO.

Despite significant advances in SDT, several challenges related to the control and reproducibility of acoustic cavitation remain. Cavitation is strongly influenced by acoustic parameters, including pressure amplitude and frequency, as well as by the physicochemical properties of the surrounding medium, such as dissolved gas content, viscosity, surface tension, temperature, and the presence of pre-existing cavitation nuclei (Wood et al., 2017). These factors determine bubble nucleation, growth, oscillation, and collapse, thereby affecting the spatial distribution of cavitation activity, sonoluminescence, localized energy deposition, and subsequent ROS generation. In particular, cavitation bubbles can form dense bubble clouds that scatter and attenuate the incident ultrasound field, potentially leading to heterogeneous energy deposition and reduced ultrasound transmission to regions located beyond the bubble cloud. Therefore, precise control of cavitation initiation and distribution remains an important consideration for achieving reproducible SDT effects. Dual-frequency sonication has been explored as one strategy to facilitate cavitation at lower acoustic pressures and enhance the efficiency of ultrasound-mediated therapeutic effects (Lafond et al., 2019). Alamolhoda et al. demonstrated that dual-frequency sonication using 150 kHz and 1 MHz ultrasound produced superior sonodynamic effects against breast adenocarcinoma in mice compared with either frequency alone (Alamolhoda et al., 2012). These findings suggest that combining different ultrasound frequencies may improve cavitation-mediated therapeutic efficacy, although the optimal frequency combination and acoustic parameters remain to be established.

Another limitation of SDT is the residual presence of sonosensitizers in normal tissues, which may lead to unintended cytotoxicity. Additionally, since many sensitizers used in both PDT and SDT overlap, patients may also experience increased sensitivity to light. To address these concerns, targeted drug delivery strategies have emerged as promising solutions. For instance, Wu et al. developed an exosome-packaged nanoplatform to deliver sonosensitizers specifically to glioblastoma, effectively overcoming the blood-brain barrier, an obstacle that often limits traditional therapies (Wu T. et al., 2022). Similarly, Abe et al. conjugated a sonosensitizer with F11-39, a monoclonal antibody with high affinity for carcinoembryonic antigen, to enable more precise and targeted SDT (Abe et al., 2002).

### 4.4 Piezocatalytic therapy

Piezocatalytic therapy (PCT) is a mechanically activated nanodynamic modality based on piezoelectric materials, which have the unique ability to convert mechanical forces into electrical fields (Chen et al., 2023). This process, driven by the piezoelectric effect, facilitates efficient interfacial charge transfer, contributing to the strong redox catalytic activities of piezoelectric materials. When subjected to mechanical stimulation, piezoelectric materials become polarized, resulting in material deformation due to the displacement of charge centers. This polarization distributes positive and negative charges on opposite surfaces of the material, enabling the catalysis of redox reactions. For instance, oxygen molecules can be reduced to generate superoxide anions (•O_2_^−^), and water molecules can be oxidized to produce •OH (Zhao et al., 2017). Additionally, You et al. reported that the piezoelectric materials they designed could continuously generate H_2_ by catalyzing H^+^ reduction through piezoelectric catalysis, significantly promoting the recovery of acute spinal cord injuries (You et al., 2024).

Nanomaterials used for PCT must possess piezoelectric properties. Common examples include BaTiO_3_ (Sappati and Bhadra, 2018), ZnO (Bhadwal et al., 2023), and SrTiO_3_ (Ling et al., 2020). Notably, since the material requires activation by external mechanical forces (typically compressive forces), it is often fabricated in the form of coatings or thin films to provide a larger contact area for force application (Wu et al., 2023).

Unlike PDT or SDT, ROS are not the sole active substances in PCT. The electronic signals produced by piezoelectric materials can also directly affect cells and biological processes. It is widely accepted that external electric stimulation can significantly regulate transmembrane potential and ion channels, influencing cellular behaviors such as stem cell differentiation, cell proliferation, cell migration, inflammatory cytokine secretion, and collagen production (Min et al., 2024). These unique features make piezoelectric materials and PCT widely applicable in tissue regeneration, beyond cancer or bacterial infection.

Externally applied electrical stimulation has demonstrated its potential to enhance bone fracture healing in animal and clinical experiments, as appropriate electric signals can mimic or enhance physiological signals for fracture healing (Min et al., 2024). For example, Wu et al. used scaffolds coated with piezoelectric materials such as BaTiO_3_ to enhance bone repair (Wu et al., 2023). Similarly, Li et al. designed a strontium-containing piezoelectric biofilm to promote the regeneration of dentin, another type of mineralized tissue (Li J. et al., 2024).

The activation factor of piezoelectric materials—mechanical force—is a very common form of stimulation. Therefore, many researchers have attempted to utilize mechanical forces generated during routine oral activities, such as tooth brushing and mastication, to activate piezocatalytic therapy (Su et al., 2025; Wang et al., 2020). This implies that PCT may operate independently of any specialized medical equipment or setting, allowing patients to receive treatment through everyday activities. Notably, the stimulation mechanisms targeting piezoelectric materials can originate not only from the physiological activity of the host but also from external physical stimulation in other forms, such as ultrasound. Sun et al. applied ultrasound with a frequency of 100 kHz and a power intensity of 0.7 W/cm^2^ to activate a nanogenerator, inducing diabetic bone regeneration (Sun et al., 2023). This approach enables dual-modal therapy combining PCT and SDT while allowing precise programming of the electrical signals released by the material to adapt to different stages of tissue healing.

### 4.5 Magnetic hyperthermia therapy

MHT is a promising therapeutic strategy based on magnetic nanoparticles. Magnetic NPs can generate heat when exposed to alternating magnetic field, affecting diseased tissues (Kafrouni and Savadogo, 2016). The heat generation mainly originates from magnetic relaxation processes, particularly Néel and Brownian relaxation. Néel relaxation involves the thermally induced reversal of the magnetic moment within an individual nanoparticle, whereas Brownian relaxation results from the physical rotation of the entire nanoparticle in response to the alternating magnetic field. These relaxation processes dissipate magnetic energy as heat (Kafrouni and Savadogo, 2016; Suriyanto et al., 2017). The relative contributions of Néel and Brownian relaxation depend on nanoparticle properties, including particle size, magnetic anisotropy, saturation magnetization, and hydrodynamic size, as well as the viscosity of the surrounding medium (Suriyanto et al., 2017; Nguyen et al., 2021). In general, smaller nanoparticles tend to exhibit more pronounced Néel relaxation, whereas Brownian relaxation becomes increasingly relevant for larger particles and depends strongly on their rotational mobility and the viscosity of the surrounding medium (Suriyanto et al., 2017; Nguyen et al., 2021). The most commonly used magnetic nanomaterials are iron oxide NPs due to its superparamagnetic properties and their ability to be metabolized into iron ions within the body (Kafrouni and Savadogo, 2016; Tran et al., 2012). Fe_3_O_4_ is the most common form.

Zero-valent iron (ZVI) nanoparticles represent another type of nanoparticle with excellent magnetic properties and the ability to rapidly accumulate in carcinoma tissue (Shevtsov et al., 2016). ZVI nanoparticles can induce strong oxidative stress by releasing iron ions and reducing the pH inside tumor cells, even in the absence of physical activators (Wu Y.-N. et al., 2022). However, ZVI has significant adverse effects on normal tissues. For instance, composite materials containing ZVI nanoparticles can exhibit notable neurotoxicity when their content reaches 20% (Aydemir Sezer et al., 2020). As a result, ZVI nanoparticles are currently primarily utilized in environmental and water treatment applications (Zhang S. et al., 2022), with their medical applications remaining limited. Future research is focused on improving their biocompatibility to expand their potential in the medical field.

While nickel and cobalt exhibit high magnetic moments, their susceptibility to oxidation and toxicity limits their clinical applications (Kafrouni and Savadogo, 2016). To address these challenges, researchers have developed cobalt ferrite (Medina et al., 2020) and cobalt zinc ferrite (Choppadandi et al., 2023), which demonstrate significantly improved biocompatibility.

Although both MHT and PTT rely on heat generation to treat lesions, MHT offers the unique advantage of targeting deep-seated lesions (Tansi et al., 2021). Additionally, magnetic nanoparticles such as Fe_3_O_4_ enable uniform heating under a magnetic field, minimizing the risks of local overheating or uneven temperature distribution (Du et al., 2019). Furthermore, functionalized magnetic nanoparticles can be guided by an external magnetic field, allowing for precise and targeted tumor treatment.

### 4.6 Chemodynamic therapy

Chemodynamic therapy is a novel cancer treatment technique based on Fenton or Fenton-like reactions. These reactions generate •OH through the catalytic decomposition of endogenous H_2_O_2_ in tumors using transition metals, primarily iron (Gao H. et al., 2023).

The reaction involves two key equations:

Main reaction:

$${\text{Fe}}^{2+}+H_{2}O_{2}\;\to \;{\text{Fe}}^{3+}+\cdot \text{OH}+{\text{OH}}^{-}$$

Side reaction:

$${\text{Fe}}^{3+}+H_{2}O_{2}\;\to \;{\text{Fe}}^{2+}+\cdot \text{OOH}+H^{+}$$

Through the side reaction, regenerated Fe^2+^ further contributes to the catalytic cycle.

Iron-based nanoparticles are widely used in CDT because they can be metabolized into iron ions within the body (Liu X. et al., 2020; Zhang L. et al., 2022). However, the Fenton reaction mediated by Fe-based NPs requires an acidic environment (pH 2.5–4) to maintain the solubility of iron ions, which is much lower than the typical pH of the tumor microenvironment. To address this limitation, alternative catalytic nanoparticles have been developed to extend the reaction conditions, including those based on Cu, Mn, Co, Mo, Sr, and Se (Gao H. et al., 2023; Wu et al., 2024). Among these alternatives, Cu-based systems have attracted particular attention because Cu^+^/Cu^2+^ can undergo Fenton-like redox cycling with H_2_O_2_ and maintain catalytic activity over a broader pH range than conventional Fe-based Fenton systems (Cao C. et al., 2022; Tang et al., 2019). In addition, Cu^2+^ can be reduced to Cu^+^ by intracellular reducing agents such as glutathione (GSH), while the consumption of GSH can further weaken the antioxidant defense of tumor cells and enhance oxidative stress (Liu G. et al., 2023; Tang et al., 2019). Mn-based systems have also been investigated as Fenton-like catalysts, with Mn^2+^ capable of generating ROS in the presence of H_2_O_2_ under appropriate reaction conditions (Cao C. et al., 2022; Gao H. et al., 2023). However, the catalytic activity of Mn-based systems is highly dependent on the Mn oxidation state and local reaction environment, and therefore does not necessarily eliminate the pH dependence of CDT (Gao H. et al., 2023).

MnO_2_ (Liu et al., 2023a) and Cu (Fu et al., 2021) nanoparticles stand out for their ability to react with intracellular GSH. By depleting GSH, these nanoparticles significantly enhance oxidative stress in tumors. Additionally, MnO_2_ nanoparticles release oxygen during their reactions, improving the hypoxic tumor microenvironment and providing substrates for ROS generation in multimodal therapy (Gao H. et al., 2023; Liu J. et al., 2023).

Cobalt nanoparticles (Co NPs) are another viable option for CDT. Gong et al. developed nanoparticles with a Co doping rate of 60% to regulate the tumor microenvironment (Gong et al., 2022). Thanks to the magnetic responsiveness of cobalt, Co-based CDT can leverage magnetic resonance imaging and magnetic field guidance to enhance treatment precision further (Gao Y. et al., 2023).

Another important limitation of CDT is the relatively limited availability of endogenous H_2_O_2_ in the tumor microenvironment. Although H_2_O_2_ levels are generally elevated in tumor cells compared with normal tissues, the endogenous concentration remains insufficient to sustain efficient Fenton/Fenton-like reactions. Reported intracellular H_2_O_2_ concentrations in cancer cells are typically in the micromolar range, and their levels can vary substantially among different tumor types and cellular compartments (Cao C. et al., 2022; Nie et al., 2022). Because H_2_O_2_ serves as the essential substrate for ·OH generation, insufficient H_2_O_2_ availability directly limits ·OH production and consequently compromises the therapeutic efficacy of CDT (Cao C. et al., 2022). Moreover, the rapid consumption of H_2_O_2_ during Fenton reactions further restricts the persistence of ROS generation, making endogenous H_2_O_2_ availability an important factor determining the catalytic efficiency and therapeutic outcome of CDT (Tang et al., 2019).

To overcome this limitation, considerable efforts have focused on increasing the local availability of H_2_O_2_ through self-supplying or *in situ* H_2_O_2_-generating nanoplatforms (Jana and Zhao, 2022). These strategies include the incorporation of H_2_O_2_-generating enzymes, nanozymes, and metal peroxides into CDT systems. For example, glucose oxidase (GOx)-based systems can catalyze the oxidation of glucose to generate H_2_O_2_, whereas metal peroxides such as CaO_2_ can react with water or protons in the tumor microenvironment to provide an additional source of H_2_O_2_ (Wang H. et al., 2025; Zhang J. et al., 2022).

In summary, CDT utilizes endogenous substrates for dynamic therapy without the need for external stimulation. While this reduces the controllability of treatment in terms of time and spatial precision, it remains highly suitable for specific clinical scenarios, such as deep tissue lesions or pH-responsive therapies.

### 4.7 Electrodynamic therapy

Electrodynamic therapy is an emerging therapeutic strategy that generates ROS through electric current or field stimulation. For instance, electric stimulation can activate platinum nanoparticles (Pt NPs) by inducing the Faraday cage effect on their surface, creating a hole-doping condition that triggers water molecule dissociation. In the presence of chloride ions, this process leads to the generation of hydroxyl radicals (Hu et al., 2021). Unlike other SRNs, EDT does not rely on specific substances like O_2_, H_2_O_2_, or H^+^, offering a unique advantage.

However, the use of electric stimulation, particularly direct electric current (DC), can produce harmful byproducts due to Faraday reactions. The oxidation of water generates hydrogen ions, leading to local acidification. Metal electrodes may release toxic metal ions, and chloride ions can undergo chlorination reactions, forming chlorine gas and corrosive hypochlorous acid. Additionally, the oxidation of amino acids and proteins can result in the production of harmful derivatives such as quinones and sulfides (Min et al., 2024).

**FIGURE 5:**
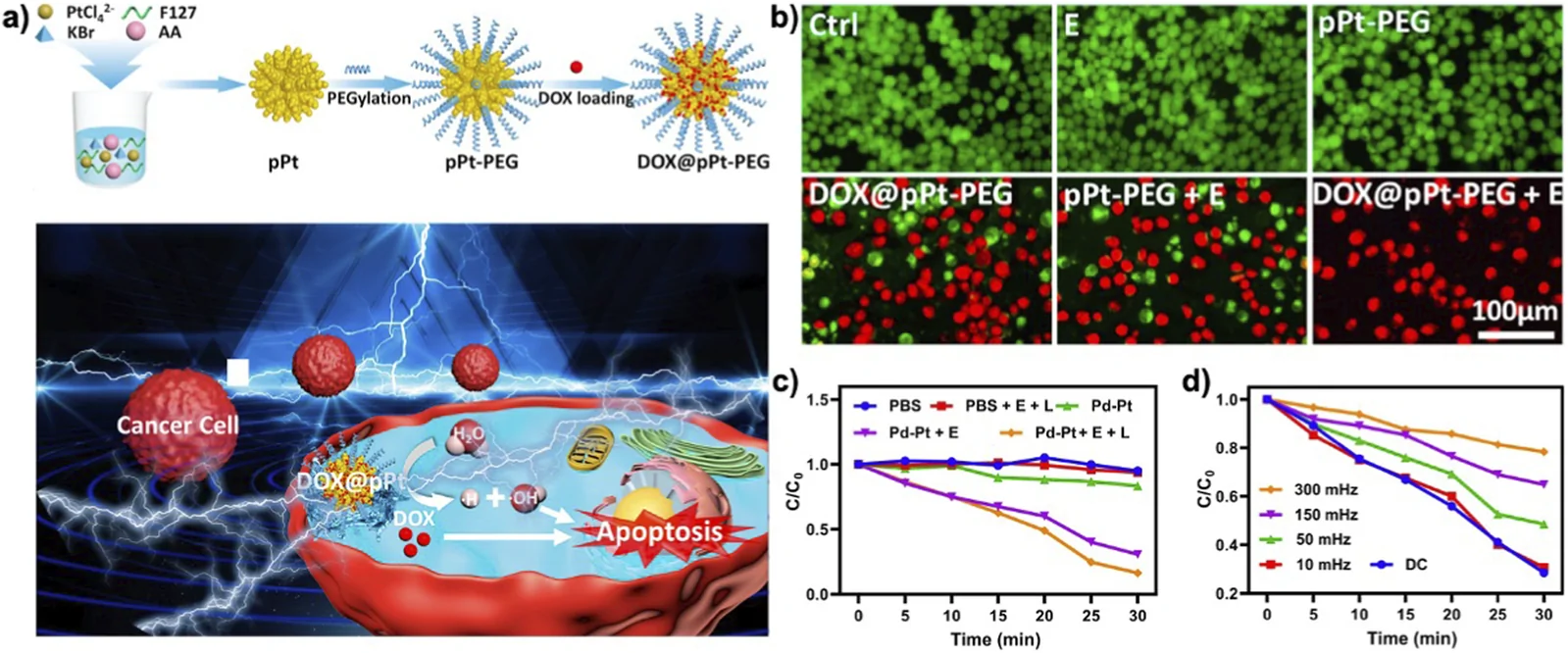
**(a)** Schematic diagram and synthesis of pPt-based drug delivery system for synergistic electrodynamic and chemotherapy in cancer treatment. **(b)** Fluorescence images of 4T1 cells co-stained with calcein-AM (green for live cells) and propidium iodide (red for dead cells) following different treatments. Reproduced with permission (Chen T. et al., 2020). Copyright 2020, Elsevier. **(c)** Degradation behaviors of MB under different treatments. **(d)** Degradation rates of MB under AC electric field with different frequencies or DC field in the presence of Pd-Pt nanosheets. Reproduced with permission (Qiao et al., 2022). Copyright 2022, Springer Nature.

While EDT is still in its early stages, the range of metal nanoparticles-based electrosensitizers (ES) is limited. Pt and Pt-based materials are the most extensively used due to their stability under electric current and fields (Gu et al., 2019). To date, the major types of Pt-based materials contain monometallic Pt electrosensitizers and Pt-containing composites. For instance, Chen et al. developed porous Pt NPs loaded with doxorubicin (DOX@pPt-PEG), which inhibited P-glycoprotein in breast cancer cells under square wave alternating current (AC) stimulation (5 mA), thereby reducing drug resistance (Figure 5a; Chen T. et al., 2020). According to Figure 5b, DOX@pPt-PEG NPs exhibited distinct cell damage under electric field. Similarly, Qiao et al. engineered extracellular vesicles (EV) Pd-Pt nanoparticles to enhance bacterial membrane penetration for infection control (Qiao et al., 2022). Obvious methylene blue (MB) degradation was observed in the presence of Pd-Pt nanosheets under square-wave AC electric field (E), suggesting the efficient generation of ·OH (Figure 5c). Additionally, as shown in Figure 5d, the NIR laser-induced heat could also enhance the generation efficiency of ·OH, paving the ways of synergistic electrodynamic therapy.

Most current EDT studies utilize AC stimulation. Unlike DC, AC stimulation alternates periodically between the anode and cathode, preventing the accumulation of harmful byproducts around the electrodes (Min et al., 2024). AC itself can also serve as a therapeutic modality by altering cellular membrane potentials, which regulates cellular behaviors. AC stimulation is already used in brain modulation, pain relief, and skin healing (Elyamany et al., 2021; Kambic et al., 1993; Zaghi et al., 2010). However, its relatively mild stimulation makes it less effective for applications such as bone healing or cancer treatment. Combining AC stimulation with nanomaterials has shown potential to achieve synergistic therapeutic effects beyond the individual contributions of either method alone.

In conclusion, EDT offers the potential advantage of being less dependent on specific components of the tissue microenvironment, such as oxygen, which may broaden its applicability under conditions where other dynamic therapies are less effective. Nevertheless, several challenges remain. Current EDT strategies commonly rely on electric fields generated between implanted electrodes, making treatment dependent on electrode placement and limiting accessibility to certain anatomical sites. Invasive electrode insertion and removal may also introduce procedural risks, including tissue injury and potential contamination, while the achievable insertion depth can restrict treatment of deeply located lesions (Chang et al., 2008; Chen F. et al., 2022; Llovet et al., 2001). To overcome these limitations, non-invasive electrical stimulation strategies, including radiofrequency-based approaches, capacitive coupling, inductive coupling, and externally applied electromagnetic fields, have been explored as potential alternatives. However, the interactions between such remotely delivered electrical stimuli and nanomaterials remain insufficiently understood. Clarifying how externally applied electric fields influence nanoparticle polarization, charge transfer, redox activity, and ROS generation will therefore be important for the future development of non-invasive EDT.

From a therapeutic-selection perspective, the major trade-off among these modalities lies between activation depth, spatiotemporal controllability, and dependence on the local microenvironment. Optical modalities provide precise external control but are constrained by tissue attenuation, whereas ultrasound and magnetic fields offer greater potential for deep-tissue activation. By contrast, CDT exploits endogenous chemical conditions without external energy delivery but sacrifices some temporal control and depends strongly on substrate availability. Future multimodal platforms should therefore be designed to combine complementary mechanisms rather than simply incorporating multiple therapeutic components.

## 5 Applications and future prospects

### 5.1 Tumor

Tumor treatment represents the primary application of SRNs. Extensive studies have elucidated the therapeutic effects and underlying mechanisms of PDT across various tumor types, highlighting key processes including oxidative stress induction, modulation of tumor immunity, promotion of apoptosis, and activation of autophagy (Donoh et al., 2019; Ji et al., 2022; Kubrak et al., 2022).

Tumor targeting is predominantly achieved by exploiting the leaky vasculature and impaired lymphatic drainage characteristic of tumor tissues. These features enable nanoscale particles to passively accumulate in solid tumors via the EPR effect (Fan et al., 2023). Upon accumulation, NPs can locally generate cytotoxic factors—such as ROS, thermal effects, or immune-activating signals—thereby inhibiting tumor growth and metastasis (Jelic et al., 2021).

However, the EPR effect exhibits substantial heterogeneity, varying across patients, tumor types, and even between primary and metastatic lesions within the same individual (Fan et al., 2023). This variability significantly limits the reliability of passive targeting. Moreover, in non-solid tumors such as hematological malignancies, the absence of well-defined tumor masses hinders localized nanoparticle accumulation, often resulting in off-target effects and damage to healthy tissues.

Therefore, improving delivery efficiency and enhancing targeting specificity remain critical challenges. One widely adopted strategy involves functionalizing nanoparticles with targeting ligands to achieve active targeting. For instance, tumor angiogenesis is highly dependent on the VEGF/VEGFR signaling pathway. Li et al. developed VEGFR2-targeted SiO_2_ nanoparticles and demonstrated effective tumor vasculature targeting in a 4T1 breast tumor model (Li et al., 2021). Similarly, Simelane and Abrahamse functionalized AuNPs with colorectal cancer-targeting monoclonal antibodies, enabling more precise PDT (Simelane and Abrahamse, 2024).

Beyond targeting bulk tumor cells, recent studies have increasingly focused on resistant cancer cell populations, particularly cancer stem cells (CSCs), which are associated with tumor recurrence, metastasis, and resistance to conventional chemotherapy and radiotherapy. Breast cancer stem cells (BCSCs), for example, exhibit characteristic markers such as CD44, CD326 (EpCAM), and ALDH, providing potential molecular targets for nanoparticle-mediated delivery (Gholizadeh and Yousefnia, 2026). Metal-based nanoparticles are particularly attractive in this context because their surfaces can be readily functionalized with ligands or biomolecules while their intrinsic optical, magnetic, or catalytic properties can be simultaneously exploited for therapeutic intervention. For example, hyaluronic acid-functionalized gold nanoplatforms have been developed to target CD44-overexpressing BCSCs, while combining targeted delivery with photothermal therapy or differentiation-inducing agents has been shown to suppress BCSC self-renewal and tumor growth (Pan et al., 2021; Xu et al., 2014). In addition, gold nanoparticle-based systems have been explored for delivering agents such as salinomycin or other therapeutic cargos to BCSCs, with the aim of overcoming their intrinsic therapeutic resistance (Chittineedi et al., 2023). These findings suggest that metal-based nanoplatforms may provide a means of integrating selective recognition of resistant tumor cell populations with localized or stimulus-responsive therapy.

Beyond cell-specific targeting, emerging strategies are increasingly moving toward molecular-level precision. In particular, RNA-loaded nanoparticle systems have shown promising potential in targeting cancer cells in hematological malignancies. This approach enables selective intervention at the genetic level, opening new avenues for the development of nanomaterials capable of recognizing mutation-specific cellular phenotypes (Garbayo et al., 2024). Collectively, these advances are driving SRNs toward a more personalized and precision-oriented paradigm.

### 5.2 Infections

SRNs combat infections through mechanisms including ROS generation and localized thermal damage, thereby reducing reliance on antibiotics and demonstrating efficacy against drug-resistant pathogens (Balderrama-González et al., 2021). Unlike conventional antimicrobial strategies, SRNs can be activated by external or endogenous stimuli, enabling localized or disease-responsive therapeutic effects while potentially reducing systemic toxicity and lowering the risk of resistance development. Beyond bacterial infections, emerging evidence also highlights their potential in antiviral applications. For instance, SRN has shown superior efficacy over conventional cryotherapy in the treatment of human papillomavirus (HPV)-associated flat warts, suggesting its broader applicability in infectious diseases (Shen et al., 2022).

Similar to tumor-targeted therapies, achieving high targeting specificity remains a central challenge in anti-infective SRNs. Infection sites are characterized by complex microenvironments, including inflammation, pH fluctuations, and the expression of specific virulence factors. Therefore, targeting these pathogenic determinants has emerged as an effective strategy. For example, Liu et al. developed a nanoparticle-enabled SDT platform capable of neutralizing vacuolating cytotoxin A, a key virulence factor secreted by *Helicobacter pylori*. This approach significantly reduced bacterial burden *in vivo* while preserving the integrity of the gut microbiota (Liu et al., 2024). These findings suggest that SRNs can extend beyond direct bactericidal effects to include virulence modulation, thereby enhancing therapeutic selectivity.

However, the presence of bacterial biofilms remains a major barrier to effective treatment. Biofilms form highly organized three-dimensional structures that not only hinder the penetration of nanomaterials and ROS but also promote bacterial tolerance by reducing metabolic activity. To overcome this limitation, recent efforts have focused on engineering biofilm-penetrating nanoplatforms. For instance, Habimana et al. functionalized AuNPs with proteinase K, enabling efficient degradation of the extracellular matrix in *Pseudomonas* fluorescens biofilms and significantly enhancing nanoparticle penetration (Habimana et al., 2018). In addition, surface charge engineering has been widely employed to improve biofilm targeting. Cationic nanoparticles can exploit electrostatic interactions with negatively charged bacterial membranes, leading to enhanced accumulation within biofilms. Yehiely et al. demonstrated that such cationic nanomaterials exhibit superior penetration and retention in *Pseudomonas aeruginosa* biofilms (Deiss-Yehiely et al., 2023). Furthermore, combining nanotherapy with ultrasound can mechanically disrupt biofilm structures, thereby amplifying therapeutic efficacy through synergistic effects (Yuwen et al., 2025).

In addition to structural barriers, hypoxia within infection sites represents another critical limitation for ROS-dependent therapies. Bacterial infections are often associated with significant oxygen depletion, which restricts ROS generation and compromises therapeutic outcomes (Mergani et al., 2023). To address this issue, the development of oxygen self-supplying nanoplatforms has gained increasing attention. For example, Su et al. synthesized MnO_2_-coated metal–organic framework (PCN-224) nanoparticles capable of *in situ* oxygen generation in infected bone defects, thereby enhancing antibacterial efficacy (Su et al., 2024). Similarly, Gu et al. engineered ZIF-8 nanoparticles co-functionalized with MnO_2_ and CaO_2_, enabling the simultaneous production of O_2_ and H_2_O_2_ to further boost ROS generation and therapeutic performance.

Collectively, current advances in anti-infective SRNs are shifting from direct pathogen eradication toward integrated strategies that combine microenvironment modulation, biofilm disruption, and multifunctional nanoplatform design. Future developments are expected to focus on the rational integration of targeting, penetration, and self-supplying capabilities to achieve more precise and efficient infection control.

### 5.3 Bone tissue regeneration

Metallic nanoparticles have emerged as bioactive regulators in bone tissue engineering, extending their role beyond passive scaffolding to actively modulate cellular behavior and the regenerative microenvironment. A growing body of evidence indicates that nanoparticles composed of gold, silver, iron, and aluminum can promote osteoblast differentiation and proliferation, suggesting that their physicochemical properties—such as surface reactivity, ion release, and nano–bio interface interactions—play a critical role in directing osteogenic processes (Eivazzadeh-Keihan et al., 2020).

The incorporation of nanoparticle-based SRNs further advances this paradigm by introducing spatiotemporally controllable biochemical cues. Unlike conventional biomaterials, these systems enable on-demand activation of signaling pathways through externally or internally triggered stimuli. For example, Yu et al. developed AuNP-based nanocarriers for miR-5106 delivery, achieving targeted regulation of gene expression in bone mesenchymal stromal cells and thereby enhancing osteogenic differentiation (Yu et al., 2017). This highlights a key advantage of dynamic nanoplatforms: the ability to couple gene regulation with nanoscale delivery for precise control of cell fate.

In parallel, AgNPs exemplify another functional dimension by integrating antibacterial activity with osteogenic support. Their capacity to release Ag^+^ ions enables effective infection control, which is critical in bone regeneration, particularly in contaminated or implant-associated defects. However, the therapeutic window of AgNPs is constrained by dose-dependent cytotoxicity. Strategies such as incorporating AgNPs into calcium apatite matrices not only mitigate toxicity but also create a synergistic microenvironment in which osteoconductivity and antimicrobial activity are co-optimized (Eivazzadeh-Keihan et al., 2020). This reflects an emerging design principle in bone engineering: balancing bioactivity and biosafety through compositional and structural integration.

Importantly, the therapeutic benefits of dynamic systems are not limited to nanomaterials themselves but also arise from the stimuli employed. Physical cues such as electric and magnetic fields are increasingly recognized as critical regulators of bone homeostasis, influencing ion channel activity, membrane potential, and downstream signaling pathways associated with osteogenesis. In this context, metallic implants can function as both structural supports and functional interfaces for energy transduction, enabling localized delivery of electrical stimulation directly to the defect site (Min et al., 2024). When combined with nanoparticle-based platforms, these systems establish a multiscale therapeutic framework in which mechanical, electrical, and biochemical signals are integrated to coordinate tissue regeneration.

Collectively, these advances indicate a conceptual shift in bone tissue engineering—from static material design toward dynamic, stimuli-responsive systems capable of actively orchestrating the regenerative microenvironment. Future strategies will likely focus on integrating programmable nanoplatforms with endogenous biological signals to achieve precise, adaptive, and patient-specific bone regeneration.

### 5.4 Neurological disorders

Metallic nanoparticles-based SRN has recently emerged as a promising strategy for the treatment of neurological disorders, with current research primarily focusing on brain tumors and neurodegenerative diseases (Cramer and Chen, 2019; Li et al., 2020). Compared with other disease systems, therapeutic intervention in the central nervous system presents unique challenges, including the presence of the blood–brain barrier (BBB), the complexity of neural networks, and the high sensitivity of neuronal cells to external perturbations. These constraints necessitate nanoplatforms that are not only capable of efficient delivery but also able to precisely modulate neural microenvironments.

Metallic nanoparticles offer distinct advantages in this context. First, their nanoscale dimensions and tunable surface properties enable them to cross the BBB, allowing them to function as carriers for therapeutic agents or as active nanotherapeutics. Second, their intrinsic physicochemical properties—such as conductivity, catalytic activity, and responsiveness to external stimuli—provide opportunities to directly interact with neuronal systems and regulate electrical signaling (Nasir et al., 2024). These dual capabilities position metallic nanoparticles as both delivery vehicles and functional modulators within the neural microenvironment.

Mechanistically, nanoparticle transport across the BBB can occur via multiple pathways, including hydrophobic paracellular pathway, lipophilic transcellular pathway, absorptive-mediated transcytosis, receptor-mediated transcytosis, and carrier-mediated transport (Quan et al., 2024). Importantly, these processes are not merely passive but can be influenced by nanoparticle design parameters such as size, morphology, and surface chemistry. For example, smaller nanoparticles may exhibit enhanced diffusion and cellular uptake, while surface properties can influence protein adsorption, receptor-mediated interactions, and the balance between BBB penetration and clearance (Asimakidou et al., 2024). Nanoparticle morphology can further affect cellular interactions and transport pathways across the BBB. This highlights a critical design principle: BBB permeability is an engineerable property, rather than a fixed biological barrier. Consequently, rational modulation of nano–bio interactions enables targeted delivery to deep-seated brain regions that are otherwise inaccessible to conventional therapeutics.

Beyond delivery, recent studies have demonstrated that metallic nanoparticles can actively modulate neuronal function. For example, magnetoelectric nanoparticles can convert external electromagnetic fields into localized electrical stimuli, enabling non-invasive regulation of neuronal activity at the cellular level (Zhang E. et al., 2024). This represents a shift from chemical-based interventions toward physical neuromodulation. In parallel, certain nanoparticles, such as TiO_2_ NPs, can generate ROS that influence neuroinflammatory pathways by activating microglia and neurons (Hsiao et al., 2016). While this effect may be therapeutically beneficial in controlled contexts, it also underscores the delicate balance between therapeutic activation and neurotoxicity in the central nervous system.

Indeed, the long-term fate of nanoparticles in neural tissues remains a critical concern. Due to the limited regenerative capacity of the central nervous system and restricted clearance pathways, the accumulation of non-degradable nanoparticles may lead to chronic inflammation or neurotoxicity. Therefore, the development of biodegradable and metabolizable nanomaterials has become a central focus in this field. Surface engineering strategies, including biomimetic coatings and responsive interfaces, are also being explored to enhance biocompatibility while preserving functional performance.

Collectively, these advances suggest that MNP-based SRNs for neurological disorders are evolving from a delivery-centric approach toward an integrated paradigm that combines BBB engineering, neuromodulation, and microenvironment regulation. Future developments will likely emphasize the design of adaptive, stimuli-responsive nanoplatforms capable of achieving precise and minimally invasive intervention within complex neural systems.

### 5.5 Cardiovascular therapy

Nanotherapies have recently emerged as a promising strategy for the treatment of cardiovascular diseases (CVDs), which remain the leading cause of mortality worldwide. Unlike tumor or infectious diseases, CVDs are characterized by complex vascular microenvironments involving oxidative stress, chronic inflammation, endothelial dysfunction, and abnormal hemodynamics. These features provide a unique biochemical and physical landscape that can be exploited by nanodynamic platforms for targeted and stimuli-responsive interventions (Flores et al., 2019).

Among various CVDs, atherosclerosis represents a primary target for SRNs. The progression of atherosclerotic plaques is closely associated with macrophage accumulation, lipid oxidation, and persistent inflammation. Nanoparticles can be engineered to selectively accumulate in plaques via endothelial dysfunction and enhanced vascular permeability, enabling localized therapeutic activation (Zhang Y. et al., 2024). For example, CuS/TiO_2_-based heterostructured nanosheets have been employed for sonodynamic/photothermal synergistic therapy, in which TiO_2_ primarily contributes to sonodynamic activity through efficient electron–hole separation, whereas CuS provides photothermal conversion through its strong NIR-II absorption. The resulting synergistic therapy induces apoptosis of proinflammatory macrophages, suppresses vascular inflammation, and inhibits atherosclerotic plaque progression (Cao Z. et al., 2022). This highlights a key advantage of SRNs in CVDs: the ability to modulate pathological cell populations within confined vascular lesions.

In the context of myocardial infarction, SRNs approach primarily aim to mitigate ischemia-induced oxidative damage and promote tissue repair. AuNPs, for instance, have demonstrated cardioprotective effects in ISO-induced myocardial injury models. This effect has been associated with their antioxidant activity, as evidenced by the restoration of endogenous antioxidant defenses, particularly GSH and SOD, which may help alleviate oxidative stress and subsequent myocardial injury (Younis et al., 2019). More broadly, nanoparticle-based delivery systems enable targeted transport of therapeutic agents to infarcted myocardium, improving drug bioavailability and therapeutic efficacy while minimizing systemic exposure (Jha et al., 2024). These findings underscore the potential of nanodynamic platforms to integrate antioxidative therapy, targeted delivery, and microenvironment modulation in cardiac repair.

Despite these advances, several critical challenges hinder clinical translation. The vascular system is highly sensitive to foreign materials, and issues such as cytotoxicity, immunogenicity, and long-term accumulation must be carefully addressed. In addition, the dynamic nature of blood flow introduces complexities in nanoparticle retention and targeting efficiency. From a translational perspective, scalable manufacturing and reproducibility also remain significant barriers (Yang et al., 2022). These challenges highlight the need for rational material design that balances functionality with biosafety, as well as a deeper understanding of nano–vascular interactions.

To overcome these limitations, current research is increasingly focused on biodegradable nanomaterials, biomimetic surface engineering, and stimuli-responsive systems that enable controlled activation within pathological sites. Moreover, strategies that enhance vascular targeting—such as shear stress-responsive delivery and endothelial-specific ligands—are gaining attention.

Looking forward, the future of SRNs in CVDs lies in the convergence of multifunctional nanoplatforms with precision medicine. The development of theranostic systems capable of simultaneous diagnosis and therapy offers a promising avenue for real-time monitoring and adaptive treatment. In addition, the integration of artificial intelligence and machine learning may enable prediction of patient-specific responses, thereby optimizing therapeutic strategies. Collectively, these advances are expected to transform SRNs from an experimental approach into a clinically viable modality for cardiovascular intervention.

## 6 Conclusions and perspectives

Metal nanoparticle-based SRNs have been extensively explored as versatile platforms for disease intervention. In this review, we have summarized recent advances in SRNs and highlighted multiple modalities—including photothermal, photodynamic, sonodynamic, piezocatalytic, chemodynamic, electrodynamic, and magnetic hyperthermia therapies—in diverse biomedical applications. Despite substantial progress at the experimental level, the clinical translation of SRNs remains limited by several fundamental and translational barriers.

From a translational perspective, one of the most critical challenges lies in the lack of clinically approved activation devices. Many SRN modalities rely on external stimuli that are currently confined to laboratory settings, and the absence of standardized, clinically adaptable equipment significantly restricts their practical implementation. In parallel, the mechanistic complexity of SRNs—particularly in multimodal systems—remains insufficiently understood. The interplay between different reactive species, physical stimuli, and biological responses introduces substantial uncertainty, hindering the rational design and optimization of therapeutic platforms.

Another major concern is the biosafety of nanosensitizers. Issues related to long-term biocompatibility, biodegradability, and *in vivo* clearance are particularly critical, especially for applications involving repeated administration or sensitive organs. Importantly, current research has been disproportionately focused on developing novel nanomaterials, whereas systematic evaluation of their biological effects and safety profiles remains relatively underexplored. Addressing this imbalance will be essential for advancing SRNs toward clinical use.

Looking forward, the future of SRNs is likely to be driven by three key directions: (i) the development of clinically compatible activation systems, (ii) deeper mechanistic understanding to enable rational multimodal design, and (iii) the creation of safe, biodegradable, and functionally optimized nanoplatforms. More broadly, SRN is evolving from a collection of individual therapeutic modalities into an integrated, microenvironment-adaptive platform capable of precise and controllable intervention.

With continued advances in nanomedicine, materials science, and biomedical engineering, SRN-based strategies are expected to transition from experimental research to clinical practice. The integration of multidisciplinary approaches, together with rigorous preclinical and clinical validation, will be essential to fully realize the potential of SRN in improving patient outcomes.

## References

[B1] Abe H. Kuroki M. Tachibana K. Li T. Awasthi A. Ueno A. (2002). Targeted sonodynamic therapy of cancer using a photosensitizer conjugated with antibody against carcinoembryonic antigen. Anticancer Res. 22 (3), 1575–1580. 12168839

[B2] Ahmad T. Sarwar R. Iqbal A. Bashir U. Farooq U. Halim S. A. (2020). Recent advances in combinatorial cancer therapy via multifunctionalized gold nanoparticles. Nanomedicine (Lond) 15 (12), 1221–1237. 10.2217/nnm-2020-0051 32370608

[B3] Alalaiwe A. Roberts G. Carpinone P. Munson J. Roberts S. (2017). Influence of PEG coating on the oral bioavailability of gold nanoparticles in rats. Drug Deliv. 24 (1), 591–598. 10.1080/10717544.2017.1282554 28222611 PMC8240969

[B4] Alamolhoda M. Mokhtari-Dizaji M. Barati A. H. Hasanzadeh H. (2012). Comparing the *in vivo* sonodynamic effects of dual- and single-frequency ultrasound in breast adenocarcinoma. J. Med. Ultrason. (2001) 39 (3), 115–125. 10.1007/s10396-012-0348-9 27278971

[B5] Alarifi S. Ali D. Al-Doaiss A. A. Ali B. A. Ahmed M. Al-Khedhairy A. A. (2013). Histologic and apoptotic changes induced by titanium dioxide nanoparticles in the livers of rats. Int. Journal Nanomedicine 8, 3937–3943. 10.2147/IJN.S47174 24143098 PMC3798149

[B6] Alexis F. Pridgen E. Molnar L. K. Farokhzad O. C. (2008). Factors affecting the clearance and biodistribution of polymeric nanoparticles. Mol. Pharm. 5 (4), 505–515. 10.1021/mp800051m 18672949 PMC2663893

[B7] Ali H. M. Karam K. Khan T. Wahab S. Ullah S. Sadiq M. (2023). Reactive oxygen species induced oxidative damage to DNA, lipids, and proteins of antibiotic-resistant bacteria by plant-based silver nanoparticles. 3 Biotech. 13 (12), 414. 10.1007/s13205-023-03835-1 38009163 PMC10665289

[B8] An Q. Su S. Hu W. Wang Y. Liang T. Li X. (2023). Dual-wavelength responsive CuS@COF nanosheets for high-performance photothermal/photodynamic combination treatments. Nanoscale 15 (48), 19815–19819. 10.1039/d3nr05219b 38051120

[B9] Asharani P. V. Lianwu Y. Gong Z. Valiyaveettil S. (2011). Comparison of the toxicity of silver, gold and platinum nanoparticles in developing zebrafish embryos. Nanotoxicology 5 (1), 43–54. 10.3109/17435390.2010.489207 21417687

[B10] Asimakidou E. Tan J. K. S. Zeng J. Lo C. H. (2024). Blood-brain barrier-targeting nanoparticles: biomaterial properties and biomedical applications in translational neuroscience. Pharm. (Basel) 17 (5), 612. 10.3390/ph17050612 PMC11123901 38794182

[B11] Atta S. Beetz M. Fabris L. (2019). Understanding the role of AgNO(3) concentration and seed morphology in the achievement of tunable shape control in gold nanostars. Nanoscale 11 (6), 2946–2958. 10.1039/c8nr07615d 30693922

[B12] Attar M. M. Amanpour S. Haghpanahi M. Haddadi M. Rezaei G. Muhammadnejad S. (2016). Thermal analysis of magnetic nanoparticle in alternating magnetic field on human HCT-116 Colon cancer cell line. Int. J. Hyperth. 32 (8), 858–867. 10.1080/02656736.2016.1204667 27418409

[B13] Aydemir Sezer U. Ozturk Yavuz K. Ors G. Bay S. Aru B. Sogut O. (2020). Zero-valent iron nanoparticles containing nanofiber scaffolds for nerve tissue engineering. J. Tissue Eng. Regen. Med. 14 (12), 1815–1826. 10.1002/term.3137 33010108

[B14] Aydın H. Er K. Kuştarcı A. Akarsu M. Gençer G. M. Er H. (2020). Antibacterial activity of silver nanoparticles activated by photodynamic therapy in infected root canals. Dent. Med. Probl. 57 (4), 393–400. 10.17219/dmp/123615 33444488

[B15] Azharuddin M. Zhu G. H. Das D. Ozgur E. Uzun L. Turner A. P. F. (2019). A repertoire of biomedical applications of noble metal nanoparticles. Chem. Commun. (Camb) 55 (49), 6964–6996. 10.1039/c9cc01741k 31140997

[B16] Azimzadeh M. Greco G. Farmani A. Pourhajibagher M. Taherkhani A. Alikhani M. Y. (2024). Synergistic effects of nano curcumin mediated photodynamic inactivation and nano-silver@colistin against *Pseudomonas aeruginosa* biofilms. Photodiagnosis Photodyn. Ther. 45, 103971. 10.1016/j.pdpdt.2024.103971 38218569

[B17] Badran Z. Rahman B. De Bonfils P. Nun P. Coeffard V. Verron E. (2023). Antibacterial nanophotosensitizers in photodynamic therapy: an update. Drug Discov. Today 28 (4), 103493. 10.1016/j.drudis.2023.103493 36657636

[B18] Bai X. Wang S. Yan X. Zhou H. Zhan J. Liu S. (2020). Regulation of cell uptake and cytotoxicity by nanoparticle core under the controlled shape, size, and surface chemistries. ACS Nano 14 (1), 289–302. 10.1021/acsnano.9b04407 31869202

[B19] Bai Q. Wang M. Liu J. Sun X. Yang P. Qu F. (2023). Porous molybdenum nitride nanosphere as carrier-free and efficient nitric oxide donor for synergistic nitric oxide and chemo/sonodynamic therapy. ACS Nano 17 (20), 20098–20111. 10.1021/acsnano.3c05790 37805936

[B20] Balderrama-González A. S. Piñón-Castillo H. A. Ramírez-Valdespino C. A. Landeros-Martínez L. L. Orrantia-Borunda E. Esparza-Ponce H. E. (2021). Antimicrobial resistance and inorganic nanoparticles. Int. J. Mol. Sci. 22 (23), 12890. 10.3390/ijms222312890 34884695 PMC8657868

[B21] Baptista M. S. Cadet J. Di Mascio P. Ghogare A. A. Greer A. Hamblin M. R. (2017). Type I and type II photosensitized Oxidation Reactions: guidelines and mechanistic pathways. Photochem Photobiol. 93 (4), 912–919. 10.1111/php.12716 28084040 PMC5500392

[B22] Bashiri G. Padilla M. S. Swingle K. L. Shepherd S. J. Mitchell M. J. Wang K. (2023). Nanoparticle protein corona: from structure and function to therapeutic targeting. Lab. Chip 23 (6), 1432–1466. 10.1039/d2lc00799a 36655824 PMC10013352

[B23] Basti H. Ben Tahar L. Smiri L. S. Herbst F. Vaulay M. J. Chau F. (2010). Catechol derivatives-coated Fe3O4 and γ-Fe2O3 nanoparticles as potential MRI contrast agents. J. Colloid Interface Sci. 341 (2), 248–254. 10.1016/j.jcis.2009.09.043 19853857

[B24] Bayda S. Adeel M. Tuccinardi T. Cordani M. Rizzolio F. (2019). The History of Nanoscience and Nanotechnology: From Chemical–Physical Applications to Nanomedicine. Molecules 2020 25 (1), 112. 10.3390/molecules25010112 31892180 PMC6982820

[B25] Bessa M. J. Costa C. Reinosa J. Pereira C. Fraga S. Fernández J. (2017). Moving into advanced nanomaterials. Toxicity of rutile TiO2 nanoparticles immobilized in nanokaolin nanocomposites on HepG2 cell line. Toxicol. Applied Pharmacology 316, 114–122. 10.1016/j.taap.2016.12.018 28039000

[B26] Bhadwal N. Ben Mrad R. Behdinan K. (2023). Review of zinc oxide piezoelectric nanogenerators: piezoelectric properties, composite structures and power output. Sensors (Basel) 23 (8), 3859. 10.3390/s23083859 37112200 PMC10144910

[B27] Bhuckory S. Lahtinen S. Höysniemi N. Guo J. Qiu X. Soukka T. (2023). Understanding FRET in upconversion nanoparticle nucleic acid biosensors. Nano Lett. 23 (6), 2253–2261. 10.1021/acs.nanolett.2c04899 36729707

[B28] Blanco E. Shen H. Ferrari M. (2015). Principles of nanoparticle design for overcoming biological barriers to drug delivery. Nat. Biotechnol. 33 (9), 941–951. 10.1038/nbt.3330 26348965 PMC4978509

[B29] Blanco-Andujar C. Ortega D. Southern P. Pankhurst Q. A. Thanh N. T. K. (2015). High performance multi-core iron oxide nanoparticles for magnetic hyperthermia: microwave synthesis, and the role of core-to-core interactions. Nanoscale 7 (5), 1768–1775. 10.1039/c4nr06239f 25515238

[B30] Brinson B. E. Lassiter J. B. Levin C. S. Bardhan R. Mirin N. Halas N. J. (2008). Nanoshells made easy: improving Au layer growth on nanoparticle surfaces. Langmuir 24 (24), 14166–14171. 10.1021/la802049p 19360963 PMC5922771

[B31] Cai X. Luo Y. Zhang W. Du D. Lin Y. (2016). pH-Sensitive ZnO quantum dots-doxorubicin nanoparticles for lung cancer targeted drug delivery. ACS Appl. Mater Interfaces 8 (34), 22442–22450. 10.1021/acsami.6b04933 27463610

[B32] Cai L. Sun T. Han F. Zhang H. Zhao J. Hu Q. (2024). Degradable and piezoelectric hollow ZnO heterostructures for sonodynamic therapy and pro-death autophagy. J. Am. Chem. Soc. 146 (49), 34188–34198. 10.1021/jacs.4c14489 39582172

[B33] Cao C. Wang X. Yang N. Song X. Dong X. (2022a). Recent advances of cancer chemodynamic therapy based on Fenton/Fenton-like chemistry. Chem. Sci. 13 (4), 863–889. 10.1039/d1sc05482a PMC8790788 35211255

[B34] Cao Z. Yuan G. Zeng L. Bai L. Liu X. Wu M. (2022b). Macrophage-Targeted Sonodynamic/Photothermal synergistic therapy for preventing atherosclerotic plaque progression using CuS/TiO(2) heterostructured nanosheets. ACS Nano 16 (7), 10608–10622. 10.1021/acsnano.2c02177 35759554

[B35] Caro C. Gámez F. Quaresma P. Páez-Muñoz J. M. Domínguez A. Pearson J. R. (2021). Fe3O4-Au core-shell nanoparticles as a multimodal platform for *in vivo* imaging and focused photothermal therapy. Pharmaceutics 13, 416. 10.3390/pharmaceutics13030416 PMC8003746 33804636

[B36] Celikbas E. Saymaz A. Gunduz H. Koc I. Cakir E. Sennaroglu A. (2023). Image-Guided enhanced PDT/PTT combination therapy using brominated hemicyanine-loaded folate receptor-targeting Ag(2)S Quantum dots. Bioconjug Chem. 34 (5), 880–892. 10.1021/acs.bioconjchem.3c00096 37078275 PMC10197082

[B37] Chang S. Kim S. H. Lim H. K. Kim S. H. Lee W. J. Choi D. (2008). Needle tract implantation after percutaneous interventional procedures in hepatocellular carcinomas: lessons learned from a 10-year experience. Korean J. Radiol. 9 (3), 268–274. 10.3348/kjr.2008.9.3.268 18525230 PMC2627263

[B38] Chen J. Saeki F. Wiley B. J. Cang H. Cobb M. J. Li Z. Y. (2005). Gold nanocages: bioconjugation and their potential use as optical imaging contrast agents. Nano Lett. 5 (3), 473–477. 10.1021/nl047950t 15755097

[B39] Chen L. Q. Fang L. Ling J. Ding C. Z. Kang B. Huang C. Z. (2015). Nanotoxicity of silver nanoparticles to red blood cells: size dependent adsorption, uptake, and hemolytic activity. Chem. Res. Toxicol. 28 (3), 501–509. 10.1021/tx500479m 25602487

[B40] Chen C.-W. Chan Y. C. Hsiao M. Liu R. S. (2016). Plasmon-Enhanced Photodynamic Cancer Therapy by Upconversion Nanoparticles Conjugated with Au Nanorods. ACS Appl. Mater. and Interfaces 8 (47), 32108–32119. 10.1021/acsami.6b07770 27933825

[B41] Chen T. Gu T. Cheng L. Li X. Han G. Liu Z. (2020a). Porous Pt nanoparticles loaded with doxorubicin to enable synergistic Chemo-/Electrodynamic Therapy. Biomaterials 255, 120202. 10.1016/j.biomaterials.2020.120202 32562941

[B42] Chen D. Ganesh S. Wang W. Amiji M. (2020b). Protein corona-enabled systemic delivery and targeting of nanoparticles. Aaps J. 22 (4), 83. 10.1208/s12248-020-00464-x 32495039

[B43] Chen B. M. Cheng T. L. Roffler S. R. (2021a). Polyethylene glycol immunogenicity: theoretical, clinical, and practical aspects of anti-polyethylene glycol antibodies. ACS Nano 15 (9), 14022–14048. 10.1021/acsnano.1c05922 34469112

[B44] Chen Y. Cai Y. Yu X. Xiao H. He H. Xiao Z. (2021b). A photo and tumor microenvironment activated nano-enzyme with enhanced ROS generation and hypoxia relief for efficient cancer therapy. J. Mater Chem. B 9 (39), 8253–8262. 10.1039/d1tb01437d 34515282

[B45] Chen T. Chu Q. Li M. Han G. Li X. (2021c). Fe(3)O(4)@Pt nanoparticles to enable combinational electrodynamic/chemodynamic therapy. J. Nanobiotechnology 19 (1), 206. 10.1186/s12951-021-00957-7 34246260 PMC8272323

[B46] Chen Y. Sheng W. Lin J. Fang C. Deng J. Zhang P. (2022a). Magnesium oxide nanoparticle coordinated phosphate-functionalized chitosan injectable hydrogel for osteogenesis and angiogenesis in bone regeneration. ACS Appl. Mater Interfaces 14 (6), 7592–7608. 10.1021/acsami.1c21260 35119809

[B47] Chen H. He X. Zhou Z. Wu Z. Li H. Peng X. (2022b). Metallic phase enabling MoS(2) nanosheets as an efficient sonosensitizer for photothermal-enhanced sonodynamic antibacterial therapy. J. Nanobiotechnology 20 (1), 136. 10.1186/s12951-022-01344-6 PMC8922895 35292034

[B48] Chen F. Meng X. Li T. Xu Z. Li S. Zhou Y. (2022c). Predictive nomogram for deep brain stimulation-related infections. Neurosurg. Focus 53 (6), E8. 10.3171/2022.9.focus21558 36455280

[B49] Chen S. Zhu P. Mao L. Wu W. Lin H. Xu D. (2023). Piezocatalytic medicine: an emerging frontier using piezoelectric materials for biomedical applications. Adv. Mater. 35 (25), 2208256. 10.1002/adma.202208256 36634150

[B50] Chen Y. Ma Y. Shi K. Chen H. Han X. Wei C. (2024). Self-Disassembling and oxygen-generating porphyrin-lipoprotein nanoparticle for targeted glioblastoma resection and enhanced photodynamic therapy. Adv. Mater 36 (15), e2307454. 10.1002/adma.202307454 38299428

[B51] Cheng Y. Tao J. Zhang Y. Xi L. Han R. Xu M. (2023a). Shape and shear stress impact on the toxicity of mesoporous Silica nanoparticles: *in vitro* and *in vivo* evidence. Mol. Pharm. 20 (6), 3187–3201. 10.1021/acs.molpharmaceut.3c00180 37167021

[B52] Cheng S. Qi M. Li W. Sun W. Li M. Lin J. (2023b). Dual-Responsive nanocomposites for synergistic antibacterial therapies facilitating bacteria-infected wound healing. Adv. Healthc. Mater 12 (6), e2202652. 10.1002/adhm.202202652 36373219

[B53] Chittineedi P. Mohammed A. Abdul Razab M. K. A. Mat Nawi N. Pandrangi S. L. (2023). Polyherbal formulation conjugated to gold nanoparticles induced ferroptosis in drug-resistant breast cancer stem cells through ferritin degradation. Front. Pharmacol. 14, 1134758. 10.3389/fphar.2023.1134758 37050902 PMC10083297

[B54] Choi S. J. Choy J. H. (2014). Biokinetics of zinc oxide nanoparticles: toxicokinetics, biological fates, and protein interaction. Int. J. Nanomedicine 9 (Suppl. 2), 261–269. 10.2147/IJN.S57920 25565844 PMC4279768

[B55] Choi H. S. Liu W. Misra P. Tanaka E. Zimmer J. P. Itty Ipe B. (2007). Renal clearance of quantum dots. Nat. Biotechnol. 25 (10), 1165–1170. 10.1038/nbt1340 17891134 PMC2702539

[B56] Choi C. H. Zuckerman J. E. Webster P. Davis M. E. (2011). Targeting kidney mesangium by nanoparticles of defined size. Proc. Natl. Acad. Sci. U. S. A. 108 (16), 6656–6661. 10.1073/pnas.1103573108 21464325 PMC3080986

[B57] Choppadandi M. Parmar K. Rao K. S. Rao K. H. Singh A. Kumar H. (2023). Self-regulated cobalt zinc ferrite system as a potential nanoplatform for the synergistic effect of hyperthermia-chemo agent for cancer therapy. Colloids Surf. B Biointerfaces 222, 113077. 10.1016/j.colsurfb.2022.113077 36577341

[B58] Chu Y. Xu X.-Q. Wang Y. (2022). Ultradeep photothermal therapy strategies. J. Phys. Chem. Lett. 13 (41), 9564–9572. 10.1021/acs.jpclett.2c02642 36201623

[B59] Colombeau L. Acherar S. Baros F. Arnoux P. Gazzali A. M. Zaghdoudi K. (2016). Inorganic nanoparticles for photodynamic therapy. Top. Curr. Chem. 370, 113–134. 10.1007/978-3-319-22942-3_4 26589507

[B60] Cramer S. W. Chen C. C. (2019). Photodynamic therapy for the treatment of glioblastoma. Front. Surg. 6, 81. 10.3389/fsurg.2019.00081 32039232 PMC6985206

[B61] Creixell M. Bohórquez A. C. Torres-Lugo M. Rinaldi C. (2011). EGFR-targeted magnetic nanoparticle heaters kill cancer cells without a perceptible temperature rise. ACS Nano 5 (9), 7124–7129. 10.1021/nn201822b 21838221

[B62] Cui X. Lu G. Fang F. Xiong Y. Tian S. Wan Y. (2021). Iron self-boosting polymer nanoenzyme for low-temperature photothermal-enhanced ferrotherapy. ACS Appl. Mater Interfaces 13 (26), 30274–30283. 10.1021/acsami.1c01658 34170100

[B63] Dai C. Zhang S. Liu Z. Wu R. Chen Y. (2017). Two-Dimensional graphene augments nanosonosensitized sonocatalytic tumor eradication. ACS Nano 11 (9), 9467–9480. 10.1021/acsnano.7b05215 28829584

[B64] Dan M. Bae Y. Pittman T. A. Yokel R. A. (2015). Alternating magnetic field-induced hyperthermia increases iron oxide nanoparticle cell association/uptake and flux in blood-brain barrier models. Pharm. Res. 32 (5), 1615–1625. 10.1007/s11095-014-1561-6 25377069 PMC4803069

[B65] Deiss-Yehiely E. Cárcamo-Oyarce G. Berger A. G. Ribbeck K. Hammond P. T. (2023). pH-Responsive, charge-reversing layer-by-layer nanoparticle surfaces enhance biofilm penetration and eradication. ACS Biomater. Sci. Eng. 9 (8), 4794–4804. 10.1021/acsbiomaterials.3c00481 37390118 PMC11117027

[B66] Deng C. Zheng M. Han S. Wang Y. Xin J. Aras O. (2023). GSH-activated porphyrin sonosensitizer prodrug for Fluorescence imaging-guided cancer sonodynamic therapy. Adv. Funct. Mater 33 (32), 2300348. 10.1002/adfm.202300348 38045635 PMC10691834

[B67] Dhanalekshmi K. I. Magesan P. Sangeetha K. Zhang X. Jayamoorthy K. Srinivasan N. (2019). Preparation and characterization of core-shell type Ag@SiO(2) nanoparticles for photodynamic cancer therapy. Photodiagnosis Photodyn. Ther. 28, 324–329. 10.1016/j.pdpdt.2019.10.006 31600577

[B68] Dirersa W. B. Kan T.-C. Getachew G. Wibrianto A. Ochirbat O. Rasal A. (2023). Preclinical assessment of enhanced chemodynamic therapy by an FeMnO(x)-Based nanocarrier: Tumor-Microenvironment-Mediated fenton reaction and ROS-Induced chemotherapeutic for boosted antitumor activity. ACS Appl. Mater Interfaces.15 (48), 55258–55275. 10.1021/acsami.3c10733 38013418

[B69] Dobson J. de Queiroz G. F. Golding J. P. (2018). Photodynamic therapy and diagnosis: principles and comparative aspects. Veterinary J. 233, 8–18. 10.1016/j.tvjl.2017.11.012 29486883

[B70] Donohoe C. Senge M. O. Arnaut L. G. Gomes-da-Silva L. C. (2019). Cell death in photodynamic therapy: from oxidative stress to anti-tumor immunity. Biochim. Biophys. Acta Rev. Cancer 1872 (2), 188308. 10.1016/j.bbcan.2019.07.003 31401103

[B71] Dorier M. Brun E. Veronesi G. Barreau F. Pernet-Gallay K. Desvergne C. (2015). Impact of anatase and rutile titanium dioxide nanoparticles on uptake carriers and efflux pumps in Caco-2 gut epithelial cells. Nanoscale 7 (16), 7352–7360. 10.1039/c5nr00505a 25825056

[B72] Du Y. Liu X. Liang Q. Liang X. J. Tian J. (2019). Optimization and design of magnetic ferrite nanoparticles with uniform tumor distribution for highly sensitive MRI/MPI performance and improved magnetic hyperthermia therapy. Nano Lett. 19 (6), 3618–3626. 10.1021/acs.nanolett.9b00630 31074627

[B73] Eivazzadeh-Keihan R. Bahojb Noruzi E. Khanmohammadi Chenab K. Jafari A. Radinekiyan F. Hashemi S. M. (2020). Metal-based nanoparticles for bone tissue engineering. J. Tissue Eng. Regen. Med. 14 (12), 1687–1714. 10.1002/term.3131 32914573

[B74] El Badawy A. M. Silva R. G. Morris B. Scheckel K. G. Suidan M. T. Tolaymat T. M. (2011). Surface charge-dependent toxicity of silver nanoparticles. Environ. Sci. Technol. 45 (1), 283–287. 10.1021/es1034188 21133412

[B75] Elyamany O. Leicht G. Herrmann C. S. Mulert C. (2021). Transcranial alternating current stimulation (tACS): from basic mechanisms towards first applications in psychiatry. Eur. Arch. Psychiatry Clin. Neurosci. 271 (1), 135–156. 10.1007/s00406-020-01209-9 33211157 PMC7867505

[B76] Fan D. Cao Y. Cao M. Wang Y. Cao Y. Gong T. (2023). Nanomedicine in cancer therapy. Signal Transduct. Target Ther. 8 (1), 293. 10.1038/s41392-023-01536-y 37544972 PMC10404590

[B77] Fang B. Y. Li C. An J. Zhao S. D. Zhuang Z. Y. Zhao Y. D. (2018). HIV-related DNA detection through switching on hybridized quenched fluorescent DNA-Ag nanoclusters. Nanoscale 10 (12), 5532–5538. 10.1039/c7nr09647j 29513333

[B78] Flores A. M. Ye J. Jarr K. U. Hosseini-Nassab N. Smith B. R. Leeper N. J. (2019). Nanoparticle therapy for vascular diseases. Arterioscler. Thromb. Vasc. Biol. 39 (4), 635–646. 10.1161/ATVBAHA.118.311569 30786744 PMC6436996

[B79] Foroozandeh P. Aziz A. A. (2018). Insight into cellular uptake and intracellular trafficking of nanoparticles. Nanoscale Res. Lett. 13 (1), 339. 10.1186/s11671-018-2728-6 30361809 PMC6202307

[B80] Frenzilli G. Bernardeschi M. Guidi P. Scarcelli V. Lucchesi P. Marsili L. (2014). Effects of *in vitro* exposure to titanium dioxide on DNA integrity of bottlenose dolphin (Tursiops truncatus) fibroblasts and leukocytes. Mar. Environmental Research 100, 68–73. 10.1016/j.marenvres.2014.01.002 24484603

[B81] Fu L.-H. Wan Y. Qi C. He J. Li C. Yang C. (2021). Nanocatalytic theranostics with glutathione depletion and enhanced reactive oxygen species generation for efficient cancer therapy. Adv. Mater. 33 (7), 2006892. 10.1002/adma.202006892 33394515

[B82] Gallardo-Villagrán M. Leger D. Y. Liagre B. Therrien B. (2019). Photosensitizers used in the photodynamic therapy of rheumatoid arthritis. Int. J. Mol. Sci. 20 (13), 3339. 10.3390/ijms20133339 31284664 PMC6651633

[B83] Gao F. He G. Yin H. Chen J. Liu Y. Lan C. (2019). Titania-coated 2D gold nanoplates as nanoagents for synergistic photothermal/sonodynamic therapy in the second near-infrared window. Nanoscale 11 (5), 2374–2384. 10.1039/c8nr07188h 30667014

[B84] Gao H. Cao Z. Liu H. Chen L. Bai Y. Wu Q. (2023a). Multifunctional nanomedicines-enabled chemodynamic-synergized multimodal tumor therapy via Fenton and Fenton-like reactions. Theranostics 13 (6), 1974–2014. 10.7150/thno.80887 PMC10091877 37064867

[B85] Gao Y. Ouyang Z. Shen S. Yu H. Jia B. Wang H. (2023b). Manganese dioxide-entrapping dendrimers Co-Deliver protein and nucleotide for magnetic resonance imaging-guided chemodynamic/starvation/Immune therapy of tumors. ACS Nano 17 (23), 23889–23902. 10.1021/acsnano.3c08174 38006397

[B86] Garbayo E. El Moukhtari S. H. Rodríguez-Nogales C. Agirre X. Rodriguez-Madoz J. R. Rodriguez-Marquez P. (2024). RNA-loaded nanoparticles for the treatment of hematological cancers. Adv. Drug Deliv. Rev. 214, 115448. 10.1016/j.addr.2024.115448 39303823

[B87] Gholizadeh Z. Yousefnia S. (2026). Application of metal-based nanoparticles in targeted drug delivery to breast cancer stem cells. Discov. Nano 21 (1), 318. 10.1186/s11671-026-04772-7 42400744 PMC13332929

[B88] Golbek T. W. Harper B. J. Harper S. L. Baio J. E. (2022). Shape-dependent gold nanoparticle interactions with a model cell membrane. Biointerphases 17 (6), 061003. 10.1116/6.0002183 36347646 PMC9646251

[B89] Gong Y. Leng J. Guo Z. Ji P. Qi X. Meng Y. (2022). Cobalt doped in Zn-MOF-5 nanoparticles to regulate tumor microenvironment for tumor Chemo/Chemodynamic therapy. Chem. Asian J. 17 (16), e202200392. 10.1002/asia.202200392 35621703

[B90] Groysbeck N. Hanss V. Donzeau M. Strub J. Cianférani S. Spehner D. (2023). Bioactivated and PEG-Protected circa 2 nm gold nanoparticles for in cell labelling and cryo-electron microscopy. Small Methods 7 (6), e2300098. 10.1002/smtd.202300098 37035956

[B91] Gu T. Wang Y. Lu Y. Cheng L. Feng L. Zhang H. (2019). Platinum nanoparticles to enable electrodynamic therapy for effective cancer treatment. Adv. Mater 31 (14), e1806803. 10.1002/adma.201806803 30734370

[B92] Guo J. Wei W. Zhao Y. Dai H. (2022). Iron oxide nanoparticles with photothermal performance and enhanced nanozyme activity for bacteria-infected wound therapy. Regen. Biomater. 9, rbac041. 10.1093/rb/rbac041 35812348 PMC9258688

[B93] Habimana O. Zanoni M. Vitale S. O'Neill T. Scholz D. Xu B. (2018). One particle, two targets: a combined action of functionalised gold nanoparticles, against Pseudomonas fluorescens biofilms. J. Colloid Interface Sci. 526, 419–428. 10.1016/j.jcis.2018.05.014 29763820

[B94] He Z. Yang H. Gu Y. Xie Y. Wu J. Wu C. (2023). Green synthesis of MOF-Mediated pH-Sensitive nanomaterial AgNPs@ZIF-8 and its application in improving the antibacterial performance of AgNPs. Int. J. Nanomedicine 18, 4857–4870. 10.2147/ijn.s418308 37662688 PMC10473413

[B95] He X. Lv Y. Lin Y. Yu H. Zhang Y. Tong Y. (2024). Platinum nanoparticles regulated V(2)C MXene nanoplatforms with NIR-II enhanced nanozyme effect for photothermal and chemodynamic anti-infective therapy. Adv. Mater 36 (25), e2400366. 10.1002/adma.202400366 38469896

[B96] Hirsch L. R. Stafford R. J. Bankson J. A. Sershen S. R. Rivera B. Price R. E. (2003). Nanoshell-mediated near-infrared thermal therapy of tumors under magnetic resonance guidance. Proc. Natl. Acad. Sci. U. S. A. 100 (23), 13549–13554. 10.1073/pnas.2232479100 14597719 PMC263851

[B97] Hoang K. N. L. McClain S. M. Meyer S. M. Jalomo C. A. Forney N. B. Murphy C. J. (2022). Site-selective modification of metallic nanoparticles. Chem. Commun. (Camb) 58 (70), 9728–9741. 10.1039/d2cc03603g 35975479

[B98] Hoshyar N. Gray S. Han H. Bao G. (2016). The effect of nanoparticle size on *in vivo* pharmacokinetics and cellular interaction. Nanomedicine (Lond) 11 (6), 673–692. 10.2217/nnm.16.5 27003448 PMC5561790

[B99] Hsiao I. L. Chang C. C. Wu C. Y. Hsieh Y. K. Chuang C. Y. Wang C. F. (2016). Indirect effects of TiO2 nanoparticle on neuron-glial cell interactions. Chem. Biol. Interact. 254, 34–44. 10.1016/j.cbi.2016.05.024 27216632

[B100] Hu Z. Li J. Li C. Zhao S. Li N. Wang Y. (2013). Folic acid-conjugated graphene–ZnO nanohybrid for targeting photodynamic therapy under visible light irradiation. J. Mater. Chem. B 1 (38), 5003–5013. 10.1039/c3tb20849d 32261090

[B101] Hu H. Feng W. Qian X. Yu L. Chen Y. Li Y. (2021). Emerging Nanomedicine-Enabled/Enhanced nanodynamic therapies beyond traditional photodynamics. Adv. Mater. 33 (12), 2005062. 10.1002/adma.202005062 33565157

[B102] Hu Z. Song X. Ding L. Cai Y. Yu L. Zhang L. (2022). Engineering Fe/Mn-doped zinc oxide nanosonosensitizers for ultrasound-activated and multiple ferroptosis-augmented nanodynamic tumor suppression. Mater Today Bio 16, 100452. 10.1016/j.mtbio.2022.100452 36245834 PMC9557028

[B103] Huang X. El-Sayed M. A. (2010). Gold nanoparticles: optical properties and implementations in cancer diagnosis and photothermal therapy. J. Adv. Res. 1 (1), 13–28. 10.1016/j.jare.2010.02.002

[B104] Huang X. El-Sayed I. H. Qian W. El-Sayed M. A. (2006). Cancer cell imaging and photothermal therapy in the near-infrared region by using gold nanorods. J. Am. Chem. Soc. 128 (6), 2115–2120. 10.1021/ja057254a 16464114

[B105] Huang X. Jain P. K. El-Sayed I. H. El-Sayed M. A. (2007). Gold nanoparticles: interesting optical properties and recent applications in cancer diagnostics and therapy. Nanomedicine (Lond) 2 (5), 681–693. 10.2217/17435889.2.5.681 17976030

[B106] Huang X. Jain P. K. El-Sayed I. H. El-Sayed M. A. (2008). Plasmonic photothermal therapy (PPTT) using gold nanoparticles. Lasers Med. Sci. 23 (3), 217–228. 10.1007/s10103-007-0470-x 17674122

[B107] Huang Y. Ferhan A. R. Gao Y. Dandapat A. Kim D. H. (2014). High-yield synthesis of triangular gold nanoplates with improved shape uniformity, tunable edge length and thickness. Nanoscale 6 (12), 6496–6500. 10.1039/c4nr00834k 24839152

[B108] Huang Y. Lai Y. Shi S. Hao S. Wei J. Chen X. (2015). Copper sulfide nanoparticles with phospholipid-PEG coating for *in vivo* near-infrared photothermal cancer therapy. Chem. Asian J. 10 (2), 370–376. 10.1002/asia.201403133 25425287

[B109] Huang Y. Li M. Huang D. Qiu Q. Lin W. Liu J. (2019). Depth-Resolved enhanced spectral-domain OCT imaging of live Mammalian embryos using gold nanoparticles as contrast agent. Small 15 (35), e1902346. 10.1002/smll.201902346 31304667

[B110] Hwang S. Nam J. Jung S. Song J. Doh H. Kim S. (2014). Gold nanoparticle-mediated photothermal therapy: current status and future perspective. Nanomedicine (Lond) 9 (13), 2003–2022. 10.2217/nnm.14.147 25343350

[B111] Ishida T. Maeda R. Ichihara M. Irimura K. Kiwada H. (2003). Accelerated clearance of PEGylated liposomes in rats after repeated injections. J. Control Release 88 (1), 35–42. 10.1016/s0168-3659(02)00462-5 12586501

[B112] Jabeen F. Najam-ul-Haq M. Javeed R. Huck C. W. Bonn G. K. (2014). Au-nanomaterials as a superior choice for near-infrared photothermal therapy. Molecules 19 (12), 20580–20593. 10.3390/molecules191220580 25501919 PMC6270707

[B113] Jain P. K. Lee K. S. El-Sayed I. H. El-Sayed M. A. (2006). Calculated absorption and scattering properties of gold nanoparticles of different size, shape, and composition: applications in biological imaging and biomedicine. J. Phys. Chem. B 110 (14), 7238–7248. 10.1021/jp057170o 16599493

[B114] Jana D. Zhao Y. (2022). Strategies for enhancing cancer chemodynamic therapy performance. Explor. (Beijing) 2 (2), 20210238. 10.1002/exp.20210238 PMC10191001 37323881

[B115] Jelic M. D. Mandic A. D. Maricic S. M. Srdjenovic B. U. (2021). Oxidative stress and its role in cancer. J. Cancer Res. Ther. 17 (1), 22–28. 10.4103/jcrt.jcrt_862_16 33723127

[B116] Jha G. Sharma R. B. Sridhar S. Hayagreev D. Sinha T. Kaur H. (2024). Nanoparticle-Based therapies for cardiovascular diseases: a literature review of recent advances and clinical potential. Cureus 16 (10), e72808. 10.7759/cureus.72808 39552990 PMC11569831

[B117] Ji B. Wei M. Yang B. (2022). Recent advances in nanomedicines for photodynamic therapy (PDT)-driven cancer immunotherapy. Theranostics 12 (1), 434–458. 10.7150/thno.67300 34987658 PMC8690913

[B118] Jiang W. Kim B. Y. S. Rutka J. T. Chan W. C. W. (2008). Nanoparticle-mediated cellular response is size-dependent. Nat. Nanotechnol. 3 (3), 145–150. 10.1038/nnano.2008.30 18654486

[B119] Jiang Z. Jiang Z. Jiang Y. Cheng Y. Yao Q. Chen R. (2023). Fe-involved nanostructures act as photothermal transduction agents in cancer photothermal therapy. Colloids Surf. B Biointerfaces 228, 113438. 10.1016/j.colsurfb.2023.113438 37421763

[B120] Kafrouni L. Savadogo O. (2016). Recent progress on magnetic nanoparticles for magnetic hyperthermia. Prog. Biomater. 5 (3-4), 147–160. 10.1007/s40204-016-0054-6 27995583 PMC5304434

[B121] Kah G. Chandran R. Abrahamse H. (2023). Biogenic silver nanoparticles for targeted cancer therapy and enhancing photodynamic therapy. Cells 12, 2012. 10.3390/cells12152012 PMC10417642 37566091

[B122] Kambic H. E. Reyes E. Manning T. Waters K. C. Reger S. I. (1993). Influence of AC and DC electrical stimulation on wound healing in pigs: a biomechanical analysis. J. Invest. Surg. 6 (6), 535–543. 10.3109/08941939309141644 8123615

[B123] Kim M.-S. Louis K. M. Pedersen J. A. Hamers R. J. Peterson R. E. Heideman W. (2014). Using citrate-functionalized TiO 2 nanoparticles to study the effect of particle size on zebrafish embryo toxicity. Analyst 139 (5), 964–972. 10.1039/c3an01966g 24384696

[B124] Kim M. Jung H. Y. Park H. J. (2015). Topical PDT in the treatment of benign skin diseases: principles and new applications. Int. J. Mol. Sci. 16 (10), 23259–23278. 10.3390/ijms161023259 26404243 PMC4632697

[B125] Kolosnjaj-Tabi J. Di Corato R. Lartigue L. Marangon I. Guardia P. Silva A. K. A. (2014). Heat-generating iron oxide nanocubes: subtle “destructurators” of the tumoral microenvironment. ACS Nano 8 (5), 4268–4283. 10.1021/nn405356r 24738788

[B126] Kubrak T. Karakuła M. Czop M. Kawczyk-Krupka A. Aebisher D. (2022). Advances in management of bladder cancer-the role of photodynamic therapy. Molecules 27 (3), 731. 10.3390/molecules27030731 35163996 PMC8838614

[B127] Kwiatkowski S. Knap B. Przystupski D. Saczko J. Kędzierska E. Knap-Czop K. (2018). Photodynamic therapy – mechanisms, photosensitizers and combinations. Biomed. and Pharmacother. 106, 1098–1107. 10.1016/j.biopha.2018.07.049 30119176

[B128] Lafond M. Yoshizawa S. Umemura S. I. (2019). Sonodynamic therapy: advances and challenges in clinical translation. J. Ultrasound Med. 38 (3), 567–580. 10.1002/jum.14733 30338863

[B129] Lee S. A. Link S. (2021). Chemical interface damping of surface plasmon resonances. Acc. Chem. Res. 54 (8), 1950–1960. 10.1021/acs.accounts.0c00872 33788547

[B130] Lee G. Lee Y. J. Kim Y. J. Park Y. (2023). Synthesis of Au-Ag bimetallic nanoparticles using Korean red ginseng (Panax ginseng Meyer) root extract for chemo-photothermal anticancer therapy. Arch. Pharm. Res. 46 (8), 659–678. 10.1007/s12272-023-01457-y 37592169

[B131] Li J. Ai Y. Wang L. Bu P. Sharkey C. C. Wu Q. (2016). Targeted drug delivery to circulating tumor cells via platelet membrane-functionalized particles. Biomaterials 76, 52–65. 10.1016/j.biomaterials.2015.10.046 26519648 PMC4662903

[B132] Li Q. Kartikowati C. W. Horie S. Ogi T. Iwaki T. Okuyama K. (2017). Correlation between particle size/domain structure and magnetic properties of highly crystalline Fe(3)O(4) nanoparticles. Sci. Rep. 7 (1), 9894. 10.1038/s41598-017-09897-5 28855564 PMC5577113

[B133] Li H. Jin K. Luo M. Wang X. Zhu X. Liu X. (2019). Size dependency of circulation and biodistribution of biomimetic nanoparticles: red blood cell membrane-coated nanoparticles. Cells 8 (8), 881. 10.3390/cells8080881 31412631 PMC6721642

[B134] Li C. Wang J. Liu L. (2020). Alzheimer's therapeutic strategy: photoactive platforms for suppressing the aggregation of amyloid β protein. Front. Chem. 8, 509. 10.3389/fchem.2020.00509 32793545 PMC7385073

[B135] Li X. Xia S. Ji R. Zhan W. Zhou W. (2021). Evaluation of microwave ablation in 4T1 breast tumor by a novel VEFGR2 targeted ultrasound contrast agents. Front. Oncol. 11, 690152. 10.3389/fonc.2021.690152 34354946 PMC8329532

[B136] Li Y. Fu R. Duan Z. Zhu C. Fan D. (2022a). Mussel-inspired adhesive bilayer hydrogels for bacteria-infected wound healing via NIR-enhanced nanozyme therapy. Colloids Surf. B Biointerfaces 210, 112230. 10.1016/j.colsurfb.2021.112230 34871820

[B137] Li K. Ma X. He S. Wang L. Yang X. Zhang G. (2022b). Ultrathin nanosheet-supported Ag@Ag(2)O core-shell nanoparticles with vastly enhanced photothermal conversion efficiency for NIR-II-Triggered photothermal therapy. ACS Biomater. Sci. Eng. 8 (2), 540–550. 10.1021/acsbiomaterials.1c01291 35107009

[B138] Li H. Yang X. Wang Z. She W. Liu Y. Huang L. (2023a). A Near-Infrared-II Fluorescent Nanocatalyst for enhanced CAR T cell therapy against solid tumor by immune reprogramming. ACS Nano 17 (12), 11749–11763. 10.1021/acsnano.3c02592 37319120

[B139] Li Y. Liu Y. Xu J. Chen D. Wu T. Cao Y. (2023b). Macrophage-Cancer hybrid membrane-camouflaged nanoplatforms for HIF-1α gene silencing-enhanced sonodynamic therapy of glioblastoma. ACS Appl. Mater Interfaces 15 (26), 31150–31158. 10.1021/acsami.3c03001 37338326

[B140] Li X. Qi J. Wang J. Hu W. Zhou W. Wang Y. (2024a). Nanoparticle technology for mRNA: delivery strategy, clinical application and developmental landscape. Theranostics 14 (2), 738–760. 10.7150/thno.84291 PMC10758055 38169577

[B141] Li Y. Chang P. Xu L. Zhu Z. Hu M. Cen J. (2024b). TiO2-Nanoparticle-Enhanced sonodynamic therapy for prevention of posterior capsular opacification and ferroptosis exploration of its mechanism. Invest. Ophthalmol. Vis. Sci. 65 (12), 24. 10.1167/iovs.65.12.24 39417751 PMC11500051

[B142] Li J. Zhao X. Xia Y. Qi X. Jiang C. Xiao Y. (2024c). Strontium-Containing piezoelectric biofilm promotes dentin tissue regeneration. Adv. Mater 36 (21), e2313419. 10.1002/adma.202313419 38335452

[B143] Liang K. Chen H. (2020). Protein-based nanoplatforms for tumor imaging and therapy. Wiley Interdiscip. Rev. Nanomed Nanobiotechnol 12 (4), e1616. 10.1002/wnan.1616 31999083

[B144] Liang S. Zhu L. Gai G. Yao Y. Huang J. Ji X. (2014). Synthesis of morphology-controlled ZnO microstructures via a microwave-assisted hydrothermal method and their gas-sensing property. Ultrason. Sonochem 21 (4), 1335–1342. 10.1016/j.ultsonch.2014.02.007 24618526

[B145] Liang J. Qiao X. Qiu L. Xu H. Xiang H. Ding H. (2023). Engineering Versatile nanomedicines for ultrasonic Tumor immunotherapy. Adv. Sci. (Weinh) 11, e2305392. 10.1002/advs.202305392 38041509 PMC10797440

[B146] Liao S. Zhang Y. Pan X. Zhu F. Jiang C. Liu Q. (2019). Antibacterial activity and mechanism of silver nanoparticles against multidrug-resistant Pseudomonas aeruginosa. Int. J. Nanomedicine 14, 1469–1487. 10.2147/IJN.S191340 30880959 PMC6396885

[B147] Lim H. K. Asharani P. V. Hande M. P. (2012). Enhanced genotoxicity of silver nanoparticles in DNA repair deficient Mammalian cells. Front. Genet. 3, 104. 10.3389/fgene.2012.00104 22707954 PMC3374476

[B148] Lin Q. Hong X. Zhang D. Jin H. (2019). Biosynthesis of size-controlled gold nanoparticles using M. lucida leaf extract and their penetration studies on human skin for plastic surgery applications. J. Photochem. Photobiol. B Biol. 199, 111591. 10.1016/j.jphotobiol.2019.111591 31514102

[B149] Lin Y. Xie R. Yu T. (2024). Photodynamic therapy for atherosclerosis: past, present, and future. Pharmaceutics 16 (6), 729. 10.3390/pharmaceutics16060729 38931851 PMC11206729

[B150] Ling J. Wang K. Wang Z. Huang H. Zhang G. (2020). Enhanced piezoelectric-induced catalysis of SrTiO(3) nanocrystal with well-defined facets under ultrasonic vibration. Ultrason. Sonochem 61, 104819. 10.1016/j.ultsonch.2019.104819 31669844

[B151] Liu J. Vipulanandan C. (2013). Effects of Au/Fe and Fe nanoparticles on Serratia bacterial growth and production of biosurfactant. Mater. Sci. Eng. C 33 (7), 3909–3915. 10.1016/j.msec.2013.05.026 23910295

[B152] Liu P. Qin R. Fu G. Zheng N. (2017). Surface coordination chemistry of metal nanomaterials. J. Am. Chem. Soc. 139 (6), 2122–2131. 10.1021/jacs.6b10978 28085260

[B153] Liu L. Jin R. Duan J. Yang L. Cai Z. Zhu W. (2020a). Bioactive iron oxide nanoparticles suppress osteoclastogenesis and ovariectomy-induced bone loss through regulating the TRAF6-p62-CYLD signaling complex. Acta Biomater. 103, 281–292. 10.1016/j.actbio.2019.12.022 31866569

[B154] Liu X. Jin Y. Liu T. Yang S. Zhou M. Wang W. (2020b). Iron-Based theranostic nanoplatform for improving chemodynamic therapy of cancer. ACS Biomater. Sci. Eng. 6 (9), 4834–4845. 10.1021/acsbiomaterials.0c01009 33455215

[B155] Liu W. Dong X. Liu Y. Sun Y. (2021). Photoresponsive materials for intensified modulation of Alzheimer's amyloid-β protein aggregation: a review. Acta Biomater. 123, 93–109. 10.1016/j.actbio.2021.01.018 33465508

[B156] Liu G. Liu M. Li X. Ye X. Cao K. Liu Y. (2023a). Peroxide-Simulating and GSH-Depleting nanozyme for enhanced Chemodynamic/Photodynamic therapy via induction of Multisource ROS. ACS Appl. Mater Interfaces 15 (41), 47955–47968. 10.1021/acsami.3c09873 37812458

[B157] Liu J. Wen C. Hu M. Leng N. Lin X. C. (2023b). Mechanism underlying the effect of MnO(2) nanosheets for A549 cell chemodynamic therapy. Mikrochim. Acta 190 (10), 381. 10.1007/s00604-023-05974-x 37697041

[B158] Liu T. Chai S. Li M. Chen X. Xie Y. Zhao Z. (2024). A nanoparticle-based sonodynamic therapy reduces Helicobacter pylori infection in mouse without disrupting gut microbiota. Nat. Commun. 15 (1), 844. 10.1038/s41467-024-45156-8 38286999 PMC10825188

[B159] Llovet J. M. Vilana R. Brú C. Bianchi L. Salmeron J. M. Boix L. (2001). Increased risk of tumor seeding after percutaneous radiofrequency ablation for single hepatocellular carcinoma. Hepatology 33 (5), 1124–1129. 10.1053/jhep.2001.24233 11343240

[B160] Longmire M. R. Ogawa M. Choyke P. L. Kobayashi H. (2011). Biologically optimized nanosized molecules and particles: more than just size. Bioconjug Chem. 22 (6), 993–1000. 10.1021/bc200111p 21513351 PMC3116041

[B161] Lorenzana-Vázquez G. Pavel I. Meléndez E. (2023). Gold nanoparticles functionalized with 2-Thiouracil for antiproliferative and photothermal therapies in breast cancer cells. Molecules 28 (11), 4453. 10.3390/molecules28114453 37298929 PMC10254252

[B162] Lv Z. He S. Wang Y. Zhu X. (2021). Noble metal nanomaterials for NIR-Triggered photothermal therapy in cancer. Adv. Healthc. Mater. 10 (6), 2001806. 10.1002/adhm.202001806 33470542

[B163] Ma J. Liu X. Wang R. Zhang J. Jiang P. Wang Y. (2020). Bimetallic core–shell nanostars with Tunable Surface Plasmon resonance for Surface-Enhanced raman scattering. ACS Appl. Nano Mater. 3 (11), 10885–10894. 10.1021/acsanm.0c02144

[B164] Ma R. Zhang Q. Wang Y. Xu Z. (2024). Structural engineering of mitochondria-targeted Au-Ag(2)S photosensitizers for enhanced photodynamic and photothermal therapy. J. Mater Chem. B 12 (31), 7646–7658. 10.1039/d4tb00533c 39007565

[B165] Mageswari K. Prabukanthan P. Madhavan J. (2023). Microwave-assisted synthesis of ZnO nanoparticles using different capping agents and their photocatalytic application. Environ. Sci. Pollut. Res. Int. 30 (14), 40174–40188. 10.1007/s11356-022-25097-9 36607582

[B166] Maji S. K. Yu S. Chung K. Sekkarapatti Ramasamy M. Lim J. W. Wang J. (2018). Synergistic nanozymetic activity of hybrid gold Bipyramid-Molybdenum Disulfide Core@Shell nanostructures for two-photon imaging and anticancer therapy. ACS Appl. Mater Interfaces 10 (49), 42068–42076. 10.1021/acsami.8b15443 30462488

[B167] Malá Z. Žárská L. Bajgar R. Bogdanová K. Kolář M. Panáček A. (2021). The application of antimicrobial photodynamic inactivation on methicillin-resistant S. aureus and ESBL-producing K. pneumoniae using porphyrin photosensitizer in combination with silver nanoparticles. Photodiagnosis Photodyn. Ther. 33, 102140. 10.1016/j.pdpdt.2020.102140 33307229

[B168] McShan D. Ray P. C. Yu H. (2014). Molecular toxicity mechanism of nanosilver. J. Food Drug Anal. 22 (1), 116–127. 10.1016/j.jfda.2014.01.010 24673909 PMC4281024

[B169] Medina M. A. Oza G. Ángeles-Pascual A. González M M. Antaño-López R. Vera A. (2020). Synthesis, characterization and magnetic hyperthermia of monodispersed cobalt ferrite nanoparticles for cancer therapeutics. Molecules 25 (19), 4428. 10.3390/molecules25194428 32992439 PMC7583941

[B170] Meng T. Jiang R. Wang S. Li J. Zhang F. Lee J. H. (2020). Stem cell membrane-coated Au-Ag-PDA nanoparticle-guided photothermal acne therapy. Colloids Surfaces B Biointerfaces 192, 111145. 10.1016/j.colsurfb.2020.111145 32480049

[B171] Meng X. Sun S. Gong C. Yang J. Yang Z. Zhang X. (2022). Ag-Doped metal-organic frameworks' heterostructure for sonodynamic therapy of deep-seated cancer and bacterial infection. ACS Nano. 17 (2), 1174–1186. 10.1021/acsnano.2c08687 36583572

[B172] Meng J. Zhang P. Liu Q. Ran P. Xie S. Wei J. (2023). Pyroelectric Janus nanomotors for synergistic electrodynamic-photothermal-antibiotic therapies of bacterial infections. Acta Biomater. 162, 20–31. 10.1016/j.actbio.2023.03.012 36931421

[B173] Mergani A. Meurer M. Wiebe E. Dümmer K. Wirz K. Lehmann J. (2023). Alteration of cholesterol content and oxygen level in intestinal organoids after infection with Staphylococcus aureus. Faseb J. 37 (12), e23279. 10.1096/fj.202300799r 37902583

[B174] Min Q. Gao Y. Wang Y. (2024). Bioelectricity in dental medicine: a narrative review. Biomed. Eng. Online 23 (1), 3. 10.1186/s12938-023-01189-6 PMC10765628 38172866

[B175] Morais M. Machado V. Figueiredo P. Dias F. Craveiro R. Lencart J. (2023). Silver nanoparticles (AgNPs) as enhancers of everolimus and radiotherapy sensitivity on clear cell renal cell carcinoma. Antioxidants (Basel) 12 (12), 2051. 10.3390/antiox12122051 38136171 PMC10741111

[B176] Moskovits M. (2008). Surface roughness and the enhanced intensity of Raman scattering by molecules adsorbed on metals. J. Chem. Phys. 69 (9), 4159–4161. 10.1063/1.437095

[B177] Myronov P. Bugaiov V. Holubnycha V. Sikora V. Deineka V. Lyndin M. (2020). Low-frequency ultrasound increase effectiveness of silver nanoparticles in a purulent wound model. Biomed. Eng. Lett. 10 (4), 621–631. 10.1007/s13534-020-00174-5 33194252 PMC7655885

[B178] Najim Obaid M. Sabr O. H. Jawad Kadhim B. (2024). The effect of MgO nanoparticle on PVA/PEG-based membranes for potential application in wound healing. J. Biomater. Sci. Polym. Ed. 35 (13), 1963–1977. 10.1080/09205063.2024.2364526 38949409

[B179] Nasir A. Rehman M. U. Khan T. Husn M. Khan M. Khan A. (2024). Advances in nanotechnology-assisted photodynamic therapy for neurological disorders: a comprehensive review. Artif. Cells Nanomed Biotechnol. 52 (1), 84–103. 10.1080/21691401.2024.2304814 38235991

[B180] Nguyen L. H. Phong P. Nam P. Manh D. Thanh N. Tung L. (2021). The role of anisotropy in distinguishing domination of Néel or brownian relaxation contribution to magnetic inductive heating: orientations for biomedical applications. Mater. (Basel) 14 (8), 1875. 10.3390/ma14081875 PMC8070233 33918815

[B181] Nie Y. Zhang W. Xiao W. Zeng W. Chen T. Huang W. (2022). Novel biodegradable two-dimensional vanadene augmented photoelectro-fenton process for cancer catalytic therapy. Biomaterials 289, 121791. 10.1016/j.biomaterials.2022.121791 36084481

[B182] Ninomiya K. Ogino C. Oshima S. Sonoke S. Kuroda S. i. Shimizu N. (2012). Targeted sonodynamic therapy using protein-modified TiO2 nanoparticles. Ultrason. Sonochem 19 (3), 607–614. 10.1016/j.ultsonch.2011.09.009 22019790

[B183] Noqta O. A. Aziz A. A. Usman I. A. Bououdina M. (2019). Recent advances in iron oxide nanoparticles (IONPs): synthesis and surface modification for biomedical applications. J. Supercond. Nov. Magnetism 32, 779–795. 10.1007/s10948-018-4939-6

[B184] Ou X. Liu Y. Zhang M. Hua L. Zhan S. (2021). Plasmonic gold nanostructures for biosensing and bioimaging. Mikrochim. Acta 188 (9), 304. 10.1007/s00604-021-04964-1 34435258 PMC8386900

[B185] Ouyang Y. Fadeev M. Zhang P. Carmieli R. Li J. Sohn Y. S. (2022). Aptamer-Modified Au Nanoparticles: functional Nanozyme Bioreactors for Cascaded Catalysis and Catalysts for Chemodynamic Treatment of Cancer Cells. ACS Nano 16 (11), 18232–18243. 10.1021/acsnano.2c05710 36286233 PMC9706657

[B186] Overchuk M. Weersink R. A. Wilson B. C. Zheng G. (2023). Photodynamic and photothermal therapies: synergy opportunities for nanomedicine. ACS Nano 17 (9), 7979–8003. 10.1021/acsnano.3c00891 37129253 PMC10173698

[B187] Pan Y. Ma X. Liu C. Xing J. Zhou S. Parshad B. (2021). Retinoic acid-loaded dendritic polyglycerol-conjugated gold nanostars for targeted photothermal therapy in breast cancer stem cells. ACS Nano 15 (9), 15069–15084. 10.1021/acsnano.1c05452 34420298

[B188] Pang C. Zhang P. Mu Y. Ren J. Zhao B. (2020). Transformation and cytotoxicity of surface-modified silver nanoparticles undergoing long-term aging. Nanomater. (Basel) 10 (11), 2255. 10.3390/nano10112255 33203023 PMC7697416

[B189] Park T. Lee S. Amatya R. Cheong H. Moon C. Kwak H. D. (2020). ICG-Loaded PEGylated BSA-silver nanoparticles for effective photothermal cancer therapy. Int. J. Nanomedicine 15, 5459–5471. 10.2147/IJN.S255874 32801700 PMC7406329

[B190] Pawar K. Kaul G. (2014). Toxicity of titanium oxide nanoparticles causes functionality and DNA damage in buffalo (Bubalus bubalis) sperm *in vitro* . Toxicol. Industrial Health 30 (6), 520–533. 10.1177/0748233712462475 23064765

[B191] Peng H. Li S. Xing J. Yang F. Wu A. (2023). Surface plasmon resonance of Au/Ag metals for the photoluminescence enhancement of lanthanide ion Ln(3+) doped upconversion nanoparticles in bioimaging. J. Mater Chem. B 11 (24), 5238–5250. 10.1039/d2tb02251f 36477984

[B192] Penon O. Marín M. J. Amabilino D. B. Russell D. A. Pérez-García L. (2016). Iron oxide nanoparticles functionalized with novel hydrophobic and hydrophilic porphyrins as potential agents for photodynamic therapy. J. Colloid Interface Sci. 462, 154–165. 10.1016/j.jcis.2015.09.060 26454374

[B193] Perrault S. D. Walkey C. Jennings T. Fischer H. C. Chan W. C. W. (2009). Mediating tumor targeting efficiency of nanoparticles through design. Nano Lett. 9 (5), 1909–1915. 10.1021/nl900031y 19344179

[B194] Pinzaru I. Coricovac D. Dehelean C. Moacă E. A. Mioc M. Baderca F. (2018). Stable PEG-coated silver nanoparticles - a comprehensive toxicological profile. Food Chem. Toxicol. 111, 546–556. 10.1016/j.fct.2017.11.051 29191727

[B195] Popović Z. Liu W. Chauhan V. P. Lee J. Wong C. Greytak A. B. (2010). A nanoparticle size series for *in vivo* fluorescence imaging. Angew. Chem. Int. Ed. Engl. 49 (46), 8649–8652. 10.1002/anie.201003142 20886481 PMC3035057

[B196] Prejanò M. Alberto M. E. De Simone B. C. Marino T. Toscano M. Russo N. (2023). Sulphur- and Selenium-for-Oxygen replacement as a strategy to obtain dual type I/Type II photosensitizers for photodynamic therapy. Molecules 28 (7), 3153. 10.3390/molecules28073153 PMC10095929 37049916

[B197] Qi M. Chi M. Sun X. Xie X. Weir M. D. Oates T. W. (2019). Novel nanomaterial-based antibacterial photodynamic therapies to combat oral bacterial biofilms and infectious diseases. Int. J. Nanomedicine 14, 6937–6956. 10.2147/IJN.S212807 31695368 PMC6718167

[B198] Qiao Z. Zhang K. Liu J. Cheng D. Yu B. Zhao N. (2022). Biomimetic electrodynamic nanoparticles comprising ginger-derived extracellular vesicles for synergistic anti-infective therapy. Nat. Commun. 13 (1), 7164. 10.1038/s41467-022-34883-5 36418895 PMC9684156

[B199] Quan Z. Wang S. Xie H. Zhang J. Duan R. Li M. (2024). ROS Regulation in CNS Disorder Therapy: Unveiling the Dual Roles of Nanomedicine. Small. 2410031. 10.1002/smll.202410031 39676433

[B200] Quinteros M. A. Viviana C. A. Onnainty R. Mary V. S. Theumer M. G. Granero G. E. (2018). Biosynthesized silver nanoparticles: decoding their mechanism of action in Staphylococcus aureus and Escherichia coli. Int. J. Biochem. and Cell. Biol. 104, 87–93. 10.1016/j.biocel.2018.09.006 30243952

[B201] Raveendran S. Sen A. Maekawa T. Kumar D. S. (2017). Ultra-fast microwave aided synthesis of gold nanocages and structural maneuver studies. Nano Res. 10 (3), 1078–1091. 10.1007/s12274-016-1368-3

[B202] Ren Q. Yi C. Pan J. Sun X. Huang X. (2022). Smart Fe(3)O(4)@ZnO core-shell nanophotosensitizers potential for combined chemo and photodynamic skin cancer therapy controlled by UVA radiation. Int. J. Nanomedicine 17, 3385–3400. 10.2147/ijn.s372377 35937080 PMC9355344

[B203] Rodríguez-Barajas N. Anaya-Esparza L. M. Villagrán-de la Mora Z. Sánchez-Burgos J. A. Pérez-Larios A. (2022). Review of therapies using TiO(2) nanomaterials for increased anticancer capability. Anticancer Agents Med. Chem. 22 (12), 2241–2254. 10.2174/1871520622666211228112631 34963437

[B204] Saeed M. Ren W. Wu A. (2018). Therapeutic applications of iron oxide based nanoparticles in cancer: basic concepts and recent advances. Biomater. Sci. 6 (4), 708–725. 10.1039/c7bm00999b 29363682

[B205] Salatin S. Maleki Dizaj S. Yari Khosroushahi A. (2015). Effect of the surface modification, size, and shape on cellular uptake of nanoparticles. Cell. Biol. Int. 39 (8), 881–890. 10.1002/cbin.10459 25790433

[B206] Sappati K. K. Bhadra S. (2018). Piezoelectric polymer and paper substrates: a review. Sensors (Basel) 18 (11), 3605. 10.3390/s18113605 30355961 PMC6263872

[B207] Sardar R. Funston A. M. Mulvaney P. Murray R. W. (2009). Gold nanoparticles: past, present, and future. Langmuir 25 (24), 13840–13851. 10.1021/la9019475 19572538

[B208] Schaumann G. E. Philippe A. Bundschuh M. Metreveli G. Klitzke S. Rakcheev D. (2015). Understanding the fate and biological effects of Ag- and TiO2-nanoparticles in the environment: the quest for advanced analytics and interdisciplinary concepts. Sci. Total Environ. 535, 3–19. 10.1016/j.scitotenv.2014.10.035 25455109

[B209] Sekar D. Falcioni M. L. Barucca G. Falcioni G. (2014). DNA damage and repair following *in vitro* exposure to two different forms of titanium dioxide nanoparticles on trout erythrocyte. Environ. Toxicology 29 (1), 117–127. 10.1002/tox.20778 22012887

[B210] Seok S. H. Cho W. S. Park J. S. Na Y. Jang A. Kim H. (2013). Rat pancreatitis produced by 13-week administration of zinc oxide nanoparticles: biopersistence of nanoparticles and possible solutions. J. Applied Toxicol. 33 (10), 1089–1096. 10.1002/jat.2862 23408656

[B211] Shen S. Wu L. Liu J. Xie M. Shen H. Qi X. (2015). Core-shell structured Fe3O4@TiO2-doxorubicin nanoparticles for targeted chemo-sonodynamic therapy of cancer. Int. J. Pharm. 486 (1-2), 380–388. 10.1016/j.ijpharm.2015.03.070 25841570

[B212] Shen S. Kong F. Guo X. Wu L. Shen H. Xie M. (2013). CMCTS stabilized Fe3O4 particles with extremely low toxicity as highly efficient near-infrared photothermal agents for *in vivo* tumor ablation. Nanoscale 5 (17), 8056–8066. 10.1039/c3nr01447a 23873020

[B213] Shen S. Feng J. Song X. Xiang W. (2022). Efficacy of photodynamic therapy for warts induced by human papilloma virus infection: a systematic review and meta-analysis. Photodiagnosis Photodyn. Ther. 39, 102913. 10.1016/j.pdpdt.2022.102913 35605923

[B214] Sheng Y. Ren Q. Tao C. Wen M. Qu P. Yu N. (2023). Construction of PEGylated chlorin e6@CuS-Pt theranostic nanoplatforms for nanozymes-enhanced photodynamic-photothermal therapy. J. Colloid Interface Sci. 645, 122–132. 10.1016/j.jcis.2023.04.092 37146376

[B215] Shevtsov M. A. Parr M. A. Ryzhov V. A. Zemtsova E. G. Arbenin A. Y. Ponomareva A. N. (2016). Zero-valent Fe confined mesoporous silica nanocarriers (Fe(0) @ MCM-41) for targeting experimental orthotopic glioma in rats. Sci. Rep. 6, 29247. 10.1038/srep29247 27386761 PMC4937429

[B216] Shi H. Lin S. Wang Y. Lou J. Hu Y. Chen Y. (2022). Ruthenium photosensitizer anchored gold nanorods for synergistic photodynamic and photothermal therapy. Dalton Trans. 51 (17), 6846–6854. 10.1039/d2dt00365a 35438705

[B217] Simelane N. W. N. Abrahamse H. (2024). Actively targeted photodynamic therapy in multicellular colorectal cancer spheroids via functionalised gold nanoparticles. Artif. Cells Nanomed Biotechnol. 52 (1), 309–320. 10.1080/21691401.2024.2357693 38781462

[B218] Singh C. Mehata A. K. Priya V. Malik A. K. Setia A. Suseela M. N. L. (2022). Bimetallic Au–Ag nanoparticles: advanced nanotechnology for tackling antimicrobial resistance. Molecules 27, 7059. 10.3390/molecules27207059 PMC9610600 36296652

[B219] Son S. Kim J. H. Wang X. Zhang C. Yoon S. A. Shin J. (2020). Multifunctional sonosensitizers in sonodynamic cancer therapy. Chem. Soc. Rev. 49 (11), 3244–3261. 10.1039/c9cs00648f 32337527

[B220] Song X. R. Goswami N. Yang H. H. Xie J. (2016a). Functionalization of metal nanoclusters for biomedical applications. Analyst 141 (11), 3126–3140. 10.1039/c6an00773b 27146244

[B221] Song J. Qu J. Swihart M. T. Prasad P. N. (2016b). Near-IR responsive nanostructures for nanobiophotonics: emerging impacts on nanomedicine. Nanomedicine 12 (3), 771–788. 10.1016/j.nano.2015.11.009 26656629

[B222] Song F. Jiang D. Wang T. Wang Y. Lou Y. Zhang Y. (2017). Mechanical stress regulates osteogenesis and adipogenesis of rat mesenchymal stem cells through PI3K/Akt/GSK-3β/β-Catenin signaling pathway. Biomed. Res. Int. 2017, 6027402. 10.1155/2017/6027402 28286769 PMC5329655

[B223] Song P. Jin S. Cao Y. Zhang S. Yin N. Zhang H. (2023). Multifunctional biocompatible Ni/Ni-P nanospheres for anti-tumor “neoadjuvant phototherapy” combining photothermal therapy and photodynamic therapy. J. Mater Chem. B 11 (41), 10019–10028. 10.1039/d3tb01802d 37850304

[B224] Srinivasan E. S. Liu Y. Odion R. A. Chongsathidkiet P. Wachsmuth L. P. Haskell-Mendoza A. P. (2023). Gold nanostars obviate limitations to Laser Interstitial Thermal Therapy (LITT) for the treatment of intracranial tumors. Clin. Cancer Res. 29 (16), 3214–3224. 10.1158/1078-0432.ccr-22-1871 37327318 PMC10425731

[B225] Srividhyaa K. Ranjani S. Hemalatha S. (2023). Citrus limon phytocompounds decorated nanoparticles control poultry pathogens. Arch. Microbiol. 205 (4), 124. 10.1007/s00203-023-03462-7 36941518

[B226] Su Z. Xu D. Hu X. Zhu W. Kong L. Qian Z. (2024). Biodegradable oxygen-evolving metalloantibiotics for spatiotemporal sono-metalloimmunotherapy against orthopaedic biofilm infections. Nat. Commun. 15 (1), 8058. 10.1038/s41467-024-52489-x 39277594 PMC11401848

[B227] Su G. L. Peng Y. J. Ruan H. Z. Cheng J. Deng T. Zhang Y. F. (2025). Regulating periodontal disease with smart stimuli-responsive systems: antimicrobial activity, immunomodulation, periodontium regeneration. Mater Today Bio 32, 101863. 10.1016/j.mtbio.2025.101863 40496719 PMC12148678

[B228] Sui C. Tan R. Chen Y. Yin G. Wang Z. Xu W. (2021). MOFs-Derived Fe-N codoped carbon nanoparticles as O(2)-Evolving reactor and ROS generator for CDT/PDT/PTT synergistic treatment of tumors. Bioconjug Chem. 32 (2), 318–327. 10.1021/acs.bioconjchem.0c00694 33543921

[B229] Suk J. S. Xu Q. Kim N. Hanes J. Ensign L. M. (2016). PEGylation as a strategy for improving nanoparticle-based drug and gene delivery. Adv. Drug Deliv. Rev. 99, 28–51. 10.1016/j.addr.2015.09.012 26456916 PMC4798869

[B230] Sun Y. Wang Q. Chen J. Liu L. Ding L. Shen M. (2017). Temperature-Sensitive gold nanoparticle-coated Pluronic-PLL nanoparticles for drug delivery and chemo-photothermal therapy. Theranostics 7 (18), 4424–4444. 10.7150/thno.18832 29158837 PMC5695141

[B231] Sun Y. Xu W. Jiang C. Zhou T. Wang Q. A L. (2022). Gold nanoparticle decoration potentiate the antibacterial enhancement of TiO(2) nanotubes via sonodynamic therapy against peri-implant infections. Front. Bioeng. Biotechnol. 10, 1074083. 10.3389/fbioe.2022.1074083 36466357 PMC9713247

[B232] Sun J. Zhao D. Wang Y. Chen P. Xu C. Lei H. (2023). Temporal immunomodulation via wireless programmed electric cues achieves optimized diabetic bone regeneration. ACS Nano 17 (22), 22830–22843. 10.1021/acsnano.3c07607 37943709

[B233] Suriyanto Ng E. Y. Kumar S. D. (2017). Physical mechanism and modeling of heat generation and transfer in magnetic fluid hyperthermia through Néelian and Brownian relaxation: a review. Biomed. Eng. Online 16 (1), 36. 10.1186/s12938-017-0327-x 28335790 PMC5364696

[B234] Tai Y. W. Chiu Y. C. Wu P. T. Yu J. Chin Y. C. Wu S. P. (2018). Degradable NIR-PTT nanoagents with a potential Cu@Cu(2)O@Polymer structure. ACS Appl. Mater Interfaces 10 (6), 5161–5174. 10.1021/acsami.7b15109 29359551

[B235] Tang L. Yang X. Yin Q. Cai K. Wang H. Chaudhury I. (2014). Investigating the optimal size of anticancer nanomedicine. Proc. Natl. Acad. Sci. U. S. A. 111 (43), 15344–15349. 10.1073/pnas.1411499111 25316794 PMC4217425

[B236] Tang Z. Liu Y. He M. Bu W. (2019). Chemodynamic therapy: tumour microenvironment-mediated fenton and fenton-like reactions. Angew. Chem. Int. Ed. Engl. 58 (4), 946–956. 10.1002/anie.201805664 30048028

[B237] Tansi F. L. Maduabuchi W. O. Hirsch M. Southern P. Hattersley S. Quaas R. (2021). Deep-tissue localization of magnetic field hyperthermia using pulse sequencing. Int. J. Hyperth. 38 (1), 743–754. 10.1080/02656736.2021.1912412 33941016

[B238] Tao Y. Zhuang W. Fan W. Zhou L. Fan L. Qin H. (2025). Dual-functional silver nanoparticle-enhanced ZnO nanorods for improved reactive oxygen species generation and cancer treatment. iScience 28 (2), 111858. 10.1016/j.isci.2025.111858 PMC11867527 40017508

[B239] Tong X. Wang Z. Sun X. Song J. Jacobson O. Niu G. (2016). Size dependent kinetics of gold nanorods in EPR mediated tumor delivery. Theranostics 6 (12), 2039–2051. 10.7150/thno.17098 27698939 PMC5039679

[B240] Tran P.H.-L. Tran T. T. D. Vo T. V. Lee B. J. (2012). Promising iron oxide-based magnetic nanoparticles in biomedical engineering. Archives Pharmacal Res. 35 (12), 2045–2061. 10.1007/s12272-012-1203-7 23263800

[B241] Unterweger H. Subatzus D. Tietze R. Janko C. Poettler M. Stiegelschmitt A. (2015). Hypericin-bearing magnetic iron oxide nanoparticles for selective drug delivery in photodynamic therapy. Int. J. Nanomedicine 10, 6985–6996. 10.2147/IJN.S92336 26648714 PMC4648594

[B242] Wang Y. Miron R. J. Zhang X. Zeng H. Zhang Y. (2024). Nanocages and cell-membrane display technology as smart biomaterials. Periodontology 2000 94 (1), 180–191. 10.1111/prd.12514 37614160

[B243] Wang J. Zhou G. Chen C. Yu H. Wang T. Ma Y. (2007). Acute toxicity and biodistribution of different sized titanium dioxide particles in mice after oral administration. Toxicol. Letters 168 (2), 176–185. 10.1016/j.toxlet.2006.12.001 17197136

[B244] Wang D. Zhao L. Guo L. H. Zhang H. (2014). Online detection of reactive oxygen species in ultraviolet (UV)-Irradiated nano-TiO2 suspensions by continuous flow chemiluminescence. Anal. Chem. 86 (21), 10535–10539. 10.1021/ac503213m 25275618

[B245] Wang Y. Cui H. Zhou J. Li F. Wang J. Chen M. (2015). Cytotoxicity, DNA damage, and apoptosis induced by titanium dioxide nanoparticles in human non-small cell lung cancer A549 cells. Environ. Sci. Pollut. Res. 22, 5519–5530. 10.1007/s11356-014-3717-7 25339530

[B246] Wang Y. Wen X. Jia Y. Huang M. Wang F. Zhang X. (2020). Piezo-catalysis for nondestructive tooth whitening. Nat. Commun. 11 (1), 1328. 10.1038/s41467-020-15015-3 32165627 PMC7067860

[B247] Wang Z. Wang G. Kang T. Liu S. Wang L. Zou H. (2021). BiVO(4)/Fe(3)O(4)@polydopamine superparticles for tumor multimodal imaging and synergistic therapy. J. Nanobiotechnology 19 (1), 90. 10.1186/s12951-021-00802-x 33781296 PMC8008624

[B248] Wang W. Zhang X. Ni X. Zhou W. Xie C. Huang W. (2022a). Semiconducting polymer nanoparticles for NIR-II fluorescence imaging-guided photothermal/thermodynamic combination therapy. Biomater. Sci. 10 (3), 846–853. 10.1039/d1bm01646f 35006217

[B249] Wang R. Du N. Jin L. Chen W. Ma Z. Zhang T. (2022b). Hyaluronic acid modified Au@SiO2@Au nanoparticles for photothermal therapy of genitourinary tumors. Polymers 14 (21), 4772. 10.3390/polym14214772 PMC9654671 36365766

[B250] Wang F. Wang B. You W. Chen G. You Y. Z. (2022c). Integrating Au and ZnO nanoparticles onto graphene nanosheet for enhanced sonodynamic therapy. Nano Res. 15 (10), 9223–9233. 10.1007/s12274-022-4599-5 PMC9274620 35845146

[B251] Wang R. Huang Z. Xiao Y. Huang T. Ming J. (2023a). Photothermal therapy of copper incorporated nanomaterials for biomedicine. Biomater. Res. 27 (1), 121. 10.1186/s40824-023-00461-z 38001505 PMC10675977

[B252] Wang M. Xu H. Li T. Li K. Zhang Q. Chen S. (2023b). Sonodynamic therapy of glioblastoma mediated by platelets with ultrasound-triggered drug release. Drug Deliv. 30 (1), 2219429. 10.1080/10717544.2023.2219429 PMC10240984 37264811

[B253] Wang Y. Chang L. Gao H. Yu C. Gao Y. Peng Q. (2024a). Nanomaterials-based advanced systems for photothermal/photodynamic therapy of oral cancer. Eur. J. Med. Chem. 272, 116508. 10.1016/j.ejmech.2024.116508 38761583

[B254] Wang Z. Run Z. Wang H. He X. Li J. (2024b). TiO(2)-Ti(3)C(2) nanocomposites utilize their photothermal activity for targeted treatment of colorectal cancer. Int. J. Nanomedicine 19, 1041–1054. 10.2147/IJN.S446537 38317849 PMC10843984

[B255] Wang H. Zhang T. Zhuang Y. Chen Z. Qu R. Mai Y. (2025a). Calcium/Manganese nanoreactors enable triple-enhanced Chemodynamic/Photodynamic therapy via tumor microenvironment reprogramming. ACS Appl. Mater Interfaces 17 (37), 51854–51865. 10.1021/acsami.5c13514 40919850

[B256] Wang T. Y. An Y. N. Yeh Y. C. (2025b). Ultrasound- and NIR-Responsive Polydextran/Black TiO(2) nanocomposite hydrogels for triple-modal antibacterial therapy. ACS Appl. Mater Interfaces 17 (35), 49910–49929. 10.1021/acsami.5c15341 40827893 PMC12412109

[B257] Wawrzyńczak A. Chudzińska J. Feliczak-Guzik A. (2024). Metal and metal oxides nanoparticles as nanofillers for biodegradable polymers. Chemphyschem 25 (10), e202300823. 10.1002/cphc.202300823 38353297

[B258] Wilhelm S. Tavares A. J. Dai Q. Ohta S. Audet J. Dvorak H. F. (2016). Analysis of nanoparticle delivery to tumours. Nat. Rev. Mater. 1 (5), 16014. 10.1038/natrevmats.2016.14

[B259] Wilhelm M. J. Sharifian Gh. M. Wu T. Li Y. Chang C. M. Ma J. (2021). Determination of bacterial surface charge density via saturation of adsorbed ions. Biophys. J. 120 (12), 2461–2470. 10.1016/j.bpj.2021.04.018 33932437 PMC8390864

[B260] Wiwatchaitawee K. Ebeid K. Quarterman J. C. Naguib Y. Ali M. Y. Oliva C. (2022). Surface modification of nanoparticles enhances drug delivery to the brain and improves survival in a glioblastoma Multiforme Murine model. Bioconjug Chem. 33 (11), 1957–1972. 10.1021/acs.bioconjchem.1c00479 35041398 PMC9662320

[B261] Wood R. J. Lee J. Bussemaker M. J. (2017). A parametric review of sonochemistry: control and augmentation of sonochemical activity in aqueous solutions. Ultrason. Sonochem 38, 351–370. 10.1016/j.ultsonch.2017.03.030 28633836

[B262] Wu H. Lin J. Liu P. Huang Z. Zhao P. Jin H. (2016). Reactive oxygen species acts as executor in radiation enhancement and autophagy inducing by AgNPs. Biomaterials 101, 1–9. 10.1016/j.biomaterials.2016.05.031 27254247

[B263] Wu F. Lin Q. Wang L. Zou Y. Chen M. Xia Y. (2020). A DNA electrochemical biosensor based on triplex DNA-templated Ag/Pt nanoclusters for the detection of single-nucleotide variant. Talanta 207, 120257. 10.1016/j.talanta.2019.120257 31594620

[B264] Wu T. Liu Y. Cao Y. Liu Z. (2022a). Engineering macrophage exosome disguised biodegradable nanoplatform for enhanced sonodynamic therapy of glioblastoma. Adv. Mater 34 (15), e2110364. 10.1002/adma.202110364 35133042

[B265] Wu Y.-N. Yang L. X. Wang P. W. Braet F. Shieh D. B. (2022b). From microenvironment remediation to novel anti-cancer strategy: the emergence of zero valent iron nanoparticles. Pharmaceutics 14, 99. 10.3390/pharmaceutics14010099 PMC8781124 35056996

[B266] Wu N. Tu Y. Fan G. Ding J. Luo J. Wang W. (2022c). Enhanced photodynamic therapy/photothermo therapy for nasopharyngeal carcinoma via a tumour microenvironment-responsive self-oxygenated drug delivery system. Asian J. Pharm. Sci. 17 (2), 253–267. 10.1016/j.ajps.2022.01.002 35582639 PMC9091608

[B267] Wu H. Dong H. Tang Z. Chen Y. Liu Y. Wang M. (2023). Electrical stimulation of piezoelectric BaTiO3 coated Ti6Al4V scaffolds promotes anti-inflammatory polarization of macrophages and bone repair via MAPK/JNK inhibition and OXPHOS activation. Biomaterials 293, 121990. 10.1016/j.biomaterials.2022.121990 36586147

[B268] Wu A. Han M. Ni Z. Li H. Chen Y. Yang Z. (2024). Multifunctional Sr/Se co-doped ZIF-8 nanozyme for chemo/chemodynamic synergistic tumor therapy via apoptosis and ferroptosis. Theranostics 14 (5), 1939–1955. 10.7150/thno.92663 38505601 PMC10945335

[B269] Wu Y. Chen B. Chen X. Zhu G. Du W. Qing L. (2025). Enhanced piezocatalytic therapy of MRSA-infected osteomyelitis using ultrasound-triggered copper nanocrystals-doped barium titanate. Bioact. Mater 51, 450–468. 10.1016/j.bioactmat.2025.04.014 40496625 PMC12149588

[B270] Xia Y. (2016). Optical sensing and biosensing based on non-spherical noble metal nanoparticles. Anal. Bioanal. Chem. 408 (11), 2813–2825. 10.1007/s00216-015-9203-3 26650732

[B271] Xia Y. Guo Y. Yang Z. Chen H. Ren K. Weir M. D. (2019). Iron oxide nanoparticle-calcium phosphate cement enhanced the osteogenic activities of stem cells through WNT/β-catenin signaling. Mater Sci. Eng. C Mater Biol. Appl. 104, 109955. 10.1016/j.msec.2019.109955 31500064

[B272] Xiang H. Lin H. Yu L. Chen Y. (2019). Hypoxia-Irrelevant photonic thermodynamic cancer nanomedicine. ACS Nano 13 (2), 2223–2235. 10.1021/acsnano.8b08910 30624041

[B273] Xiao L. Feng M. Chen C. Xiao Q. Cui Y. Zhang Y. (2023). Microenvironment-Regulating drug delivery nanoparticles for treating and preventing typical biofilm-induced oral diseases. Adv. Mater. 37, 2304982. 10.1002/adma.202304982 37875431

[B274] Xiong Y. Rao Y. Hu J. Luo Z. Chen C. (2023). Nanoparticle-Based photothermal therapy for breast cancer noninvasive treatment. Adv. Mater 37, e2305140. 10.1002/adma.202305140 37561994

[B275] Xu Y. Karmakar A. Heberlein W. E. Mustafa T. Biris A. R. Biris A. S. (2012). Multifunctional magnetic nanoparticles for synergistic enhancement of cancer treatment by combinatorial radio frequency thermolysis and drug delivery. Adv. Healthc. Mater 1 (4), 493–501. 10.1002/adhm.201200079 23184783

[B276] Xu Y. Wang J. Li X. Liu Y. Dai L. Wu X. (2014). Selective inhibition of breast cancer stem cells by gold nanorods mediated plasmonic hyperthermia. Biomaterials 35 (16), 4667–4677. 10.1016/j.biomaterials.2014.02.035 24630839

[B277] Xu P. Y. Kumar Kankala R. Wang S. B. Chen A. Z. (2023). Sonodynamic therapy-based nanoplatforms for combating bacterial infections. Ultrason. Sonochem 100, 106617. 10.1016/j.ultsonch.2023.106617 37769588 PMC10542942

[B278] Xu K. Zou Y. Lin C. Zhang L. Tan M. Li M. (2024). Cascade catalysis nanozyme for interfacial functionalization in combating implant infections associated with diabetes via sonodynamic therapy and adaptive immune activation. Biomaterials 311, 122649. 10.1016/j.biomaterials.2024.122649 38850718

[B279] Yan Z. Wang D. Gao Y. (2023). Nanomaterials for the treatment of bacterial infection by photothermal/photodynamic synergism. Front. Bioeng. Biotechnol. 11, 1192960. 10.3389/fbioe.2023.1192960 37251578 PMC10210152

[B280] Yang Y. Gao N. Hu Y. Jia C. Chou T. Du H. (2015a). Gold nanoparticle-enhanced photodynamic therapy: effects of surface charge and mitochondrial targeting. Ther. Deliv. 6 (3), 307–321. 10.4155/tde.14.115 25853307

[B281] Yang H. He L. Q. Hu Y. W. Lu X. Li G. R. Liu B. (2015b). Quantitative detection of photothermal and photoelectrocatalytic effects induced by SPR from Au@Pt nanoparticles. Angew. Chem. Int. Ed. Engl. 54 (39), 11462–11466. 10.1002/anie.201505985 26278278

[B282] Yang C. Zhu Y. Li D. Liu Y. Guan C. Man X. (2021). Red Phosphorus decorated TiO(2) nanorod mediated photodynamic and photothermal therapy for renal cell carcinoma. Small 17 (30), e2101837. 10.1002/smll.202101837 34145768

[B283] Yang F. Xue J. Wang G. Diao Q. (2022). Nanoparticle-based drug delivery systems for the treatment of cardiovascular diseases. Front. Pharmacol. 13, 999404. 10.3389/fphar.2022.999404 36172197 PMC9512262

[B284] Yang G. Pan X. Feng W. Yao Q. Jiang F. Du F. (2023). Engineering Au(44) nanoclusters for NIR-II luminescence imaging-guided photoactivatable cancer immunotherapy. ACS Nano 17 (16), 15605–15614. 10.1021/acsnano.3c02370 37503901

[B285] Yilmazer A. Eroglu Z. Gurcan C. Gazzi A. Ekim O. Sundu B. (2023). Synergized photothermal therapy and magnetic field induced hyperthermia via bismuthene for lung cancer combinatorial treatment. Mater Today Bio 23, 100825. 10.1016/j.mtbio.2023.100825 37928252 PMC10622883

[B286] Yim G. Kang S. Kim Y. J. Kim Y. K. Min D. H. Jang H. (2019). Hydrothermal galvanic-replacement-tethered synthesis of Ir-Ag-IrO(2) nanoplates for computed tomography-guided multiwavelength potent thermodynamic cancer therapy. ACS Nano 13 (3), 3434–3447. 10.1021/acsnano.8b09516 30860814

[B287] Yoo S. W. Oh G. Ahn J. C. Chung E. (2021). Non-oncologic applications of nanomedicine-based phototherapy. Biomedicines 9 (2), 113. 10.3390/biomedicines9020113 PMC7911939 33504015

[B288] You Y. Jiang J. Zheng G. Chen Z. Zhu Y. Ma H. (2024). *In situ* piezoelectric-catalytic anti-inflammation promotes the rehabilitation of acute spinal cord injury in synergy. Adv. Mater. 36 (18), 2311429. 10.1002/adma.202311429 38298173

[B289] Younis N. S. Bakir E. M. Mohamed M. E. El Semary N. A. (2019). Cyanobacteria as nanogold factories II: chemical reactivity and anti-Myocardial infraction properties of customized gold nanoparticles biosynthesized by cyanothece sp. Mar. Drugs 17 (7), 402. 10.3390/md17070402 31288394 PMC6669522

[B290] Younis A. B. Haddad Y. Kosaristanova L. Smerkova K. (2023). Titanium dioxide nanoparticles: recent progress in antimicrobial applications. WIREs Nanomedicine Nanobiotechnology 15 (3), e1860. 10.1002/wnan.1860 36205103

[B291] Yu M. Lei B. Gao C. Yan J. Ma P. X. (2017). Optimizing surface-engineered ultra-small gold nanoparticles for highly efficient miRNA delivery to enhance osteogenic differentiation of bone mesenchymal stromal cells. Nano Research 10 (1), 49–63. 10.1007/s12274-016-1265-9

[B292] Yu P. Zheng L. Wang P. Chai S. Zhang Y. Shi T. (2020). Development of a novel polysaccharide-based iron oxide nanoparticle to prevent iron accumulation-related osteoporosis by scavenging reactive oxygen species. Int. J. Biol. Macromol. 165, 1634–1645. 10.1016/j.ijbiomac.2020.10.016 33049237

[B293] Yu Y. Wu S. Zhang L. Xu S. Dai C. Gan S. (2022). Cationization to boost both type I and type II ROS generation for photodynamic therapy. Biomaterials 280, 121255. 10.1016/j.biomaterials.2021.121255 34810034

[B294] Yu Q. Shi W. Li S. Liu H. Zhang J. (2023). Emerging advancements in piezoelectric nanomaterials for dynamic tumor therapy. Molecules 28 (7), 3170. 10.3390/molecules28073170 37049933 PMC10095813

[B295] Yumita N. Nishigaki R. Umemura K. Umemura S. (1989). Hematoporphyrin as a sensitizer of cell-damaging effect of ultrasound. Jpn. J. Cancer Res. 80 (3), 219–222. 10.1111/j.1349-7006.1989.tb02295.x 2470713 PMC5917717

[B296] Yuwen L. Liu Y. Xu F. Zhang C. Chen X. Yin Z. (2025). Fe(3)O(4)/MnCO(3) microbubbles for efficient elimination of bacterial biofilms by mechanical/sonodynamic effects under ultrasound irradiation and magnetic field targeting. Biomater. Sci. 13 (12), 3367–3379. 10.1039/d5bm00227c 40354102

[B297] Zaghi S. Acar M. Hultgren B. Boggio P. S. Fregni F. (2010). Noninvasive brain stimulation with low-intensity electrical currents: putative mechanisms of action for direct and alternating current stimulation. Neuroscientist 16 (3), 285–307. 10.1177/1073858409336227 20040569

[B298] Zahraie N. Perota G. Dehdari Vais R. Sattarahmady N. (2022). Simultaneous chemotherapy/sonodynamic therapy of the melanoma cancer cells using a gold-paclitaxel nanostructure. Photodiagnosis Photodyn. Ther. 39, 102991. 10.1016/j.pdpdt.2022.102991 35779857

[B299] Ze Y. Sheng L. Zhao X. Ze X. Wang X. Zhou Q. (2014). Neurotoxic characteristics of spatial recognition damage of the hippocampus in mice following subchronic peroral exposure to TiO2 nanoparticles. J. Hazardous Materials 264, 219–229. 10.1016/j.jhazmat.2013.10.072 24295774

[B300] Zhang S. Gao H. Bao G. (2015). Physical principles of nanoparticle cellular endocytosis. ACS Nano 9 (9), 8655–8671. 10.1021/acsnano.5b03184 26256227 PMC5681865

[B301] Zhang H. Li Y. H. Chen Y. Wang M. M. Wang X. S. Yin X. B. (2017). Fluorescence and magnetic resonance dual-modality imaging-guided photothermal and photodynamic dual-therapy with magnetic porphyrin-metal organic framework nanocomposites. Sci. Rep. 7, 44153. 10.1038/srep44153 28272454 PMC5341151

[B302] Zhang L. Wu L. Mi Y. Si Y. (2019). Silver nanoparticles induced cell apoptosis, membrane damage of Azotobacter vinelandii and Nitrosomonas europaea via generation of reactive oxygen species. Bull. Environ. Contam. Toxicol. 103 (1), 181–186. 10.1007/s00128-019-02622-0 31049596

[B303] Zhang L. Cheng Q. Li C. Zeng X. Zhang X. Z. (2020). Near infrared light-triggered metal ion and photodynamic therapy based on AgNPs/porphyrinic MOFs for tumors and pathogens elimination. Biomaterials 248, 120029. 10.1016/j.biomaterials.2020.120029 32289589

[B304] Zhang T. Zheng Q. Fu Y. Xie C. Fan G. Wang Y. (2021). α-Fe(2)O(3)@Pt heterostructure particles to enable sonodynamic therapy with self-supplied O(2) and imaging-guidance. J. Nanobiotechnology 19 (1), 358. 10.1186/s12951-021-01105-x 34736483 PMC8569996

[B305] Zhang M. Hu W. Cai C. Wu Y. Li J. Dong S. (2022a). Advanced application of stimuli-responsive drug delivery system for inflammatory arthritis treatment. Mater Today Bio 14, 100223. 10.1016/j.mtbio.2022.100223 PMC8881671 35243298

[B306] Zhang S. Yi K. Chen A. Shao J. Peng L. Luo S. (2022b). Toxicity of zero-valent iron nanoparticles to soil organisms and the associated defense mechanisms: a review. Ecotoxicology 31 (6), 873–883. 10.1007/s10646-022-02565-z 35834074

[B307] Zhang L. Forgham H. Shen A. Qiao R. Guo B. (2022c). Recent advances in single Fe-Based nanoagents for photothermal-chemodynamic cancer therapy. Biosens. (Basel) 12 (2), 86. 10.3390/bios12020086 PMC8869282 35200346

[B308] Zhang J. Liang C. Wei Z. Yang W. Ge W. Qu X. (2022d). TME-triggered MnSiO(3)@Met@GOx nanosystem for ATP dual-inhibited starvation/chemodynamic synergistic therapy. Biomaterials 287, 121682. 10.1016/j.biomaterials.2022.121682 35870264

[B309] Zhang W. Li J. Chen L. Chen H. Zhang L. (2023). Palladium-based multifunctional nanoparticles for combined chemodynamic/photothermal and calcium overload therapy of tumors. Colloids Surf. B Biointerfaces 230, 113529. 10.1016/j.colsurfb.2023.113529 37708713

[B310] Zhang X. Zhang Z. Yuan H. Sun X. (2024a). ZnO quantum dots decorated BaTiO(3) for cancer sonodynamic therapy. Ultrason. Sonochem 110, 107036. 10.1016/j.ultsonch.2024.107036 39191130 PMC11396363

[B311] Zhang E. Shotbolt M. Chang C. Y. Scott-Vandeusen A. Chen S. Liang P. (2024b). Controlling action potentials with magnetoelectric nanoparticles. Brain Stimul. 17 (5), 1005–1017. 10.1016/j.brs.2024.08.008 39209064

[B312] Zhang Y. Yang Y. Feng Y. Gao X. Pei L. Li X. (2024c). Sonodynamic therapy for the treatment of atherosclerosis. J. Pharm. Anal. 14 (5), 100909. 10.1016/j.jpha.2023.11.016 PMC11127226 38799235

[B313] Zhang S. Gao X. J. Ma Y. Song K. Ge M. Ma S. (2024d). A bioinspired sulfur-Fe-heme nanozyme with selective peroxidase-like activity for enhanced tumor chemotherapy. Nat. Commun. 15 (1), 10605. 10.1038/s41467-024-54868-w PMC11621791 39638998

[B314] Zhang Y. Liu Y. Zhao M. Wang Y. Yi H. Liu D. (2025). Iron-based theranostic nanoenzyme for combined tumor magneto-photo thermotherapy and starvation therapy. Biomater. Adv. 166, 214038. 10.1016/j.bioadv.2024.214038 39306963

[B315] Zhao D. Sun X. Tong J. Ma J. Bu X. Xu R. (2012). A novel multifunctional nanocomposite C225-conjugated Fe3O4/Ag enhances the sensitivity of nasopharyngeal carcinoma cells to radiotherapy. Acta Biochim. Biophys. Sin. (Shanghai) 44 (8), 678–684. 10.1093/abbs/gms051 22710262

[B316] Zhao L. L. Zhang Y. Wang F. Hu S. Wang X. Ma B. (2017). *BaTiO* _3_ *nanocrystal-mediated micro pseudo-electrochemical cells with ultrasound-driven piezotronic enhancement for polymerization* . Nano ENERGY 39, 461–469. 10.1016/j.nanoen.2017.07.037

[B317] Zhao S. Luo Y. Chang Z. Liu C. Li T. Gan L. (2021). BSA-Coated gold nanorods for NIR-II photothermal therapy. Nanoscale Res. Lett. 16 (1), 170. 10.1186/s11671-021-03627-7 34842995 PMC8630206

[B318] Zhao J. Gao N. Xu J. Zhu X. Ling G. Zhang P. (2023). Novel strategies in melanoma treatment using silver nanoparticles. Cancer Lett. 561, 216148. 10.1016/j.canlet.2023.216148 36990267

[B319] Zheng H. Yin N. Lv K. Niu R. Zhou S. Wang Y. (2024). Defect-rich sonosensitizers based on CeO(2) with Schottky heterojunctions for boosting sonodynamic/chemodynamic synergistic therapy. J. Mater Chem. B 12 (17), 4162–4171. 10.1039/d4tb00084f 38619400

[B320] Zhou R. Zhang M. Xi J. Li J. Ma R. Ren L. (2022a). Gold nanorods-based photothermal therapy: interactions between biostructure, nanomaterial, and near-infrared irradiation. Nanoscale Res. Lett. 17 (1), 68. 10.1186/s11671-022-03706-3 PMC9325935 35882718

[B321] Zhou K. Zhang Z. Xue J. Shang J. Ding D. Zhang W. (2022b). Hybrid Ag nanoparticles/polyoxometalate-polydopamine nano-flowers loaded chitosan/gelatin hydrogel scaffolds with synergistic photothermal/chemodynamic/Ag+ anti-bacterial action for accelerated wound healing. Int. J. Biol. Macromol. 221, 135–148. 10.1016/j.ijbiomac.2022.08.151 36029962

[B322] Zhu P. Chen Y. Shi J. (2020). Piezocatalytic Tumor therapy by ultrasound-triggered and BaTiO(3) -Mediated piezoelectricity. Adv. Mater 32 (29), e2001976. 10.1002/adma.202001976 32537778

[B323] Zhu S. Yan F. Yang L. Li B. Xue R. Yu W. (2023). Low-dose X-ray radiodynamic therapy solely based on gold nanoclusters for efficient treatment of deep hypoxic solid tumors combined with enhanced antitumor immune response. Theranostics 13 (3), 1042–1058. 10.7150/thno.78649 PMC9925321 36793856

[B324] Zhu Y. Niu X. Ding C. Lin Y. Fang W. Yan L. (2024). Carrier-Free self-assembly nano-sonosensitizers for Sonodynamic-Amplified Cuproptosis-Ferroptosis in glioblastoma therapy. Adv. Sci. (Weinh) 11 (23), e2402516. 10.1002/advs.202402516 38582500 PMC11187904

